# A community intervention effectiveness study of single dose or two doses of bivalent HPV vaccine (CERVARIX®) in female school students in Thailand

**DOI:** 10.1371/journal.pone.0267294

**Published:** 2022-04-28

**Authors:** Suchada Jiamsiri, Chulwoo Rhee, Hyeon Seon Ahn, Nimesh Poudyal, Hyeong-Won Seo, Worrawan Klinsupa, Pornjarim Nilyanimit, Nakorn Premsri, Chawetsan Namwat, Sompong Vonpunsawad, Yun Chon, Sunju Park, Deok-Ryun Kim, Elizabeth R. Unger, Lauri Markowitz, Yong Poovorawan, Supachai Rerks-Ngarm, Jean Louis Excler, Julia Lynch

**Affiliations:** 1 Division of Vaccine Preventable Diseases, Department of Disease Control, Ministry of Public Health, Nonthaburi, Thailand; 2 International Vaccine Institute, Seoul, Republic of Korea; 3 Center of Excellence in Clinical Virology, Department of Pediatrics, Faculty of Medicine, Chulalongkorn University, Bangkok, Thailand; 4 National Vaccine Institute, Ministry of Public Health, Nonthaburi, Thailand; 5 Bureau of Epidemiology, Department of Disease Control, Ministry of Public Health, Nonthaburi, Thailand; 6 Chronic Viral Diseases Branch, Division of High-Consequence Pathogens and Pathology, National Center for Emerging and Zoonotic Infectious Diseases, US Centers for Disease Control and Prevention, Atlanta, GA, United States of America; 7 Viral Vaccine Preventable Diseases Branch, Division of Viral Diseases, National Center for Immunization and Respiratory Diseases, US Centers for Disease Control and Prevention, Atlanta, GA, United States of America; Public Library of Science, UNITED KINGDOM

## Abstract

Human papillomavirus (HPV) is a common infection principally spread through sexual activity. Most HPV infections are asymptomatic and resolve spontaneously. However, persistent infection may progress to cervical cancer. Highly efficacious HPV vaccines have been available since 2006, yet uptake into national programs has been slow in part due to cost. WHO guidelines call for a two-dose (0,6 month) schedule for girls 9–14 years of age. Post-hoc analyses of randomized trials have found high vaccine effectiveness following a single dose of vaccine. In order to provide additional data on the potential impact of single dose HPV vaccination in a real-world setting, we are conducting an effectiveness study among Thai schoolgirls. This is an observational study of a single dose (SD) or two doses (2D) of the bivalent HPV vaccine CERVARIX® (GlaxoSmithKline plc.) administered in a school-based program to 8–9,000 Grade 8 female students in two provinces of Thailand beginning in 2018; one province is assigned the SD, and the other the standard 2D regimen. The reduction in HPV vaccine-type prevalence will be assessed in each province two and four years after vaccination by comparing HPV prevalence in urine samples obtained through cross-sectional surveys of the immunized grade cohort as they age and compared to a historical “baseline” HPV prevalence of same age students.

## Introduction

Human Papillomavirus (HPV) infection of the reproductive tract is common, principally spread through sexual activity. The majority of HPV infections are asymptomatic and resolve spontaneously. However, persistent infection with specific HPV types (most frequently HPV 16 and 18) may lead to precancerous lesions which if untreated may progress to cervical and some other cancers [1]. In 2020 it was estimated that 604,000 new cases of cervical cancers occurred in women with over 340,000 cervical cancer related deaths [2]. As a result of limited availability of screening and treatment, 85% of the deaths occur in resource-limited settings.

All available HPV vaccines are virus-like particle (VLP) vaccines and have been shown to have high efficacy in randomized controlled trials [3]. CERVARIX® (GlaxoSmithKline) and Gardasil® (Merck & Co) received WHO prequalification in 2009, and Gardasil®9, in 2018. Cecolin® (Xiamen Innovax Biotech Co) was recently licensed in China and currently under review for World Health Organization (WHO) prequalification. HPV vaccines are ideally administered prior to the initiation of sexual activity. Although originally licensed based on a three-dose schedule (0, 1–2, 6 months) in young adult women, in 2014 the WHO guidelines recommended a two-dose regimen (0, 6 months) for girls aged 9–14 years based on the non-inferiority of antibody responses of the two-dose schedule in that age group as compared to the three-dose schedule efficacious among women [4, 5].

Despite reductions in the cost of these vaccines through the Global Alliance for Vaccine and Immunization (GAVI) or tiered-pricing, the uptake of HPV vaccines into national programs has been slow particularly in low- and middle-income countries (LMIC) [6]. Barriers to uptake include cost (vaccine and delivery cost), and programmatic challenges (novel age of target group, competing new vaccine introduction priorities) and vaccine availability. Thailand introduced HPV vaccine into a nationwide school-based program in 2017, targeting girls in Grade 5. However, catch up immunization programs among school girls older than Grade 5 was stalled due to limited funding.

Post-hoc analyses of randomized trials have found high vaccine effectiveness following a single dose (SD) of bivalent or quadrivalent vaccine [7–9]. However, the interpretation of these analyses is limited by several factors including women with incomplete vaccination schedules not randomized by number of doses, small sample size, and low number of incident or persistent infections.

SD HPV vaccination would enable twice as many female students to be covered with the same amount of vaccine supply, facilitate catch-up vaccination campaigns, and alleviate the overall economic cost of vaccine supply and delivery, while averting treatment costs of disease. WHO acknowledges that more data are needed to issue such a recommendation [6].

In order to provide additional data on the potential impact of SD HPV vaccination in a real-world setting, we are conducting an effectiveness study of the SD and the standard two-dose (2D) regimens in two provinces in Thailand. Vaccination is administered in schools, mirroring the already implemented national Grade 5 HPV immunization program, but targeting Grade 8 females outside the grade eligibility.

### Objectives

Primary objectives

- Demonstrate effectiveness of SD HPV vaccination by a reduction in vaccine-type HPV prevalence (HPV16 and/or 18) at two and four years post vaccination compared to the prevalence among unvaccinated same grade female students collected in a baseline survey.
- Demonstrate effectiveness of SD and 2D HPV vaccine regimens are similar by comparing reductions in vaccine HPV-type prevalence at four years post vaccination compared with the baseline prevalence in the two provinces.

Secondary objectives

- Estimate prevalence of HPV infection in female students in high school Grades 10 and 12 (G10, G12) and vocational school years 1 and 3 (V1, V3)
- Estimate the distribution of HPV types detected
- Assess HPV type-specific antibody response pre- and post-vaccination in a subset of participants

Exploratory objectives

- Assess comparability of sexual behavior between the two provinces among female students in Grades 8, 10 and 12 (G8, G10, G12) and vocational school years 1 and 3 (V1, V3)
- Assess comparability of risk for HPV infection between G10/V1 and G12/V3 female students at baseline and at years 2 and 4 post vaccination
- Assess possible herd protection conferred by the HPV vaccination in unvaccinated female students

## Methods and analysis

### Ethics and inclusivity

The protocol was first approved by the IVI IRB (IVI HPV1 2018–005 V1.3) on 20 Nov 2018, the Thailand Ministry of Health EC (Ref.no.25/2561, V1.0) on 29 Nov 2018, and the Chulalongkorn University IRB (IRB No. 495/61 V1.0) on 4 Dec 2018. The US CDC IRB deferred to the IVI IRB 7 May 2019. This paper is based on the protocol version 6.0 dated 15 Dec 2021 S1 File. Any amendment of the approved protocol shall be submitted for review and approval by the competent authorities in Thailand and at IVI. Written informed consent and/or assent is obtained from all guardians and subjects. The trial is registered at clinicaltrials.gov with the registry name **“**Effectiveness of Single Dose or Two Doses of Bivalent HPV Vaccine in Thailand (IVIHPV1)**”** and identifier NCT03747770. Additional information regarding the ethical, cultural, and scientific considerations specific to inclusivity in global research is included in the S2 File. During study design development, Thai medical leaders not otherwise involved in the study and representing pediatrics, gynecology, oncology and public health departments were invited to a stakeholder meeting to discuss the study plan and objectives and gain their input. These were further discussed with public health and education leaders within the provinces prior to finalization of the study plan and protocol.

### Study design

This is an observational community effectiveness study of SD or 2D of CERVARIX® (containing HPV16 and HPV18) administered in a voluntary school-based program to Grade 8 female students less than age 15 years in two provinces of Thailand, Udon Thani and Buri Ram. The overall study is divided into four independent parts:

- Part A–Grade 8 vaccination and sexual behavior questionnaire (SBQ)
- Part B–Baseline cross-sectional survey (CSS) of HPV prevalence based on self-collected urine samples and SBQ (G10/V1 and G12/V3)
- Part C–CSS of HPV prevalence based on self-collected urine samples and SBQ two years after vaccination (G10/V1)
- Part D–CSS of HPV prevalence based on self-collected urine samples and SBQ four years after vaccination (G12/V3)

Vaccine effectiveness will be assessed as the reduction in vaccine HPV-type infection measured in a CSS two and four years after vaccination, compared to the Baseline CSS. A subset of students in each province (N = 200) will be invited to participate in a sub-study to evaluate immune response to vaccination. Table 1 summarizes study components, expected timeline and sample size.

**Table 1:** Study components, timeline, and sample size per study activity.

Study Component	Timeline	Sample Size	Intervention/Activity
**Part A: Vaccination**	2018 Dec-2019 Feb	All eligible 8,000~9,000* schoolgirls (<15 years old) in Grade 8 per province	Udon Thani: single-dose HPV vaccine regimen Buri Ram: two-dose HPV vaccine regimen 6 months apart
		N = 1,500 for SBQ	
		N = 200 (pre-vaccine serology)	
**Part B: Baseline CSS**	2018 Dec-2019 Feb	G10/V1: N = 2,600 schoolgirls per province	SBQ and HPV urine prevalence
		G12/V3: N = 2,000 schoolgirls per province	
**Part C: Year 2 CSS**	2020 Dec-2021 Feb	G10/V1: N = 2,600 schoolgirls per province	SBQ and HPV urine prevalence
		N = 200 (serology)	
**Part D: Year 4 CSS**	2022 Dec-2023 Feb	G12/V3: N = 2,000 schoolgirls per province	SBQ and HPV urine prevalence
		N = 200 (serology)	

*Approximate number of Grade 8 schoolgirls that will be eligible and offered vaccination

### Study setting

This study will be conducted in two provinces in North-East Thailand, Udon Thani and Buri Ram, selected by the Ministry of Public Health based on the similarity of criteria such as size of the female student population, socio-economic parameters, comparability of self-reported sexual activity in a national survey, logistics feasibility, and high degree of acceptance of the Grade 5 HPV vaccine program by local authorities and communities. Each province is further sub-divided into Districts, each with a District Hospital and subordinate Health Centers (Table 2). This public health infrastructure is used to deliver school-based vaccinations including the Grade 5 HPV vaccination program and Grade 6 diphtheria and tetanus boosters. This infrastructure and experience will be leveraged to conduct the Grade 8 HPV vaccination campaign for this study over a three-month period. After Grade 9, students are tracked into either regular high school to complete Grades 10–12 (G10-G12) or vocational schools to complete grades vocational year 1–3 (V1-V3). Concurrent with the vaccination campaign, MOPH and school-based staff will conduct the Baseline CSS at the high schools and vocational schools. By random selection, the 2D and SD regimens were allocated to Buri Ram and Udon Thani, respectively.

**Table 2:** Number of districts, district hospitals and schools by province.

Province	Number of Districts	Number of District Hospitals	Number of all schools participating in the study
**Udon Thani**	20	194	349
**Buri Ram**	23	215	369

### Study component recruitment and enrollment

All aspects of recruitment and enrollment will be conducted according to ICH E6(R2) GCP guidelines. For each of the activities described below, eligibility is assessed according to the inclusion and exclusion criteria (Table 3). Parents or guardian are informed about the activity in their own language by school staff and MOPH study staff and offered an opportunity to ask questions.

**Table 3:** Inclusion and exclusion criteria for each study component.

Study Component	Inclusion criteria	Exclusion criteria
**Grade 8 Vaccination**	• Female students with identification card • Less than 15 years of age • Parent or guardian consent for vaccination and blood collection as applicable • Participant assent for vaccination, SBQ, and blood collection as applicable	• Students who already received HPV vaccination • Reported pregnancy • Any student who has a preexisting known medical condition or diagnosed psychological illness which in the opinion of the Principal Investigator or designee may be detrimental to her wellbeing
**Baseline CSS**	• Female students with identification card • Participant assent for SBQ and urine collection	• Any student who has a preexisting known medical condition or diagnosed psychological illness which in the opinion of the Principal Investigator or designee may be detrimental to her well-being
**Year 2 and Year 4 post-vaccination CSS**	• Female students with identification card • Parent or guardian consent for blood collection as applicable • Participant assent for SBQ, urine, and blood collection from those vaccinated at Grade 8 as applicable	• Any student who has a preexisting known medical condition or diagnosed psychological illness which in the opinion of the Principal Investigator or designee may be detrimental to her wellbeing

CSS: Cross-sectional surveys, SBQ: Sexual behavior questionnaire

#### Vaccination

Open label vaccination with either SD (Udon Thani) or 2D regimen (Buri Ram) are offered to all Grade 8 students younger than age 15 years between December 2018-February 2019 on a voluntary basis. A subset of 1500 students in each province are selected by a proportional and systematic sampling method and invited to complete the SBQ. For Grade 8 students less than 15 years of age, both a parental consent and student assent form is completed prior to enrollment for vaccination, and a separate assent form is obtained for the SBQ.

#### Cross-sectional surveys

Cross-sectional surveys (CSS) are intended to sample a representative subset of students in the province, and all schools willing to participate are included in the surveys. As sexual activity is expected to be higher among vocational students (based on existing national survey data), and the overall prevalence of vaccine-type HPV infections is expected to be low, vocational students will be over sampled to achieve ~50% of all enrolled subjects. Based on the overall province and school-type sample size, school-specific target enrollments will be calculated to be proportionate to their grade enrollments. At each school, students will be selected to participate in the CSS based on a systematic sampling from an eligibility list. When selected students are either not eligible because of exclusion criteria or decide not to participate in the study, additional students are selected by the field staff using the same method until the school target enrollment is reached. If the school target enrollment cannot be met, study staff can over enroll at another school of the same type in order to meet the province target enrollment. In order to ensure student confidentiality, only student assent is required for enrollment in the CSS and SBQ. In the Year 2 and Year 4 CSS, the student vaccination status is verified and documented at the time of enrollment.

#### Serology

For practical purposes, a non-random subset of 200 Grade 8 students in each province consenting to vaccination and living in close proximity of a District Hospital will be invited to participate in this sub-study. A blood sample will be taken prior to vaccination and at the time of the Year 2 and Year 4 CSS. Eligibility criteria are re-assessed by District Health staff at the time of blood sample collection. Both parental consent and student assent forms are completed for each blood draw prior to vaccination, and at G10/V1 and G12/V3.

#### Withdrawal

Parents or guardians, and participants are informed that participation in each activity is voluntary and that they are free to withdraw their consent or assent at any time upon request, without justification and without prejudice. Subjects who withdraw may request destruction of samples and deletion of their data. The Principal Investigator may also discontinue participation in case of any occurrence that in her/his judgment might be detrimental to the participants well-being, and the reason for their withdrawal will be recorded.

### Interventions

CERVARIX® is a bivalent HPV vaccine manufactured by GlaxoSmithKline plc. and presented as a suspension containing purified viral L1 protein for HPV types 16 and 18 and administered by intramuscular injection. It is produced using a baculovirus expression system. Each 0.5 mL dose of the bivalent vaccine contains 20 μg of HPV16 L1 protein and 20 μg of HPV18 L1 protein formulated with AS04 (containing 500 μg of aluminum hydroxide and 50 μg of 3-O-desacyl-4-monophosphoryl lipid A.

This vaccine is indicated for use in females and males from the age of 9 years for the prevention of premalignant anogenital lesions affecting the cervix, vulva, vagina and anus, and cervical and anal cancers causally related to specific HPV types [10, 11]. Randomized clinical trials in Thailand have shown the vaccine is safe and immunogenic [12, 13]. No serious adverse reactions have been recorded since the nationwide implementation of the 2D-regimen in Grade 5 (MOPH/DDC Epidemiology Department, Thailand).

### Specific assessments

#### Vaccination adverse event monitoring

Post vaccination monitoring of the Grade 8 subjects is conducted as in other school-based programs and adverse events following immunization (AEFI) recorded through the national surveillance system and reported as per national guidelines. The investigators will exercise due diligence in ascertaining, and accurately recording all reportable AEFI that vaccinated study participants may experience. Reported AEFI are linked to the study ID of the students. AEFI may also be reported to the MOPH EC as per local regulations.

#### CSS assessment of HPV infection

Urine samples are self-collected by the students at school using a commercially available urine collection device, Colli-Pee® (Novosanis, Belgium; FV-5000 series N00176) pre-labeled with coded subject ID. The device contains 7 ml of UCM preservative and collects up to 20 ml of the initial void of urine. This collection method has been successfully used in Bhutan and Rwanda for HPV prevalence assessments, and demonstrated a high degree of comparability to physician-collected cervical swabs in a pilot study in Thailand [14–18]. Urine samples are then stored at 2–8°C within 30 minutes and transported via monitored cold chain to Chulalongkorn University lab in Bangkok for further processing within 48 hours of reception. A 10 ml aliquot of urine is centrifuged, 9 ml of supernatant removed and the cell pellet re-suspended in remaining one ml and frozen at -20°C until HPV DNA testing. A second aliquot of urine is frozen at -20°C for further testing, if required.

Cobas® (Roche), a high throughput, qualitative assay system will be used for detection of HPV16 and 18 and 12 other HPV types (31, 33, 35, 39, 45, 51, 52, 56, 58, 59, 66, 68) [19–21]. Residual extracts from all Cobas-positive samples and equal numbers of Cobas-negative samples will be frozen -20°C and subsequently assayed with Anyplex™ (Seegene) to identify 28 HPV types [22, 23]. Cobas® and Anyplex™ testing will be initiated in parallel with field collection activities and continue until all samples from the activity year are tested. Test results will be uploaded to the electronic data capture (EDC) system by Chulalongkorn lab staff.

#### Serology

The purpose of the immunogenicity study is to verify that the vaccine administered is immunogenic for HPV16 and 18 and to assess response to non-vaccine types (HPV 6, 11, 31, 33, 45, 52, 58) with potential cross-immunogenicity. Blood (5 ml) will be collected via antecubital venipuncture by trained phlebotomist into serum tubes and allowed to clot at room temperature. Sera will be aliquoted into 1 ml cryovials and stored at -70°C and shipped on dry ice to the US CDC laboratory for testing using multiplex direct IgG ELISA against L1/L2 HPV virus-like particles on the Meso Scale Discovery platform with chemiluminescent detection as described with minor modifications [24].

### Analysis

#### Primary outcome

The primary outcome is the vaccine effectiveness, or reduction in vaccine-type HPV prevalence (HPV16 and/or 18) as measured by Cobas® in a single time point urine sample collected through a CSS two and four years after vaccination compared to unvaccinated same grade female students from the Baseline CSS in each of the provinces. Four years after vaccination a non-inferiority comparison of SD and 2D vaccine effectiveness will be made in the SD and 2D provinces. As the analysis will use a non-contemporaneous control group (Baseline survey), and the composition of the groups and/or their sexual behavior may differ, an adjusted analysis will be conducted to account for imbalances in the populations between the two time points within the province, and between the provinces. The main indicator for imbalance in sexual behavior will be the prevalence of 7 non-vaccine, non-cross-reactive high-risk HPV types (35, 39, 56, 58, 59, 66, 68).

#### Sample size and power calculations

The assumed prevalence of HPV16 and/or 18 in G10/V1 and G12/V3 female students in the two provinces for Year 2 and Year 4 CSS are 2% and 3%, respectively. Due to lack of data on HPV prevalence in young female students in Thailand and the uncertainty of our assumption, the sample size for Year 2 and Year 4 CSS will be re-assessed based on the actual prevalence of HPV infection in G10/V1 and G12/V3 from the Baseline CSS prior to the Year 2 CSS. Assuming a 90% vaccine coverage of Grade 8 students, the sample size of N = 2600 per province at Year 2, and N = 2,000 per province at Year 4 are calculated to provide >80% power to show the vaccine effectiveness of SD or 2D is greater than 50% using one-sided test at a 0.025 significance. At four years post vaccination, this sample size is calculated to provide 80% power to demonstrate the VE of SD is non-inferior to VE of 2D with non-inferiority margin of 10% using one-sided test at 0.025 significance level.

For the assessment of antibody response to the vaccine, the sample size N = 200 per province is based on keeping the coefficient of variation (CV) of mean titer of HPV16 or HPV18 less than 2.5, so that the geometric mean titer (GMT) estimate can be compared between provinces.

#### Data collection

*Vaccination*. A limited amount of data is collected on a field source document including name, national ID, date of birth and date of vaccination. Each student agreeing to vaccination (or any other study activity) is assigned a pre-printed subject number in numerical and bar-code format which codes for province, school, a unique number, grade, and activity type (e.g., vaccination, CSS, serology). The pre-printed barcodes are affixed to the consent and assent documents and the source document, which are maintained only by the MOPH staff. Only the complete subject number, date of birth, date of consent or assent and date of vaccination are entered into the EDC system by study staff.

*Cross-sectional surveys*. Each student’s assigned subject number in numerical and bar-code format on pre-printed barcodes is affixed to documents and urine sample containers. The SBQ is a web-based, self-administered questionnaire with a limited set of questions derived from the MOPH national survey regarding sexual behavior. Using their own cell phone or via a tablet or phone provided by study staff, students scan their assigned subject number barcode at a weblink and privately enter their responses independent of study or school staff.

*Serology*. The same enrollment procedures are followed as described above. The blood sample collection is performed at the local health center.

#### Statistical methods

*Vaccination*. Vaccine coverage is the estimated percentage of female students who have received an HPV vaccination. Two types of vaccine coverage will be calculated in this study:

- Total vaccine coverage of Grade 8 schoolgirls: the total number of Grade 8 students that received at least one dose of the study HPV vaccine, or self-report previously receiving any HPV vaccine/ the total number of students in Grade 8 in the provinces
- Per protocol vaccine coverage of Grade 8 schoolgirls: the number of students in Grade 8 enrolled in the vaccination activity that completed the HPV vaccine regimen per protocol/ the number of eligible Grade 8 schoolgirls

Only students vaccinated per protocol will be eligible to enroll as vaccinated students in the Year 2 and Year 4 post-vaccination CSS. The AEFI as defined in the Thailand national guidelines will be summarized as reported after each vaccination and analyzed descriptively.

*HPV prevalence*. Cobas® results for vaccine-type HPV16 and 18 prevalence and Anyplex™ results for prevalence of HPV types 31, 33, 35, 39, 45, 51, 52, 56, 58, 59, 66, 68 (among Cobas-positive samples) will be reported by grade and by school type. The prevalence of HPV types 6, 11, 26, 40, 42, 43, 44, 53, 54, 61, 69, 70, 73, 82 will be calculated based on the Anyplex™ results of Cobas-positive samples, and of a subset of Cobas-negative samples, in order to estimate the frequency of these types in the Cobas-negative population. Because the sampling by school type is not proportional to enrollment, the HPV prevalence by province will be reported as a weighted prevalence of the two school types in the total population using Horvitz-Thompson adjustment [25].

*Vaccine Effectiveness (VE)*. The crude VE is defined as the percent reduction of HPV16 and/or 18 prevalence in a vaccinated group compared to an unvaccinated group and given by:

$$VE=100\times (1-\frac{p_{1}}{p_{2}}),$$

where *p*_1_ is the prevalence of HPV infection (HPV16 and/or 18) at Year 2 or Year 4 post vaccination and *p*_2_ is the prevalence of HPV infection (HPV16 and/or 18) of unvaccinated students in the Baseline CSS in the corresponding grade and province.

In addition, an adjusted VE will be computed by three methods as part of a sensitivity analysis and to account for the potential confounding effects of imbalance in age distribution and sexual activity between the non-contemporaneous groups. First, a simple adjusted VE will be calculated which adjusts only for the relative proportion of the 7 non-target HPV types (35, 39, 56, 58, 59, 66, 68) with no evidence of vaccine cross protection using the formula:

$$1-\frac{p_{2}/q_{2}}{p_{1}/q_{1}}.$$

Where *p*_1_ and *p*_2_ denote the weighted HPV 16 and/or 18 prevalence rates in the Baseline and Year 2 and Year 4 surveys, respectively. Similarly, *q*_1_ and *q*_2_ denote the weighted overall prevalence rates for the non-target genotypes in the Baseline and Year 2 and Year 4 surveys, respectively. The VE will also be adjusted using differences in the proportion of self-reported sexual activity from the SBQ.

Finally, a propensity score will be computed from a logistic regression that contained age and binary test result of each of 7 non-target HPV types as covariates. VE stratified by quintiles of propensity scores will be computed.

*Non-inferiority test*. The VE difference between the SD and 2D regimens (SD minus 2D) with the corresponding 95% confidence intervals (CI) will be calculated to test the VE of SD is non-inferior to VE of 2D with non-inferiority margin of 10% at Year 4.

*Herd protection*. This exploratory analysis will only be conducted if there is a sufficient number of unvaccinated students who enroll in the Year 2 and Year 4 surveys. The crude herd protection (HP) will be the percent reduction of HPV16 and/or 18 prevalence among unvaccinated students at Year 2 or Year 4 post vaccination as compared to the prevalence in the corresponding grade and province in the Baseline CSS. An adjusted HP will be calculated using the same methods as for adjusted VE.

*Serology*. The HPV type-specific antibody titers at Baseline, Year 2 and Year 4 post vaccination will be logarithmically transformed to better approximate normality and reported as a weighted GMT average with two-sided 95% CI by province.

CI for a single proportion will be defined using the Wilson method and a CI for a difference between independent proportions will be computed using the extension of the Wilson method and CI for a ratio of proportions will be derived using the delta method [26, 27]. All hypotheses testing will be carried out at a one-sided 0.025 significance level unless otherwise specified. Should any of the statistical methods proposed prove unsuitable during the final analysis, more appropriate methods will be used, and any changes documented in the study report, including the rational for use. Additional ad-hoc analyses may be conducted as deemed appropriate. All statistical analysis will be performed using SAS v9.4 and R.

#### Per protocol set analysis

The VE will be calculated using only subjects who met all protocol requirements, received the designated vaccine regimen in G8 and enrolled in a later CSS two and/or four years after vaccination. The unvaccinated Baseline CSS data set will be restricted to include only subjects who based on their date of birth would have been eligible to receive vaccine (<15 years of age) in G8 so that the vaccinated set and the unvaccinated comparator set have the same age distribution.

### Data and safety monitoring

Since the vaccine is already in use in the national immunization program and administered within the approved age range, the establishment of a Data and Safety Monitoring Board (DSMB) is not required. Because the schoolgirls in one province are offered a reduced dosing schedule, a futility assessment will be performed after the Year 2 survey with pre-determined criteria that, if met, would indicate no public health value to the SD regimen. If the futility criteria are met, a second dose of HPV vaccine will be offered to the vaccinated students in Udon Thani.

The futility criteria for considering the administration of a second dose of vaccine are:

- The estimated effectiveness of two doses after cross-sectional survey in Buri Ram must have a lower bound >50% AND
- The estimated effectiveness of single dose after Year 2 cross-sectional survey in Udon Thani must have an upper bound of <50%, AND
- Be inferior to 2D with a margin of >25% difference in vaccine effectiveness.

The sponsor has contracted independent local clinical monitors to ensure that the study is conducted according to protocol, standard operating procedures, the principles of GCP, and to verify that investigators are collecting and reporting quality data. During periods of field activities in the year of vaccination and Baseline CSS, and during the Year 2 and Year 4 CSS, the monitors are present at site to observe the informed consent process, including 100% review of ICFs and assent forms, and to verify adherence to study protocol. Data quality is further verified by monitors through direct source document verification against data in the electronic data capture system of at least 10% of randomly selected study participants.

### Confidentiality

All study-related information and data is being securely managed including use of physical security (locked rooms and cabinets) for source documents in Thailand, and secure servers for storage of digital data. The primary data to be used for analysis is located on a server at IVI, however a duplicate set of data is stored in Thailand. Only the PI and limited MOPH staff have access to personally identifiable information, and all data stored at IVI and Chulalongkorn University use only study ID number or a laboratory code number as an identifier. The serum samples, labeled with a laboratory code, will be sent outside the country for analysis at the US CDC. Both urine and serum samples will be stored for five years after study completion.

## Dissemination policy

Upon completion of each phase of the study (Baseline, Year 2, Year 4), an analysis will be conducted, and a study report produced. The report will be submitted to the competent authority and ethics committees according to national regulations and publication sought in peer-reviewed journals. In order to shorten the time between availability of data and public health decision-making, study reports will be made available to national (Thailand) and international (WHO) public health policy makers. We will report the study in accordance with the Consolidated Standards of Reporting Trials guidelines, with authorship based on the ICMJE recommendations.

## Supporting information

S1 Checklist (DOC)

S1 File ENG HPV study protocol V6.0_15DEC2021. (PDF)

S2 File Inclusivity questionnaire. (PDF)
