# Supplementary material for: A community intervention effectiveness study of single dose or two doses of bivalent HPV vaccine (CERVARIX®) in female school students in Thailand
Source: PLoS One. 2022 Apr 28;17(4):e0267294. doi: 10.1371/journal.pone.0267294 (PMC9049519; doi:10.1371/journal.pone.0267294)
Supplement: S1 Checklist — (DOC) [file pone.0267294.s001.doc]

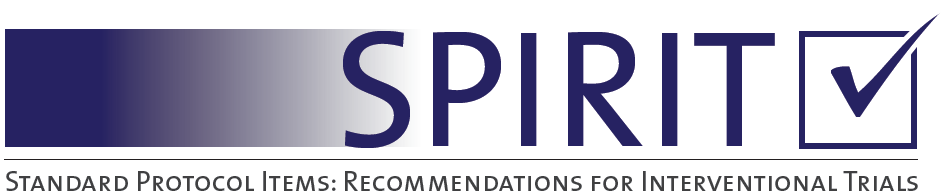


SPIRIT 2013 Checklist: Recommended items to address in a clinical trial protocol and related documents*

| Section/item | ItemNo | Description |
| --- | --- | --- |
| **Administrative information** | | |
| Title | 1 | A community intervention effectiveness study of single dose or two doses of bivalent HPV vaccine (CERVARIX®) in female school students in Thailand |
| Trial registration | 2a | The trial is registered at clinicaltrials.gov with the registry name **“**Effectiveness of Single Dose or Two Doses of Bivalent HPV Vaccine in Thailand (IVIHPV1)**”** and identifier NCT03747770 |
|  | 2b | **N/A** |
| Protocol version | 3 | 2018-005 V6.0 on 15 Dec 2021 |
| Funding | 4 | This work was supported by the Bill & Melinda Gates Foundation (<https://www.gatesfoundation.org/>) through a grant [OPP1188728]. In addition, the Governments of Korea, Sweden, India and Finland provide core/unrestricted support to the International Vaccine Institute. |
| Roles and responsibilities | 5a | Authorship List:  Suchada Jiamsiri^1^, Chulwoo Rhee^2^, Hyeon Seon Anh^2^, Nimesh Poudyal^2^, Hyeong-Won Seo^2^, Worrawan Klinsupa^1^, Pornjarim Nilyanimit^3^, Nakorn Premsri^4^, Chawetsan Namwat^5^, Sompong Vonpunsawad^3^, Yun Chon^2^, Sunju Park^2^, Deok-Ryun Kim^2^, Elizabeth R. Unger^6^, Lauri Markowitz^7^, Yong Poovorawan^3^, Supachai Rerks-Ngarm^1^, Jean Louis Excler^2^, Julia Lynch^2^  1. Division of Vaccine Preventable Diseases, Department of Disease Control, Ministry of Public Health, Nonthaburi, Thailand  2. International Vaccine Institute, Seoul, Republic of Korea  3. Center of Excellence in Clinical Virology, Department of Pediatrics, Faculty of Medicine, Chulalongkorn University, Bangkok, Thailand  4. National Vaccine Institute, Ministry of Public Health, Nonthaburi, Thailand  5. Bureau of Epidemiology, Department of Disease Control, Ministry of Public Health, Nonthaburi, Thailand  6. Chronic Viral Diseases Branch, Division of High-Consequence Pathogens and Pathology, National Center for Emerging and Zoonotic Infectious Diseases, US Centers for Disease Control and Prevention, Atlanta GA, USA  7. Viral Vaccine Preventable Diseases Branch, Division of Viral Diseases, National Center for Immunization and Respiratory Diseases, US Centers for Disease Control and Prevention, Atlanta GA, USA  All authors contributed to the development of study design and analysis plans. SJ, CR, NP, SV, DK, EU, LM, YP, SP, SR, JLE, JL contributed in manuscript writing and editing. All authors read and approved the final manuscript. |
|  | 5b | Sponsor: International Vaccine Institute, SNU Research Park, 1 Gwanak-ro, Gwanak-gu, Seoul, Korea (+82-2-881-1149), Julia.lynch@ivi.int |
|  | 5c | The funder was not involved in decisions regarding study design, data collection, management, analysis, decision to publish or preparation of the manuscript. Under the grant conditions of the funder, a Creative Commons Attribution 4.0 Generic License has already been assigned to the Author Accepted Manuscript version that might arise from this submission. The design, management, analysis, and reporting of the study are entirely independent of the vaccine manufacturer. |
|  | 5d | **N/A** |
| Introduction |  |  |
| Background and rationale | 6a | Human Papillomavirus (HPV) infection of the reproductive tract is common, principally spread through sexual activity. The majority of HPV infections are asymptomatic and resolve spontaneously. However, persistent infection with specific HPV types (most frequently HPV 16 and 18) may lead to precancerous lesions which if untreated may progress to cervical and some other cancers.[1] In 2020 it was estimated that 604,000 new cases of cervical cancers occurred in women with over 340,000 cervical cancer related deaths.[2] As a result of limited availability of screening and treatment, 85% of the deaths occur in resource-limited settings.  All available HPV vaccines are virus-like particle (VLP) vaccines and have been shown to have high efficacy in randomized controlled trials.[3] CERVARIX® (GlaxoSmithKline) and Gardasil® (Merck & Co) received WHO prequalification in 2009, and Gardasil®9, in 2018. Cecolin® (Xiamen Innovax Biotech Co) was recently licensed in China and currently under review for World Health Organization (WHO) prequalification. HPV vaccines are ideally administered prior to the initiation of sexual activity. Although originally licensed based on a three-dose schedule (0, 1-2, 6 months) in young adult women, in 2014 the WHO guidelines recommended a two-dose regimen (0, 6 months) for girls aged 9-14 years based on the non-inferiority of antibody responses of the two-dose schedule in that age group as compared to the three-dose schedule efficacious among women.[4, 5]  Despite reductions in the cost of these vaccines through the Global Alliance for Vaccine and Immunization (GAVI) or tiered-pricing, the uptake of HPV vaccines into national programs has been slow particularly in low- and middle-income countries (LMIC).[6] Barriers to uptake include cost (vaccine and delivery cost), and programmatic challenges (novel age of target group, competing new vaccine introduction priorities) and vaccine availability. Thailand introduced HPV vaccine into a nationwide school-based program in 2017, targeting girls in Grade 5. However, catch up immunization programs among school girls older than Grade 5 was stalled due to limited funding.  Post-hoc analyses of randomized trials have found high vaccine effectiveness following a single dose (SD) of bivalent or quadrivalent vaccine.[[7-9](#_ENREF_37)] However, the interpretation of these analyses is limited by several factors including women with incomplete vaccination schedules not randomized by number of doses, small sample size, and low number of incident or persistent infections.  SD HPV vaccination would enable twice as many female students to be covered with the same amount of vaccine supply, facilitate catch-up vaccination campaigns, and alleviate the overall economic cost of vaccine supply and delivery, while averting treatment costs of disease. WHO acknowledges that more data are needed to issue such a recommendation.[6]  In order to provide additional data on the potential impact of SD HPV vaccination in a real-world setting, we are conducting an effectiveness study of the SD and the standard two-dose (2D) regimens in two provinces in Thailand. Vaccination is administered in schools, mirroring the already implemented national Grade 5 HPV immunization program, but targeting Grade 8 females outside the grade eligibility |
|  |  | |
| Objectives | 7 | Primary objectives  Demonstrate effectiveness of SD HPV vaccination by a reduction in vaccine-type HPV prevalence (HPV16 and/or 18) at two and four years post vaccination compared to the prevalence among unvaccinated same grade female students collected in a baseline survey.  Demonstrate effectiveness of SD and 2D HPV vaccine regimens are similar by comparing reductions in vaccine HPV-type prevalence at four years post vaccination compared with the baseline prevalence in the two provinces.    Secondary objectives   - Estimate prevalence of HPV infection in female students in high school Grades 10 and 12 (G10, G12) and vocational school years 1 and 3 (V1, V3) - Estimate the distribution of HPV types detected - Assess HPV type-specific antibody response pre- and post-vaccination in a subset of participants   Exploratory objectives   - Assess comparability of sexual behavior between the two provinces among female students in Grades 8, 10 and 12 (G8, G10, G12) and vocational school years 1 and 3 (V1, V3) - Assess comparability of risk for HPV infection between G10/V1 and G12/V3 female students at baseline and at years 2 and 4 post vaccination - Assess possible herd protection conferred by the HPV vaccination in unvaccinated female students |
| Trial design | 8 | This is an observational community effectiveness study of SD or 2D of CERVARIX® (containing HPV16 and HPV18) administered in a voluntary school-based program to Grade 8 female students less than age 15 years in two provinces of Thailand, Udon Thani and Buri Ram. The overall study is divided into four independent parts:   - Part A – Grade 8 vaccination and sexual behavior questionnaire (SBQ) - Part B – Baseline cross-sectional survey (CSS) of HPV prevalence based on self-collected urine samples and SBQ (G10/V1 and G12/V3 ) - Part C – CSS of HPV prevalence based on self-collected urine samples and SBQ two years after vaccination (G10/V1) - Part D – CSS of HPV prevalence based on self-collected urine samples and SBQ four years after vaccination (G12/V3)   Vaccine effectiveness will be assessed as the reduction in vaccine HPV-type infection measured in a CSS two and four years after vaccination, compared to the Baseline CSS. A subset of students in each province (N=200) will be invited to participate in a sub-study to evaluate immune response to vaccination. |
| Methods: Participants, interventions, and outcomes | | |
| Study setting | 9 | This study will be conducted in two provinces in North-East Thailand, Udon Thani and Buri Ram, selected by the Ministry of Public Health based on the similarity of criteria such as size of the female student population, socio-economic parameters, comparability of self-reported sexual activity in a national survey, logistics feasibility, and high degree of acceptance of the Grade 5 HPV vaccine program by local authorities and communities. Each province is further sub-divided into Districts, each with a District Hospital and subordinate Health Centers (Table 2). This public health infrastructure is used to deliver school-based vaccinations including the Grade 5 HPV vaccination program and Grade 6 diphtheria and tetanus boosters. This infrastructure and experience will be leveraged to conduct the Grade 8 HPV vaccination campaign for this study over a three-month period. After Grade 9, students are tracked into either regular high school to complete Grades 10-12 (G10-G12) or vocational schools to complete grades vocational year 1-3 (V1-V3). Concurrent with the vaccination campaign, MOPH and school-based staff will conduct the Baseline CSS at the high schools and vocational schools. By random selection, the 2D and SD regimens were allocated to Buri Ram and Udon Thani, respectively. |
| Eligibility criteria | 10 | All schools with the targeted grade students in each Province are invited to participate. Student Inclusion and Exclusion criteria are below.   | Study Component | **Inclusion criteria** | **Exclusion criteria** | | --- | --- | --- | | **Grade 8 Vaccination** | - Female students with identification card - Less than 15 years of age - Parent or guardian consent for vaccination and blood collection as applicable - Participant assent for vaccination, SBQ, and blood collection as applicable | - Students who already received HPV vaccination - Reported pregnancy - Any student who has a preexisting known medical condition or diagnosed psychological illness which in the opinion of the Principal Investigator or designee may be detrimental to her wellbeing | | **Baseline CSS** | - Female students with identification card - Participant assent for SBQ and urine collection | - Any student who has a preexisting known medical condition or diagnosed psychological illness which in the opinion of the Principal Investigator or designee may be detrimental to her well-being | | **Year 2 and Year 4 post-vaccination CSS** | - Female students with identification card - Parent or guardian consent for blood collection as applicable - Participant assent for SBQ, urine, and blood collection from those vaccinated at Grade 8 as applicable | - Any student who has a preexisting known medical condition or diagnosed psychological illness which in the opinion of the Principal Investigator or designee may be detrimental to her wellbeing | |
| Interventions | 11a | CERVARIX® is a bivalent HPV vaccine manufactured by GlaxoSmithKline plc. and presented as a suspension containing purified viral L1 protein for HPV types 16 and 18 and administered by intramuscular injection. It is produced using a baculovirus expression system. Each 0.5 mL dose of the bivalent vaccine contains 20 μg of HPV16 L1 protein and 20 μg of HPV18 L1 protein formulated with AS04 (containing 500 μg of aluminum hydroxide and 50 μg of 3-O-desacyl-4-monophosphoryl lipid A. This vaccine is indicated for use in females and males from the age of 9 years for the prevention of premalignant anogenital lesions affecting the cervix, vulva, vagina and anus, and cervical and anal cancers causally related to specific HPV types.[10, 11]  Eligible grade 8 students in Udon Thani received a single-dose of vaccine and in Buri Ram eligible students received two doses with an approximate 6 month interval as per national guidelines. |
|  | 11b | Criteria for discontinuing or modifying allocated interventions for a given trial participant (eg, drug dose change in response to harms, participant request, or improving/worsening disease)- **N/A** |
|  | 11c | Strategies to improve adherence to intervention protocols, and any procedures for monitoring adherence (eg, drug tablet return, laboratory tests)-**N/A** |
|  | 11d | Relevant concomitant care and interventions that are permitted or prohibited during the trial-**N/A** |
| Outcomes | 12 | Primary endpoint  • HPV 16 and HPV 18 DNA prevalence as measured in urine by DNA PCR at Year 2 and Year 4 post vaccination compared to unvaccinated female students  Secondary endpoints  • HPV 16 and 18 infection prevalence in Grades 10/year 1 and Grade 12/year 3 female students at baseline survey, Year 2 and Year 4 surveys post vaccination  • Distribution of HPV genotypes in HPV infections detected during the surveys.  • HPV type-specific antibody response in a subset of participants prior to vaccination and at Year 2 and Year 4 post vaccination to ensure and document that the vaccine used in this study is immunogenic. Exploratory Endpoints  • Sexual behaviour including sexual debut of female students from Grades 8, 10/year 1 and 12/year 3.  • Reduction in HPV prevalence among un-vaccinated students- herd protection |
| Participant timeline | 13 | Time schedule of enrolment, interventions (including any run-ins and washouts), assessments, and visits for participants. A schematic diagram is highly recommended (see Figure)  The activities after enrolment in each component of the study are completed in 1 day during the time window indicated in the table and there will be no follow-up visits.   | Study Component | Timeline | Sample Size | Intervention/Activity | | --- | --- | --- | --- | | Part A: Vaccination | 2018 Dec-2019 Feb | All eligible 8,000~9,000* schoolgirls (<15 years old) in Grade 8 per province  N=1,500 for SBQ  N=200 (pre-vaccine serology) | Udon Thani: single-dose HPV vaccine regimen  Buri Ram: two-dose HPV vaccine regimen 6 months apart | | Part B: Baseline CSS | 2018 Dec-2019 Feb | G10/V1: N=2,600 schoolgirls per province  G12/V3: N=2,000 schoolgirls per province | SBQ and HPV urine prevalence | | Part C: Year 2 CSS | 2020 Dec-2021 Feb | G10/V1: N=2,600 schoolgirls per province  N=200 (serology) | SBQ and HPV urine prevalence | | Part D: Year 4 CSS | 2022 Dec-2023 Feb | G12/V3: N=2,000 schoolgirls per province  N=200 (serology) | SBQ and HPV urine prevalence | |
| Sample size | 14 | Estimated number of participants needed to achieve study objectives and how it was determined, including clinical and statistical assumptions supporting any sample size calculations- See figure above |
| Recruitment | 15 | All aspects of recruitment and enrolment will be conducted according to ICH E6(R2) GCP guidelines. For each of the activities described below, eligibility is assessed according to the inclusion and exclusion criteria. Parents or guardian are informed about the activity in their own language by school staff and MOPH study staff and offered an opportunity to ask questions.  Vaccination  Open label vaccination with either SD (Udon Thani) or 2D regimen (Buri Ram) are offered to all Grade 8 students younger than age 15 years between December 2018-February 2019 on a voluntary basis. A subset of 1500 students in each province are selected by a proportional and systematic sampling method and invited to complete the SBQ. For Grade 8 students less than 15 years of age, both a parental consent and student assent form is completed prior to enrolment for vaccination, and a separate assent form is obtained for the SBQ.  Cross-sectional surveys  Cross-sectional surveys (CSS) are intended to sample a representative subset of students in the province, and all schools willing to participate are included in the surveys. As sexual activity is expected to be higher among vocational students (based on existing national survey data), and the overall prevalence of vaccine-type HPV infections is expected to be low, vocational students will be over sampled to achieve ~50% of all enrolled subjects. Based on the overall province and school-type sample size, school-specific target enrolments will be calculated to be proportionate to their grade enrolments. At each school, students will be selected to participate in the CSS based on a systematic sampling from an eligibility list. When selected students are either not eligible because of exclusion criteria or decide not to participate in the study, additional students are selected by the field staff using the same method until the school target enrolment is reached. If the school target enrolment cannot be met, study staff can over enrol at another school of the same type in order to meet the province target enrolment. In order to ensure student confidentiality, only student assent is required for enrolment in the CSS and SBQ. In the Year 2 and Year 4 CSS, the student vaccination status is verified and documented at the time of enrolment.  Serology  For practical purposes, a non-random subset of 200 Grade 8 students in each province consenting to vaccination and living in close proximity of a District Hospital will be invited to participate in this sub-study. A blood sample will be taken prior to vaccination and at the time of the Year 2 and Year 4 CSS. Eligibility criteria are re-assessed by District Health staff at the time of blood sample collection. Both parental consent and student assent forms are completed for each blood draw prior to vaccination, and at G10/V1 and G12/V3. |
| **Methods: Assignment of interventions (for controlled trials)** | | |
| Allocation: |  | By random selection, the two-dose and single-dose regimens were allocated to Buri Ram and Udon Thani, respectively. |
| Sequence generation | 16a | Method of generating the allocation sequence (eg, computer-generated random numbers), and list of any factors for stratification. To reduce predictability of a random sequence, details of any planned restriction (eg, blocking) should be provided in a separate document that is unavailable to those who enrol participants or assign interventions-**N/A** |
| Allocation concealment mechanism | 16b | Mechanism of implementing the allocation sequence (eg, central telephone; sequentially numbered, opaque, sealed envelopes), describing any steps to conceal the sequence until interventions are assigned-**N/A** |
| Implementation | 16c | Who will generate the allocation sequence, who will enrol participants, and who will assign participants to interventions-**N/A** |
| Blinding (masking) | 17a | Who will be blinded after assignment to interventions (eg, trial participants, care providers, outcome assessors, data analysts), and how- **N/A-**Open Label study |
|  | 17b | If blinded, circumstances under which unblinding is permissible, and procedure for revealing a participant’s allocated intervention during the trial-**N/A** |
| **Methods: Data collection, management, and analysis** | | |
| Data collection methods | 18a | Urine samples are self-collected by the students at school using a commercially available urine collection device, Colli-Pee® (Novosanis, Belgium; FV-5000 series N00176) pre-labeled with coded subject ID. The device contains 7 ml of UCM preservative and collects up to 20 ml of the initial void of urine. This collection method has been successfully used in Bhutan and Rwanda for HPV prevalence assessments, and demonstrated a high degree of comparability to physician-collected cervical swabs in a pilot study in Thailand.[14-18]. Urine samples are then stored at 2-8°C within 30 minutes and transported via monitored cold chain to Chulalongkorn University lab in Bangkok for further processing within 48 hours of reception. A 10 ml aliquot of urine is centrifuged, 9 ml of supernatant removed and the cell pellet re-suspended in remaining one ml and frozen at -20°C until HPV DNA testing. A second aliquot of urine is frozen at -20°C for further testing, if required.  Cobas® (Roche), a high throughput, qualitative assay system will be used for detection of HPV16 and 18 and 12 other HPV types (31, 33, 35, 39, 45, 51, 52, 56, 58, 59, 66, 68).[19-21] Residual extracts from all Cobas-positive samples and equal numbers of Cobas-negative samples will be frozen -20^o^ C and subsequently assayed with Anyplex™ (Seegene) to identify 28 HPV types.[22, 23] Cobas® and Anyplex™ testing will be initiated in parallel with field collection activities and continue until all samples from the activity year are tested. Test results will be uploaded to the electronic data capture (EDC) system by Chulalongkorn lab staff.  Blood (5 ml) will be collected via antecubital venipuncture by trained phlebotomist into serum tubes and allowed to clot at room temperature. Sera will be aliquoted into 1 ml cryovials and stored at -70^o^C and shipped on dry ice to the US CDC laboratory for testing using multiplex direct IgG ELISA against L1/L2 HPV virus-like particles on the Meso Scale Discovery platform with chemiluminescent detection as described with minor modifications.[24]  The SBQ is a subset of 8 questions derived from the Thailand Ministry of Health National Survey on sexual behaviour which is administered to school students periodically.  A limited amount of data is collected on a field source document including name, national ID, date of birth and date of vaccination. Each student agreeing to vaccination (or any other study activity) is assigned a pre-printed subject number in numerical and bar-code format which codes for province, school, a unique number, grade, and activity type (e.g., vaccination, CSS, serology). The pre-printed barcodes are affixed to the consent and assent documents and the source document, which are maintained only by the MOPH staff. Only the complete subject number, date of birth, date of consent or assent and date of vaccination are entered into the EDC system by study staff.  The SBQ is a web-based, self-administered questionnaire. Using their own cell phone or via a tablet or phone provided by study staff, students scan their assigned subject number barcode at a weblink and privately enter their responses independent of study or school staff. |
|  | 18b | Plans to promote participant retention and complete follow-up, including list of any outcome data to be collected for participants who discontinue or deviate from intervention protocols-**N/A** |
| Data management | 19 | Plans for data entry, coding, security, and storage, including any related processes to promote data quality (eg, double data entry; range checks for data values). Reference to where details of data management procedures can be found, if not in the protocol  An electronic web-based system for data management using paper and paper-less will be developed by IVI for the collection of participant-specific information. Lab data will be electronically transferred to the IVI main database system for data quality control. This data management (DM) system will include computer system functionality of entry, editing, browsing, downloading and uploading, providing error reports, exception lists and summary reports for each activity. In addition, audit trail of DM system would record all sequential changes made in the database. Data entry programs will incorporate identification of the range, consistency and duplication checks. Data quality is further verified by sponsor contracted monitors through direct source document verification against data in the electronic data capture system of at least 10% of randomly selected study participants. The database will be located and managed at the IVI Biostatistics and Data Management Department. Data security for this data management system will be reinforced by automatic computer virus scanning at start-up of each data entry and data management session, and password protection for accessing data and data management software. Regarding confidentiality of data, the access to database will be controlled by user ID and password depending on their role and responsibility of study team members and the personal identification information of participants will not be stored in IVI server. In addition, backup files generated by the data management system will be kept in a secure place. Data entry and cleaning will be conducted at the sites. Final data cleaning, data locking and data analysis will be performed at the IVI. |
| Statistical methods | 20a | Statistical methods for analysing primary and secondary outcomes. Reference to where other details of the statistical analysis plan can be found, if not in the protocol Vaccine coverage is the estimated percentage of female students who have received an HPV vaccination. Two types of vaccine coverage will be calculated in this study:   - Total vaccine coverage of Grade 8 schoolgirls: the total number of Grade 8 students that received at least one dose of the study HPV vaccine, or self-report previously receiving any HPV vaccine/ the total number of students in Grade 8 in the provinces - Per protocol vaccine coverage of Grade 8 schoolgirls: the number of students in Grade 8 enrolled in the vaccination activity that completed the HPV vaccine regimen per protocol/ the number of eligible Grade 8 schoolgirls   Only students vaccinated per protocol will be eligible to enroll as vaccinated students in the Year 2 and Year 4 post-vaccination CSS. The AEFI as defined in the Thailand national guidelines will be summarized as reported after each vaccination and analyzed descriptively.  HPV Prevalence  Cobas® results for vaccine-type HPV16 and 18 prevalence and Anyplex™ results for prevalence of HPV types 31, 33, 35, 39, 45, 51, 52, 56, 58, 59, 66, 68 (among Cobas-positive samples) will be reported by grade and by school type. The prevalence of HPV types 6, 11, 26, 40, 42, 43, 44, 53, 54, 61, 69, 70, 73, 82 will be calculated based on the Anyplex™ results of Cobas-positive samples, and of a subset of Cobas-negative samples, in order to estimate the frequency of these types in the Cobas-negative population. Because the sampling by school type is not proportional to enrollment, the HPV prevalence by province will be reported as a weighted prevalence of the two school types in the total population using Horvitz-Thompson adjustment [25].  Vaccine Effectiveness (VE)  The crude VE is defined as the percent reduction of HPV16 and/or 18 prevalence in a vaccinated group compared to an unvaccinated group and given by:  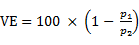,where 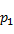 is the prevalence of HPV infection (HPV16 and/or 18) at Year 2 or Year 4 post vaccination and 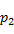 is the prevalence of HPV infection (HPV16 and/or 18) of unvaccinated students in the Baseline CSS in the corresponding grade and province.  *Non-inferiority test*  The VE difference between the SD and 2D regimens (SD minus 2D) with the corresponding 95% confidence intervals (CI) will be calculated to test the VE of SD is non-inferior to VE of 2D with non-inferiority margin of 10% at Year 4.  *Herd Protection*  The crude herd protection (HP) will be the percent reduction of HPV16 and/or 18 prevalence among unvaccinated students at Year 2 or Year 4 post vaccination as compared to the prevalence in the corresponding grade and province in the Baseline CSS. An adjusted HP will be calculated using the same methods as for adjusted VE.  All statistical analysis will be performed using SAS v9.4 and R. |
|  | 20b | Methods for any additional analyses (eg, subgroup and adjusted analyses)  In addition, an adjusted VE will be computed by three methods as part of a sensitivity analysis and to account for the potential confounding effects of imbalance in age distribution and sexual activity between the non-contemporaneous groups. First, a simple adjusted VE will be calculated which adjusts only for the relative proportion of the 7 non-target HPV types (35, 39, 56, 58, 59, 66, 68) with no evidence of vaccine cross protection using the formula:  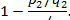where 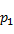 and 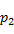 denote the weighted HPV 16 and/or 18 prevalence rates in the Baseline and Year 2 and Year 4 surveys, respectively. Similarly, 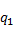 and 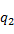 denote the weighted overall prevalence rates for the non-target genotypes in the Baseline and Year 2 and Year 4 surveys, respectively. The VE will also be adjusted using differences in the proportion of self-reported sexual activity from the SBQ.  Finally, a propensity score will be computed from a logistic regression that contained age and binary test result of each of 7 non-target HPV types as covariates. VE stratified by quintiles of propensity scores will be computed. |
|  | 20c | Definition of analysis population relating to protocol non-adherence (eg, as randomised analysis), and any statistical methods to handle missing data (eg, multiple imputation)  The VE will be calculated using only subjects who met all protocol requirements, received the designated vaccine regimen in G8 and enrolled in a later CSS two and/or four years after vaccination. The unvaccinated Baseline CSS data set will be restricted to include only subjects who based on their date of birth would have been eligible to receive vaccine (<15 years of age) in G8 so that the vaccinated set and the unvaccinated comparator set have the same age distribution. |
| **Methods: Monitoring** | | |
| Data monitoring | 21a | Composition of data monitoring committee (DMC); summary of its role and reporting structure; statement of whether it is independent from the sponsor and competing interests; and reference to where further details about its charter can be found, if not in the protocol. Alternatively, an explanation of why a DMC is not needed-  Since the vaccine is already in use in the national immunization program and administered within the approved age range, the establishment of a Data and Safety Monitoring Board (DSMB) is not required. |
|  | 21b | Description of any interim analyses and stopping guidelines, including who will have access to these interim results and make the final decision to terminate the trial  Because the schoolgirls in one province are offered a reduced dosing schedule, a futility assessment will be performed after the Year 2 survey with pre-determined criteria that, if met, would indicate no public health value to the SD regimen. If the futility criteria are met, a second dose of HPV vaccine will be offered to the vaccinated students in Udon Thani.  The futility criteria for considering the administration of a second dose of vaccine are:   - The estimated effectiveness of two doses after cross-sectional survey in Buri Ram must have a lower bound >50% AND - The estimated effectiveness of single dose after Year 2 cross-sectional survey in Udon Thani must have an upper bound of <50%, AND - Be inferior to 2D with a margin of >25% difference in vaccine effectiveness.   The results of the futility assessment will be shared with the MOPH and the relevant IRBs including the MOPH EC. |
| Harms | 22 | Plans for collecting, assessing, reporting, and managing solicited and spontaneously reported adverse events and other unintended effects of trial interventions or trial conduct  Post vaccination monitoring of the Grade 8 subjects is conducted as in other school-based programs and adverse events following immunization (AEFI) recorded through the national surveillance system and reported as per national guidelines for this licensed product. The investigators will exercise due diligence in ascertaining, and accurately recording all reportable AEFI that vaccinated study participants may experience. Reported AEFI are linked to the study ID of the students. AEFI may also be reported to the MOPH EC as per local regulations. |
| Auditing | 23 | Frequency and procedures for auditing trial conduct, if any, and whether the process will be independent from investigators and the sponsor  The sponsor has contracted independent local clinical monitors to ensure that the study is conducted according to protocol, standard operating procedures, the principles of GCP, and to verify that investigators are collecting and reporting quality data. During periods of field activities in the year of vaccination and Baseline CSS, and during the Year 2 and Year 4 CSS, the monitors are present at site to observe the informed consent process, including 100% review of ICFs and assent forms, and to verify adherence to study protocol.  IVI QA Department performs qualifications of all vendors, and audits as required according to established SOPs. |
| Ethics and dissemination | | |
| Research ethics approval | 24 | Plans for seeking research ethics committee/institutional review board (REC/IRB) approval  The protocol was approved by the IVI IRB (IVI HPV1 2018-005 V6.0) on 15 Dec 2021, the Thailand Ministry of Health EC (Ref.no.25/2561, V6.0) on 12 Jan 2022, and the Chulalongkorn University IRB (IRB No. 495/61 V5.0) on 8 Sep 2020. The US CDC IRB deferred to the IVI IRB on 7 May 2019. This paper is based on the protocol version Version 6.0 dated 15 Dec 2021. |
| Protocol amendments | 25 | Plans for communicating important protocol modifications (eg, changes to eligibility criteria, outcomes, analyses) to relevant parties (eg, investigators, REC/IRBs, trial participants, trial registries, journals, regulators)  Any amendment of the approved protocol shall be submitted for review and approval by the competent authorities in Thailand and at IVI. |
| Consent or assent | 26a | Who will obtain informed consent or assent from potential trial participants or authorised surrogates, and how (see Item 32)  Informed consent is obtained by trained MOPH study staff. |
|  | 26b | Additional consent provisions for collection and use of participant data and biological specimens in ancillary studies, if applicable-**N/A** |
| Confidentiality | 27 | How personal information about potential and enrolled participants will be collected, shared, and maintained in order to protect confidentiality before, during, and after the trial  All study-related information and data is being securely managed including use of physical security (locked rooms and cabinets) for source documents in Thailand, and secure servers for storage of digital data. The primary data to be used for analysis is located on a server at IVI, however a duplicate set of data is stored in Thailand. Only the PI and limited MOPH staff have access to personally identifiable information, and all data stored at IVI and Chulalongkorn University use only study ID number or a laboratory code number as an identifier. |
| Declaration of interests | 28 | Financial and other competing interests for principal investigators for the overall trial and each study site  There are no declared conflicts of interest. |
| Access to data | 29 | Statement of who will have access to the final trial dataset, and disclosure of contractual agreements that limit such access for investigators  Only authorized individuals on the study team, or monitors have access to data. The study principal investigator, and the co-investigators will have access to the final dataset for analysis. |
| Ancillary and post-trial care | 30 | Provisions, if any, for ancillary and post-trial care, and for compensation to those who suffer harm from trial participation.  No ancillary or post-study care is provided as the intervention is a licensed vaccine already delivered as part of the national program to healthy individuals. However, in case of harm from the study, the sponsor has secured clinical trial insurance for subjects. |
| Dissemination policy | 31a | Plans for investigators and sponsor to communicate trial results to participants, healthcare professionals, the public, and other relevant groups (eg, via publication, reporting in results databases, or other data sharing arrangements), including any publication restrictions  Upon completion of each phase of the study (Baseline, Year 2, Year 4), an analysis will be conducted, and a study report produced. The report will be submitted to the competent authority and ethics committees according to national regulations and publication sought in peer-reviewed journals. In order to shorten the time between availability of data and public health decision-making, study reports will be made available to national (Thailand) and international (WHO) public health policy makers. We will report the study in accordance with the Consolidated Standards of Reporting Trials guidelines. |
|  | 31b | Authorship eligibility guidelines and any intended use of professional writers  Authorship will be based on the ICMJE recommendations. There are no plans to use medical writers. |
|  | 31c | Plans, if any, for granting public access to the full protocol, participant-level dataset, and statistical code  Datasets including code will be made available on an open access data platform after study completion. |
| Appendices |  |  |
| Informed consent materials | 32 | Model consent form and other related documentation given to participants and authorised surrogates |
| Biological specimens | 33 | Plans for collection, laboratory evaluation, and storage of biological specimens for genetic or molecular analysis in the current trial and for future use in ancillary studies, if applicable- **N/A** |

*It is strongly recommended that this checklist be read in conjunction with the SPIRIT 2013 Explanation & Elaboration for important clarification on the items. Amendments to the protocol should be tracked and dated. The SPIRIT checklist is copyrighted by the SPIRIT Group under the Creative Commons “[Attribution-NonCommercial-NoDerivs 3.0 Unported](http://www.creativecommons.org/licenses/by-nc-nd/3.0/)” license.
