# Supplementary material for: A community intervention effectiveness study of single dose or two doses of bivalent HPV vaccine (CERVARIX®) in female school students in Thailand
Source: PLoS One. 2022 Apr 28;17(4):e0267294. doi: 10.1371/journal.pone.0267294 (PMC9049519; doi:10.1371/journal.pone.0267294)
Supplement: S1 File — (PDF) [file pone.0267294.s002.pdf]

A community intervention effectiveness study of single dose or two doses of bivalent HPV vaccine (CERVARIX) in female school students in Thailand

**Protocol Number**	IVI HPV1
**Principal Investigator**	Dr. Suchada Jiamsiri<br>Division of AIDS and STIs<br>Department of Disease Control<br>Ministry of Public Health<br>Nonthaburi, Thailand<br>Tel: +66 81 810 5664<br>*sjiamsiri@outlook.com*
**Sponsor**	International Vaccine Institute (IVI)<br>SNU Research Park, 1 Gwanak-ro,<br>Gwanak-gu, Seoul, 151-742<br>Republic of Korea<br>T: +82 2 881 1149<br>M: +82 2 10 7278 7221<br>*Julia.lynch@ivi.int*
**Funding Agency**	Bill & Melinda Gates Foundation
**Version Number, Date**	Version 6.0 15DEC2021

KEY ROLES

Principal Investigator (MOPH/DDC)
Dr. Suchada Jiamsiri
Division of AIDS and STIs
Department of Disease Control
Ministry of Public Health
Nonthaburi, Thailand
Tel: +66 81 810 5664
sjiamsiri@outlook.com

BMGF Grant Principal Investigator (IVI)
Dr. Julia Lynch
International Vaccine Institute
SNU Research Park, 1 Gwanak-ro,
Gwanak-gu, Seoul, 151-742
Republic of Korea
T: +82 2 881 1149
M: +82 2 10 7278 7221
julia.lynch@ivi.int

DDC Grant Principal Investigator
Dr. Patummal Silaporn
Deputy Director,
Division of General Communicable Diseases
Ministry of Public Health
Nonthaburi, Thailand
Tel: +66 02 590 3338
Email: *patummal@hotmail.com*

IVI Principal Investigator
Dr. Chulwoo Rhee
International Vaccine Institute
SNU Research Park, 1 Gwanak-ro,
Gwanak-gu, Seoul, 151-742
Republic of Korea
Tel: +82 10 6856 3878
Daniel.Rhee@ivi.int

Site Manager
Worrawan Klinsupa
Division of AIDS and STIs
Department of Disease Control
Ministry of Public Health
Nonthaburi, Thailand
Tel: +66 81 554 1048
kworrawan@hotmail.com

Research Laboratory Investigators

Professor Yong Poovorawan
Center of Excellence in Clinical Virology
Department of Pediatrics
Faculty of Medicine, Chulalongkorn University
Bangkok, 10330 Thailand
Tel: +66 2 256 4909
Fax: +66 2 256 4929
yong.p@chula.ac.th

Dr. Sompong Vongpunswad
Center of Excellence in Clinical Virology
Department of Pediatrics
Faculty of Medicine, Chulalongkorn University
Bangkok, 10330 Thailand
T: +66 2 256 4909
M: +66 90 998 0397
sompong.vo@chula.ac.th

Pornjarim Nilyanimit
Center of Excellence in Clinical Virology
Department of Pediatrics
Faculty of Medicine, Chulalongkorn University
Bangkok, 10330 Thailand
Tel: +66 61 564 5256
mim_bhni@hotmail.com

Elizabeth Unger
Chief of Chronic Viral Diseases Branch
Division of High-Consequence Pathogens and Pathology
National Center for Emerging and Zoonotic Infectious Diseases
US Centers for Disease Control and Prevention
1600 Clifton Road, Atlanta GA 30329, USA
Tel: +1 404 639 8359
eru0@cdc.gov

Advisers

Dr. Lauri Markowitz
Team Lead, HPV
Viral Vaccine Preventable Diseases Branch
Division of Viral Diseases
Associate Director of Science for HPV
National Center for Immunization and Respiratory Diseases
US Centers for Disease Control and Prevention
1600 Clifton Road, Atlanta GA 30329, USA
Tel: +1 404 639 8359
lem2@cdc.gov

Dr. Anh Wartel
International Vaccine Institute
SNU Research Park, 1 Gwanak-ro,
Gwanak-gu, Seoul, 151-742
Republic of Korea
Tel: +82 10 5056 2512
anh.wartel@ivi.int

Study Oversight Monitor

Dr. Supachai Rerks-Ngarm
Department of Disease Control
Ministry of Public Health, Nonthaburi, Thailand
Tel: +66 81 821 7553
supachai.moph@gmail.com

Epidemiology

Dr. Nimesh Poudyal
International Vaccine Institute
SNU Research Park, 1 Gwanak-ro,
Gwanak-gu, Seoul, 151-742
Republic of Korea
T: +82 2 881 1343
M: +82 10 3790 9761
nimesh.poudyal@ivi.int

Biostatistics and Data Management

Dr. Deok Ryun Kim
T: +82 2 881 1140
M: +82 10 8783 6913
drkim@ivi.int

Dr. Jinae Lee
Tel: +82 2 881 1497
M: +82 10 3215 2319
Jinae.Lee@ivi.int

Hyeon Seon Ahn
Tel: +82 2 881 1394
M: +82 10 3369 7914
hyeonseon.ahn@ivi.int

Geun Hyeog Jang
Tel: +82 2 881 1138
M: +82 10 7223 9776
geunhyeog.jang@ivi.int

Jae-Woong Lee
Tel: +82 2 881 1336
M: +82 10 2043 0359

jaewoong.lee@ivi.int

International Vaccine Institute
SNU Research Park, 1 Gwanak-ro,
Gwanak-gu, Seoul, 151-742
Republic of Korea

Clinical Operations

Bo Mi Kim
Tel: +82-2-8811-279
M: +82-10-5133-6186
BoMi.Kim@ivi.int

Quality Assurance

Tobin Guarnacci
Head of Quality IVI
International Vaccine Institute
SNU Research Park, 1 Gwanak-ro,
Gwanak-gu, Seoul, 151-742
Republic of Korea
T: +82 2 881 1131
M: +82 10 9010 9959
Tobin.guarnacci@ivi.int

TABLE OF CONTENTS

TABLE OF CONTENTS

KEY ROLES 2

TABLE OF CONTENTS 6

LIST OF ABBREVIATIONS 9

STATEMENT OF COMPLIANCE 11

1 SYNOPSIS 12

2 INTRODUCTION 22

2.1 BACKGROUND 22

2.1.1 HPV GLOBAL DISEASE BURDEN 22

2.1.2 HPV-ASSOCIATED CERVICAL CANCER 22

2.1.3 OTHER HPV-ASSOCIATED DISEASES IN WOMEN 23

2.1.4 ETIOLOGICAL AGENT 24

2.1.5 PATHOGENESIS 24

2.1.6 IMMUNE RESPONSE AFTER HPV INFECTION 25

2.1.7 EXISTING HPV VACCINES 26

2.1.8 VACCINE IMMUNOGENICITY, EFFICACY AND EFFECTIVENESS 26

2.2 RATIONALE FOR THE USE OF HPV VACCINE SINGLE DOSE 30

2.2.1 INITIAL FINDINGS ON EFFICACY OF A SINGLE DOSE OF HPV VACCINE 31

2.2.2 SCIENTIFIC RATIONALE FOR USE OF SINGLE DOSE 33

2.2.3 PLANNED STUDIES AND EVALUATIONS OF SINGLE DOSE 35

2.2.4 FEASIBILITY OF MEASURING IMPACT OF VACCINATION PROGRAMS 35

2.3 HPV SITUATION IN THAILAND 37

2.3.1 HPV VACCINATION IN THAILAND 37

3 STUDY OBJECTIVES 38

4 STUDY DESIGN 39

4.1 OVERALL DESIGN 39

4.1.1 GENERAL CONSIDERATIONS 40

4.1.2 STUDY COMPONENTS 41

4.2 STUDY ENDPOINTS 46

4.2.1 PRIMARY ENDPOINT 46

4.2.2 SECONDARY ENPOINTS 46
4.2.3 EXPLORATORY ENPOINTS 47

5 SCHOOL AND POPULATION SELECTION PROCEDURES 47

6 HPV VACCINE TO BE USED IN THE STUDY 48

6.1 ACQUISITION 48
6.1.1 HPV VACCINE CHARACTERISTICS, USE AND STORAGE 48
6.1.2 VACCINE ACCOUNTABILITY 49

7 STUDY POPULATION 49

7.1 STRATEGIES FOR RECRUITMENT 49
7.2 CONSENT PROCEDURES AND DOCUMENTATION 50
7.3 COMPENSATION FOR PARTICIPATION 51
7.4 PARTICIPANT INCLUSION AND EXCLUSION CRITERIA 51
7.5 STUDY PROCEDURES 52
7.6 PARTICIPANT WITHDRAWAL FOR FURTHER VACCINATION 53
7.6.1 REASONS FOR WITHDRAWAL FOR FURTHER VACCINATION 53
7.6.2 HANDLING OF PARTICIPANT DISCONTINUATION 54
7.7 PROTOCOL DEVIATIONS 54
7.8 PROTOCOL AMENDMENTS 55
7.9 PREMATURE TERMINATION OR SUSPENSION OF STUDY 55
7.10 END OF STUDY 55

8 LABORATORY PROCEDURES AND EVALUATIONS 56

8.1 ASSESSMENT OF HPV INFECTION 56
8.2 ASSESSMENT OF IMMUNOGENICITY 57

9 SAFETY MONITORING 58

10 STUDY MONITORING 59

11 DATA MANAGEMENT AND STATISTICAL CONSIDERATIONS 59

11.1 STATISTICAL HYPOTHESIS AND SAMPLE SIZE 59
11.1.1 VACCINATION 59
11.1.2 BASELINE SURVEY AND YEAR 2 AND YEAR 4 SURVEYS 59
11.1.3 BEHAVIORAL QUESTIONNAIRE 60
11.1.4 BLOOD COLLECTION 60
11.2 DESCRIPTION OF STATISTICAL METHODS 61
11.2.1 GENERAL APPROACH 61
11.2.2 ANALYSIS OF PRIMARY ENDPOINT 61

11.2.3 ANALYSIS OF SECONDARY ENDPOINTS 61
11.2.4 EXPLORATORY ENDPOINTS 62
11.2.5 CRITERIA FOR A SECOND DOSE IN UDON THANI FEMALE STUDENTS 62

12 SOURCE DOCUMENTS AND ACCESS TO SOURCE DOCUMENTS 63

13 DATA HANDLING AND RECORD KEEPING 64

13.1 DATA COLLECTION AND MANAGEMENT RESPONSIBILITIES 64
13.2 STUDY RECORDS RETENTION 65
13.3 PUBLICATION AND DATA SHARING POLICY 65

14 QUALITY ASSURANCE AND QUALITY CONTROL 66

15 ETHICS AND PROTECTION OF HUMAN SUBJECTS 66

15.1 REGULATORY AND ETHICAL COMPLIANCE 66
15.2 PARTICIPANT AND DATA CONFIDENTIALITY 67
15.3. RESEARCH USE OF HUMAN SAMPLES 67
15.4 USE OF STORED BIOLOGICAL SPECIMENS 68

16 REFERENCES 69

17 APPENDIX 76

LIST OF ABBREVIATIONS

AEFI	Adverse Event Following Immunization
BMGF	Bill and Melinda Gates Foundation
°C	Degree Celsius
CI	Confidence Interval
CIN	Cervical intra-epithelial neoplasia
CIOMS	Council for International Organizations of Medical Sciences
COV	Cut-off value
CV	Coefficient of variation
CVT	Costa Rica Vaccine Trial
2D	two-dose regimen
DCF	Data Collection Form
DDC	Department of Disease Control
EC	Ethics Committee
EDTA	Ethylenediaminetetraacetic Acid
ELISA	Enzyme Linked Immunosorbent Assay
EPI	Expanded Program on Immunization
FAS	Full Analysis Set
FDA	Food and Drug Administration
GCP	Good Clinical Practices
GCLP	Good Clinical Laboratory Practices
GMR	Geometric Mean Ratio
GMT	Geometric Mean Titer
HPV	Human Papilloma Virus
HS	High school
IAF	Informed Assent Form
IARC	International Agency for Research on Cancer
ICF	Informed Consent Form
ICH	International Council for Harmonization
IgG	Immunoglobulin G
IRB	Institutional Review Board
IVI	International Vaccine Institute
MOP	Manual of Procedures
MOPH	Thai Ministry of Public Health
MSD	Meso Scale Discovery
MTA	Material Transfer Agreement
µg	Microgram
N	Number
NITAG	Thai National Immunization Technical Advisory Group
PCR	Polymerase chain reaction
PI	Principal Investigator
PLL	Phase-Locked Loop
PP	Per Protocol

QA	Quality Assurance
QC	Quality Control
SAP	Statistical Analysis Plan
SD	Single Dose
SOE	Schedule of Events
SOP	Standard Operating Procedure
STI	Sexually transmitted infection
TUC	Thailand US CDC Collaboration
US CDC	US Centers for Disease Control and Prevention
VE	Vaccine effectiveness
VLP	Virus-Like Particle
VS	Vocational school
WHO	World Health Organization

STATEMENT OF COMPLIANCE

The study will be conducted according to the protocol and guided by the principles of International Council for Harmonization (ICH-E6(R2)) Good Clinical Practice (GCP) Section 2, Belmont Principles, CIOMS guidelines, Declaration of Helsinki and other applicable regulations in and sponsor requirement. The Principal Investigator will ensure that no deviation from, or changes to the protocol will take place without prior agreement from the sponsor and documented approval from the Ministry of Public Health Ethical Review Committee of Research in Human Subjects (MOPH EC), Chulalongkorn University Institute Institutional Review Board (Chula IRB), and the International Vaccine Institute Institutional Review Board (IVI IRB), except where necessary to eliminate an immediate hazard(s) to the trial participants. The protocol, informed consent form(s), recruitment materials, and all participant materials will be submitted to the MOPH EC and IVI IRB for review and approval. All identified study personnel will be trained to perform their roles and will carry out their responsibilities guided by the principles of ICH GCP (section 2.0, ICH E6 (R2)) and clinic site SOPs. Roles and responsibilities of study staff are presented in the Manual of Procedures.

I agree to ensure that all staff members involved in the conduct of this study are informed about their obligations in meeting the above commitments.

Principal Investigator

Dr. Suchada Jiamsiri

____

Print/Type Name

Signed ____ Date: _________________________

Signature

1 SYNOPSIS

**Name of the Sponsor:** International Vaccine Institute (IVI)	
**Name of Investigational Product:** CERVARIX HPV vaccine manufactured by Glaxo Smith Kline (GSK)	
**Name of Active Ingredients:** Bivalent HPV vaccine presented as a suspension for intramuscular injection containing purified viral L1 protein for HPV types 16 and 18. Each 0.5 mL dose of the bivalent vaccine contains 20µg of HPV-16 L1 protein and 20 µg of HPV-18 L1 protein adsorbed onto a proprietary adjuvant system containing 500 µg of aluminum hydroxide and 50 µg of 3-O-desacyl-4-monophosphoryl lipid A (AS04).	
**Title of Study:** A community intervention effectiveness study of single dose or two doses of bivalent HPV vaccine (CERVARIX) in female school students in Thailand	
**Protocol Number:** IVI HPV1	
**Study Center(s):** Schools, provincial and district hospitals from Udon Thani and Buriram provinces, Thailand	
**Principal Investigator:** Dr. Suchada Jiamsiri, Division of AIDS and STIs,<br>Department of Disease Control, Ministry of Public Health, Nonthaburi, Thailand<br><br>**Site Manager:** Worrawan Klinsupa, Division of AIDS and STIs,<br>Department of Disease Control, Ministry of Public Health, Nonthaburi, Thailand<br><br>**Data Management and Biostatistics Scientist:** Biostatistics and Data Management Department, IVI<br><br>**Research Laboratory Scientists:** Prof. Yong Poovorawan, Dr. Sompong Vongpunswad, Pornjarim Nilyanimit, Center of Excellence in Clinical Virology, Department of Pediatrics, Faculty of Medicine, Chulalongkorn University, Bangkok, Thailand<br><br>Elizabeth Unger, Chief of Chronic Viral Diseases Branch, Division of High-Consequence Pathogens and Pathology, National Center for Emerging and Zoonotic Infectious Diseases<br>US Centers for Disease Control and Prevention, Atlanta GA, USA<br><br>**Study Oversight Monitor**: Dr. Supachai Rerks-Ngarm, Department of Disease Control<br>Ministry of Public Health, Nonthaburi, Thailand	
**Study Period**<br><br>Estimated date first participantenrolled: December 2018<br>Estimated date last participantenrolled: January 2019<br>Estimated duration of the trial: 5 years	**Phase of development:**<br>Community Intervention Effectiveness study

Study Hypothesis

Single dose (SD) of CERVARIX is able to reduce the rate of HPV infection similarly to two doses of vaccine (2D) in vaccinated female school students compared to unvaccinated students of the same school grade. The proposed study will investigate the effectiveness of a SD of HPV vaccine through the conduct of a regional effectiveness study in Thailand. The outcome from this SD effectiveness study is to generate evidence that might support deployment of SD-HPV vaccination regimen at larger scale, in both Thailand and other countries, thereby ensuring that a larger female population has access to this vaccine.

Objectives

Primary

- Demonstrate HPV vaccine effectiveness of SD by a reduction in vaccine-type HPV prevalence (HPV 16 and HPV 18) at Year 2 and Year 4 post vaccination compared to unvaccinated same grade female students (baseline survey)
- Demonstrate that HPV vaccine effectiveness of SD and 2D regimens are similar by comparing reductions in vaccine type prevalence at Year 2 and Year 4 post vaccination compared with the baseline surveys in the two provinces

Secondary

- Estimate prevalence of HPV infection in Grades 10 and 12 high school and year 1 and 3 vocational female students
- Estimate the distribution of HPV genotypes in HPV infections detected during the surveys
- Assess HPV type-specific antibody response prior and post vaccination in a subset of participants

Exploratory

- Assess sexual behavior for the comparability between the two provinces in Grades 8, Grade 10 and Grade 12 high school and year 1 and 3 vocational school female students
- Assess comparability of risk for HPV infection (HPV prevalence) between Grades 10 high school and year 1 vocational school and Grade 12 high school and year 3 vocational school female students at baseline survey and at Year 2 and Year 4 surveys post vaccination
- Assess the possible herd protection conferred by the HPV vaccination in unvaccinated female students in Year-2 survey

Methodology

The study will be initiated after obtaining approval from the Ministry of Public Health Ethical Review Committee of Research in Human Subjects (MOPH EC), Chulalongkorn University Institute Institutional Review Board (Chula IRB), and from the International Vaccine Institute Institutional Review Board (IVI IRB) guided by the principles of ICH GCP (section 2.0, ICH E6 (R2)) and ICH E11.
The study design under consideration with Thai Health Authorities includes 4 distinct and independent components: Baseline cross-sectional survey, vaccination, and sequential cross-sectional surveys for impact assessments at Year 2 and Year 4 post vaccination. Cross-sectional impact assessments at Year 2 post vaccination surveys may allow assessment of possible herd effect by enrolling female students who may not have been vaccinated. Cross-sectional impact assessment at Year 4 post vaccination survey will only enroll vaccinated schoolgirls and will focus on direct assessment of vaccine effectiveness. All these components will be conducted in schools and district hospitals from the two selected provinces. For each of the 4 components of the study, parent or guardian consent and female student consent/assent will be obtained.

The most feasible way to conduct this effectiveness study is to target young teenage female students through the public school system used for the national HPV vaccination program. The school grading system in Thailand is described below:

- Primary School, Grade 1-6: Age 7-12 years
- Secondary School, Grade 7-9: Age 13-15 years
- High School, Grade 10-12: Age 16-18 years (abbreviated Grade 10 or 12 HS)
- Vocational School, year 1-3 (corresponding to Grade 10-12): Age 16-18 years (abbreviated year 1 or year 3 VS)

About one-third of the female students attended vocational school while two-thirds attended high school.

The target population for vaccination is represented by all Grade 8 female students in the two provinces. A subset (N=200/province) of Grade 8 female students in each province will be selected and invited for a blood collection before vaccination.

From the list of all schools in the two provinces, the expected enrollment number of participants of Grade 8, Grade 10 HS/year 1 VS and Grade 12 HS/year 3 VS from each school will be determined relative to the total number of female students in each school in each province.

Target populations of surveys (baseline, Year 2 and Year 4 impact surveys) are a subset of female students of Grades 10 HS/year 1 VS and Grade 12 HS/year 3 VS from all schools in the two provinces and the sampling unit is an individual.

Due to an expected higher sexual activity among students in vocational schools, the number of participants in each school will be oversampled from vocational schools compared to high school

schools. Within each school, the planned number of survey participants will be enrolled once parent's consent and participant's consent/assent have been obtained.

The table below provides the elements of the study and the number of female students to be enrolled per each of the activities, per school grading and per province.

2018-2019		Year 2 post vaccination (2020)	Year 4 post vaccination (2022)
**Grade 10 / year 1 and Grade 12 / year 3**	**Grade 8**	**Grade 10 / year 1**	**Grade 12 / year 3**
**Cross-sectional baseline survey**	**Vaccination**	**Cross-sectional impact survey**	**Cross-sectional impact survey**
**Udon Thani**			
N = 4600*	N ~ 8000-9000	N = 2600	N = 2000
Demographics	Demographics	Demographics	Demographics
Urine collection		Urine collection	Urine collection
Behavioral questionnaire	Behavioral questionnaire (N~1500)	Behavioral questionnaire	Behavioral questionnaire
	Blood collection (N=200)	Blood collection (N=200)	Blood collection (N=200)
**Buriram**			
N = 4600	N ~ 8000-9000	N = 2600	N = 2000
Demographics	Demographics	Demographics	Demographics
Urine collection		Urine collection	Urine collection
Behavioral questionnaire	Behavioral questionnaire (N~1500)	Behavioral questionnaire	Behavioral questionnaire
	Blood collection (N=200)	Blood collection (N=200)	Blood collection (N=200)

*N=2600 for Grade 10 high school / year 1 vocational school and N=2000 for Grade 12 high school /year 3 vocational school

- Female students of Grade 8 will be enrolled after obtaining parent/guardian informed consent and participant assent. Separate informed parent/guardian consent for blood collection and participant assents from for questionnaire and blood collection will be obtained.
- Demographics will be collected from all students and sexual behavior data from a selected representative subset (N=1500 in each province).
- All enrolled students will receive either SD (Udon Thani) or 2D (Buriram).
- A baseline blood sample will be collected at the District Hospital from a subset randomly selected among those enrolled prior to vaccination (N=200 per province). Another subset of students (N=200 per province) will be randomly selected for each of the Year 2 and Year 4 surveys for blood collection for assessment of vaccine immunogenicity.

HPV infection will be determined in urine samples by PCR DNA assay by the Chulalongkorn lab. A high throughput, automated, commercially available, validated qualitative assay utilizing amplification of target DNA by PCR and nucleic acid hybridization for detection of HPV type 16 and 18 and at least 12 other types of HPV in urine samples will be used for the HPV DNA detection

assay. Positive samples will then be genotyped.

An HPV type-specific antibody assessment will be performed in a subset (N=200 per province) offemale student groups: Grade 8 prior vaccination, Grade 10/year 1 at Year 2 and Grade 12/year 3 at Year 4 post vaccination. Antibody titers against vaccine HPV 16 and 18 genotypes and non-vaccine genotypes (HPV 6, 11, 31, 33, 45, 52, 58) will be determined at the US CDC laboratory using multiplex direct IgG ELISA against L1/L2 HPV virus-like particles (VLPs).

Criteria for Inclusion/Exclusion

	Inclusion criteria	Exclusion criteria
**Grade 8 (Mathayom 2)**	• Female students with identification card<br>• Less than 15 years of age<br>• Parent or guardian consent for vaccination and blood collection as applicable Participant assent for vaccination, questionnaire, and blood collection as applicable (**Table 6**)	• Students who already received HPV vaccination<br>• Reported pregnancy<br>• Any student who has a pre-existing known medical condition or diagnosed psychological illness which in the opinion of the Principal Investigator or designee may be detrimental to her well-being**
**Baseline survey**<br><br>Grade 10 / year 1<br>Grade 12 / year 3	• Female students with identification card Participant assent for questionnaire and urine collection (**Table 6**)	• Any student who has a pre-existing known medical condition or diagnosed psychological illness which in the opinion of the Principal Investigator or designee may be detrimental to her well-being***
**Year 2 post vaccination survey**<br><br>Grade 10 / year 1<br><br>**Year 4 post vaccination survey**<br><br>Grade 12 / year 3	• Female students with identification card<br>• Parent or guardian consent for blood collection as applicable<br>• Participant consent/assent for questionnaire, urine and blood collection from those vaccinated at Grade 8 as applicable (**Table 6**)<br>• Received assigned dose regimen of HPV vaccine at Grade 8; 1 dose for Udon Thani, 2-dose for Buriram (Only for Year 4 post vaccination survey)	• Any student who has a pre-existing known medical condition or diagnosed psychological illness which in the opinion of the Principal Investigator or designee may be detrimental to her well-being***<br>• Self-reported HPV vaccination (self-purchased)<br>• Received only 1 dose or 3 doses of vaccine (for Buriram only)<br>• Received 2 doses or 3 doses of vaccine (For Udon Thani only)<br>• Received no HPV vaccine (For blood collection)

** Known anaphylaxis to the components of the HPV vaccine, allergic reaction to the first injection that may preclude a second injection,fever, symptomatic infectious disease such as urinary tract infection, lower respiratory tract infection, known blood discrasy, major psychosis, Down syndrome (some being scholarized) for behavioral questionnaire

*** Symptomatic urinary tract infection, known blood discrasy, major psychosis, Down syndrome (some being scholarized) for behavioral questionnaire

Investigational Product, Dosage and Mode of Administration

CERVARIX is a suspension for intramuscular injection containing purified viral L1 protein for HPV types 16 and 18. Each 0.5 mL dose of the bivalent vaccine contains 20µg of HPV-16 L1 protein and 20 µg of HPV-18 L1 protein adsorbed onto a proprietary adjuvant system containing 500 µg of aluminum hydroxide and 50 µg of 3-O-desacyl-4-monophosphoryl lipid A (AS04).
The vaccine must be stored at 2-8°C.

Vaccination schedule

Province	Number of Vaccinees	Months	
		0	At least 6
Udon Thani	~8000-9000	X	
Buriram	~8000-9000	X	X

Evaluation Criteria

Primary endpoint

- HPV 16 and HPV 18 DNA prevalence as measured in urine by DNA PCR at Year 2 and Year 4 post vaccination compared to unvaccinated female students

Secondary endpoints

- HPV 16 and 18 infection prevalence in Grades 10/year 1 and Grade 12/year 3 female students at baseline survey, Year 2 and Year 4 surveys post vaccination
- Distribution of HPV genotypes in HPV infections detected during the surveys. This will contribute to understand:
    - the possible cross-protection of vaccination against other HPV genotypes not targeted by the vaccine
    - to document the evolution of the frequency of circulating HPV genotypes over time and their relationship with sexual behavior
- HPV type-specific antibody response in a subset of participants prior to vaccination and at Year 2 and Year 4 post vaccination to ensure and document that the vaccine used in this study is immunogenic. The study is not powered to correlate vaccine-induced responses with effectiveness.

Exploratory Endpoints

- Sexual behavior of female students from Grades 8, 10/year 1 and 12/year 3. This will allow to compare the level of risk between provinces and between vaccinated and unvaccinated (baseline) female students at different time points.
- Comparability of risk (sexual behavior) for HPV infection (HPV prevalence) between Grades 10 high school and year 1 vocational school and Grade 12 high school and year 3 vocational school female students at baseline survey and at Year 2 and Year 4 surveys post vaccination
- Possible herd protection conferred by the HPV vaccination as indirect benefit of the vaccine in unvaccinated female students in each province in Year-2 survey

**Safety monitoring**<br><br>This is a community intervention effectiveness study of one or two doses of CERVARIX, a licensed vaccine approved by the Thai FDA since 2012. Numerous safety studies have already been conducted including in Thailand. Since the study involves a large number of female students who will be vaccinated (N=16,000-18,000), the safety events the safety events experienced by the vaccinated student will be reported to the study site staff and transmitted to the Principal Investigator for review and then entered into the database.<br><br>Adverse Event Following Immunization (AEFI) will be recorded through the national surveillance sytem and reported as per national guidelines. As per current practice in the national surveillance system, the AEFI listed below which occurred within 4 weeks after vaccination should be reported:<br>1. Result in death with unknown cause<br>2. Neurological syndrome such as seizures, muscle weakness, encephalitis, etc.<br>3. Severe allergic reaction such as anaphylaxis<br>4. Sepsis<br>5. High fever with redness and swelling at the injection site for more than 3 days.<br>6. Any events probably related to the vaccination<br>a. Hospitalization<br>b. Cluster of students with same events<br>c. Non severe symptoms such as rash and abcess at the injection site.<br><br>The PI, sub-investigators, and site staff will exercise due diligence in ascertaining, accurately recording all reportable AEFI that vaccinated study participants may have experienced. Reported AEFI will be linked to the study ID of the students. Site investigators will contact the PI and ask for appropriate management as required. AEFI may also be reported to the MOPH EC as per local regulations.
**Statistical Considerations**<br><br>The target population is composed of female students from all schools in Udon Thani and Buriram provinces and sampling unit is the student. The total sample size of Grade 8 participants (N=16,000-18,000) to receive HPV vaccination is based on the current estimate of Grade 8 students in secondary schools of Udon Thani and Buriram provinces. All willing Grade 8 students would be vaccinated in these two provinces.<br><br>We will perform random sampling including oversampling from vocational schools and regular high schools and proportional sampling by number of students in each school.<br><br>For baseline survey and Year 2 and Year 4 cross-sectional surveys, assuming a 90% vaccine coverage of Grade 8 participants, the sample size of N=2600 per province at Year 2, and N=2000 per province at Year 4 are calculated to provide >80% power to show the vaccine effectiveness (VE) of SD is non-inferior to VE of 2D with non-inferiority margin of 10% using one-sided test at 0.025 significance level. In addition, this sample size is calculated to provide 80% power to test

the vaccine effectiveness of SD or 2D is greater than 50% using one-sided test at a 0.025 significance level and 90% vaccination coverage. The assumed prevalence of HPV 16 and or 18 in Grade 10/year 1 and Grade 12/year 3 female students in the two provinces (for Year 2 and Year 4 year impact surveys, respectively) are 2% and 3% respectively. Due to lack of information on HPV prevalence in female students in Thailand, the prevalence of HPV infection in Grade 10/year 1 and Grade 12/year 3 will be estimated based on baseline survey.

The sample size for the behavioral questionnaire N=1500 is based on the current estimate of the rate of sexual risk (experience of sexual activity) as 2% [95% CI 1.4%-2.8%] and for which we expect to see about 30 sexually active Grade 8 female students per region.

For Grade 10/year 1 and Grade 12/year 3 baseline and Year 2 and Year 4 surveys, all female students enrolled in these events would be asked to fill in the questionnaire in order to link risk behavior with HPV prevalence (N=2600 for Grade 10 high school / year 1 vocational school and N=2000 for Grade 12 high school /year 3 vocational school per province).

For the assessment of HPV type-specific antibody response to the vaccine, the sample size N=200 per province is based on keeping the CV of mean titer of immunogenicity (HPV-16 or HPV 18) for SD or 2D as less than 2.5. So that the 95% CI of GMT estimate of SD or 2D can be comparable to the estimate of GMT from other studies in similar populations in Thailand.

The prevalence of HPV infection will be measured by DNA PCR in urine samples.

- To estimate the vaccine effectiveness (VE) of SD and 2D, the percent reduction of HPV 16 and 18 prevalence at the Year 2 and Year 4 impact surveys from the prevalence of Grade 10 / year 1 and Grade 12 / year 3 at the baseline cross-sectional survey will be calculated
- To establish whether the VE of SD is comparable to 2D, VE estimates at Year 4 impact survey in SD province will be tested to show non-inferiority to the estimate of VE in 2D province. Using the baseline information of two provinces, any risk factors for HPV infection such as whether the participant is from vocational school, participant is sexually active, may be considered as adjustment in the comparison.

The frequency of the different HPV genotypes detected in positive urine samples will be summarized for each assessment (baseline and Year 2 and Year 4 impact survey) in each province.

Immunogenicity assessment will be performed in a subset prior vaccination and at Year 2 and Year 4 impact assessments (N=200 per province at each assessment). Geometric mean titre (GMT) of serum ELISA antibody at Year 2 and Year 4 impact assessment after SD or 2D will be summarized.

The responses to the sexual behavior questionnaire from Grade 8, Grade 10/year 1 and Grade 12/year 3 students at each assessment in each province will be summarized descriptively and utilized to assess the potential ascertainment bias for estimates due to survey sample selection

and subsequent comparison with Grade 10/year 1 and Grade 12/year 3 female students at baseline survey at each assessment. If any critical differences are found for some variables, an adjusted analysis will be performed as sensitivity analysis.

Herd protection may be analyzed assuming that there would be sufficient unvaccinated female students at Year 2 cross-sectional survey to allow this analysis. HPV prevalence in unvaccinated students at Year 2 (Grade 10/year 1) would be compared to the HPV prevalence of same school grade unvaccinated students from the baseline survey.

2 INTRODUCTION

2.1 BACKGROUND

2.1.1 HPV GLOBAL DISEASE BURDEN

Based on a meta-analysis, the HPV prevalence worldwide among women with normal cytological findings is estimated to be 11.7% (95% confidence interval (CI) (11.6–11.7%) [1]. The highest prevalence was in sub-Saharan Africa (24%; 95% CI: 23.1–25.0%), Latin America and the Caribbean (16.1%; 95% CI: 15.8–16.4%), eastern Europe (14.2%; 95% CI: 14.1–14.4%), and South East Asia (14%; 95% CI: 13.0–15.0). However, country-specific adjusted HPV prevalence in cervical specimens ranged from 1.6% to 41.9% worldwide. Age-specific HPV prevalence peaked at younger ages (<25 years) with a prevalence of 21.8% (95% CI: 21.3–22.3%, crude) and 24.0% (95% CI: 23.5–24.5%, adjusted), with lower prevalence at middle ages. In Central and South America an increase in prevalence at older ages (≥45 years) was documented [2]. In some low-income countries in Asia and Africa, HPV prevalence is very similar in women in all age groups [3].

HPV types 16 and 18 were the most frequent types worldwide, with HPV-16 the most common type in all regions. HPV-18 and other high-risk types, such as types 31, 39, 51, 52, 56, 58, and 59, had similar prevalence and were among the most common high-risk HPV types after HPV-16. Women infected with one HPV type may be co-infected or subsequently infected with other types [4].

2.1.2 HPV-ASSOCIATED CERVICAL CANCER

Persistent infection with high-risk HPV types is strongly associated with the development of cervical cancer [5]. It was estimated that 630 000 new HPV-related cancers occurred in women in 2012, of which 530 000 (84%) were cervical cancer. This resulted in an estimated 266 000 deaths worldwide, accounting for 8% of all female cancer deaths that year [6].

HPV-16 and HPV-18 together are responsible globally for 71% of cases of cervical cancer [7]. More specifically, 60.6% (95% CI: 59.6–61.6) of cases are attributed to HPV-16 and 10.2% (95% CI: 9.6–10.9) to HPV-18. HPV-31 accounts for 3.7%, HPV-33 for 3.8%, HPV-45 for 5.9%, HPV-52 for 2.8% and HPV-58 for 2.3% of cervical cancer cases. HPV types 16, 18, 45, 31, 33, 52, and

58 account for approximately 90% of the squamous-cell carcinomas which are positive for HPV DNA [8].

While infection with a high-risk HPV type is the underlying cause of cervical cancer, most women infected with high-risk HPV do not develop cancer. Infection persists in only a small percentage of women and only a small percentage of chronic infections progress to pre-cancer, of which even fewer will progress to invasive cancer.

A large majority (>85%) of cervical cancer cases (445 000 annually) occur in the less developed regions, where it accounts for almost 12% of all cancers in women. In comparison, in more developed regions, cervical cancer accounts for less than 1% of all cancers in women (83 000 annually). Mortality rates vary 18-fold between countries, ranging from <2 per 100 000 women in industrialized countries to 28 per 100 000 in some developing countries [6].

2.1.3 OTHER HPV-ASSOCIATED DISEASES IN WOMEN

Anogenital HPV infection can result in malignant cancers or benign skin and mucosal tumours, including anogenital warts in women. Although a wide variety of HPV types can cause anogenital warts, some studies suggest that types 6 and 11 account for up to 90% of all cases [9,10], although the actual contribution of these types to developing genital warts may be lower. In a systematic review of global estimates, the overall reported annual incidence (men and women combined) of anogenital warts (including new and recurrent) ranged from 160 to 289 per 100 000. The estimated median annual incidence of new anogenital warts was 137 per 100 000 men and 121 per 100 000 women. Prevalence ranged from 0.15% to 0.18% in the general population [11].

HPV infection with certain specific HPV types is also the cause of a proportion of cancers of the anus, the oropharynx, the vulva and vagina, and of the penis. Of HPV-associated cancers, HPV types 16 and 18 are associated with 85% of HPV-related head and neck cancers and 87% of anal cancers – the second and third most frequent HPV-related cancers with, respectively, 38 000 and 35 000 estimated cases per year [12].

2.1.4 ETIOLOGICAL AGENT

Human papillomaviruses belong to the family Papillomaviridae. The virions are non-enveloped and contain a double-stranded DNA genome. The genetic material is enclosed by an icosahedral capsid composed of major and minor structural proteins, L1 and L2 respectively. These viruses are highly tissue-specific and infect both cutaneous and mucosal epithelium. Based on the genomic sequence of L1, the gene encoding the principal capsid protein, over 200 HPV types have been identified and characterized. Papillomavirus isolates are traditionally described as 'types'. HPV types may be classified in many ways, including the locations on the body that each virus tends to infect (cutaneous or mucosal types) and by their potential to induce cancer, i.e., high-risk vs. low-risk types. The International Agency for Research on Cancer currently defines 12 high-risk HPV types which are associated with cancers in humans (types 16, 18, 31, 33, 35, 39, 45, 51, 52, 56, 58, 59) and additional types for which there is limited evidence of carcinogenicity (types 68 and 73) [12,13].

2.1.5 PATHOGENESIS

HPV viruses are spread through contact with infected genital skin, mucous membranes, or bodily fluids, and can be transmitted through sexual intercourse including oral sex. Most (70–90%) of HPV infections are asymptomatic and resolve spontaneously within 1–2 years. If not detected and treated appropriately, persistent infection with high-risk types may progress o invasive carcinoma at the site of infection, mainly of the genital tract. Persistent HPV infection is a necessary cause of cervical cancer [5]. Persistent HPV infection is defined by the presence of type-specific HPV DNA on repeated clinical biological samples over a period of time, usually 6 months, although this time period is not universally accepted [12].

About 5–10% of all infected women develop persistent infection. Persistent infections, within months or years, may progress towards premalignant glandular or squamous intra-epithelial lesions, classified histo-pathologically as cervical intra-epithelial neoplasia (CIN), and to cancer. CIN is further classified as: CIN 1: mild dysplasia; CIN 2: moderate to marked dysplasia; and CIN 3: severe dysplasia to carcinoma in situ. Most CIN lesions regress spontaneously, though over a number of years, lesions on the cervix can slowly become cancerous.

The interval between the acquisition of HPV infection and progression to invasive carcinoma is usually 20 years or longer. The basis for this progression is not well understood but the predisposing conditions and risk factors include the following: HPV type; immune status (susceptibility is greater in persons who are immuno-compromised, HIV-infected, or receiving immunosuppressive therapy); co-infection with other STIs (herpes simplex, chlamydia and gonococcal infections); parity and young age at first pregnancy; tobacco smoking. HIV-infected women have a higher prevalence of persistent HPV infection, often with multiple HPV types, and are at increased risk of progression to high-grade CIN and cervical cancer compared to women without HIV infection [14].

HPV infection is also implicated in a variable range of carcinomas of the anus (88%), the vulva (15–48%, depending on age) and vagina (78%), the penis (51%) and the oropharynx (13–60%, depending on region). In all of these sites HPV-16 is the predominant type [5,15]. HPV infection with low-risk types causes anogenital warts in females (*condylomata acuminata* or venereal warts). Over 90% of these are associated with types 6 and 11. The reported median time between infection with HPV types 6 or 11 and the development of anogenital warts is 11–12 months in men and 5–6 months in young women [11]. Anogenital warts can be difficult to treat and, in rare cases, can become malignant. HPV-6 and HPV-11 can also cause a rare condition known as recurrent respiratory papillomatosis (RRP), in which warts form on the larynx or other parts of the respiratory tract with the risk of airway obstruction [16].

2.1.6 IMMUNE RESPONSE AFTER HPV INFECTION

The median time from HPV infection to seroconversion is approximately 8–12 months, although immunological response varies by individual and HPV type. HPV infections are restricted to the epithelial layer of the mucosa and do not induce a vigorous immune response [12,17]. The best-characterized and most type-specific HPV antibodies are those directed against the L1 protein of the virus. After natural infection, 70–80% of women seroconvert; their antibody responses are typically slow to develop and of low titer and avidity [18].

The available data on whether natural infection with HPV induces protection against reinfection are equivocal. There appears to be a reduced risk of reinfection with the same HPV type but infection does not seem to provide group-specific or general immune protection from reinfection

with other HPV types. In most cases, those who develop lesions mount an effective cell-mediated immune (CMI) response and the lesions regress [19].

Failure to develop an effective CMI response to clear the infection results in persistent infection and, in the case of the high-risk HPVs, an increased probability of progression to CIN 2/3 [12].

2.1.7 EXISTING HPV VACCINES

There are two highly effective (Vaccine efficacy >90%) WHO-prequalified vaccines, the quadrivalent Gardasil™ (Merck) and bivalent CERVARIX™ (GSK). A third vaccine, the 9-valent HPV vaccine (Gardasil® 9 (Merck), is undergoing WHO-prequalification now. WHO guidelines call for the two-dose vaccination of girls and women 9–14 years of age. Despite reductions in the cost of these critical vaccines through Gavi or tiered-pricing, the uptake of HPV vaccines into national programs has been limited particularly in Africa and Asia, although several countries have introduced vaccine in effectiveness studies. Barriers to uptake remain for both Gavi-eligible and graduating countries and include cost (vaccine and delivery cost), programmatic/logistical (novel age of target group, competing new vaccine introduction priorities) and socio-cultural challenges (reviewed in [12,20]).

For all HPV vaccines, the vaccination schedule stipulated by the manufacturers depends on the age of the vaccine recipient. The vaccines should be administered intramuscularly in the deltoid region [21]. The background information focuses on CERVARIX (GSK), the bivalent HPV vaccine, intended to be used for this study.

For girls and boys aged 9–14 years, a 2-dose schedule (0.5 mL at 0 and 5–13 months) is recommended. If the age at the time of the first dose is ≥15 years, 3 doses (0.5 mL at 0, 1, 6 months) are recommended. The second dose can be given between 1 and 2.5 months after first dose and the third dose between 5 and 12 months after the first dose. If, at any age, the second vaccine dose is administered before the fifth month after the first dose, the third dose should always be administered. The need for a booster dose has not been established [12].

2.1.8 VACCINE IMMUNOGENICITY, EFFICACY AND EFFECTIVENESS

The mechanism of protection conferred by HPV vaccines is assumed, on the basis of data from animal models, to be mediated by polyclonal neutralizing antibodies against the major viral coat

protein, L1. In clinical trials of the vaccines, a peak antibody titer was observed 4 weeks after the third dose and declined within the first year, then stabilized at a plateau titer after 18 months. The serological response after vaccination is much stronger (1–4 logs higher) than the response after natural infection. The reasons are unclear but may be related to better targeting/activation of lymph node cells by vaccines than by mucosal infections, and possibly by the use of adjuvants in the existing vaccines. Long-lived plasma cells, which primarily reside in the bone marrow, continuously produce IgG antibodies and are responsible for long-term HPV-specific antibody persistence [22].

Vaccine-induced circulating antibodies are thought to reach the site of infection by active IgG transudation in the female genital tract. Immunization also elicits memory B cells though their contribution to long-term protection is unclear [23]. Protective efficacy depends upon the quantity but also the quality (affinity) of vaccine-induced antibodies [24].

Breakthrough cases in vaccinated individuals have not yet been unequivocally identified in clinical studies, precluding the identification of a minimal antibody threshold level that would correlate with protection against CIN 2 or 3, or against persistent infection. Thus no specific immune correlate is yet available. The specific assays that have been developed to evaluate the immune response include: VLP-based enzyme immunoassay, competitive immunoassay with labelled neutralizing monoclonal antibodies, and in vitro neutralization [12].

HPV vaccines were licensed on the basis of their clinical efficacy in young adult women for the three-dose vaccine. The age extension for pre-adolescent and adolescent girls and boys for all 3 vaccines, in whom efficacy trials would not be feasible (due to ethical considerations and follow-up time from infection to development of detectable lesions), was granted because studies demonstrated that antibody responses in adolescent girls were not inferior to those elicited in women (immunological bridging).

2.1.8.1 THREE-DOSE SCHEDULE

All 3 HPV vaccines were originally licensed and marketed using a 3-dose vaccination schedule. Gardasil-9 has a two-dose regimen in its US FDA package insert, but Gardasil-4 and CERVARIX package inserts only mention 3-dose regimen. However, WHO-SAGE recommendation supports

a 2D schedule. Approval of the 2D indication was based on demonstration of non-inferiority of the antibody response when compared to young adult women in whom efficacy has been proven.

Efficacy of the bivalent vaccine has been evaluated in 2 Phase III studies [25,26]. In both studies high efficacy was observed against infection and cervical lesions associated with HPV-16 and HPV-18 in women not already infected with HPV (HPV-naive) [25-27].

The immunogenicity of the bivalent and quadrivalent vaccines was compared in a head-to-head trial. Neutralizing antibodies against HPV-16 and HPV-18 were 3.7 and 7.3-fold higher, respectively, for the bivalent compared to the quadrivalent vaccine in women aged 18–26 years at month 7 after initiation of the vaccination course [28]. After 60 months of follow-up, geometric mean titres (GMTs) were consistently higher in those receiving the bivalent vaccine across all age strata (18–45 years): 2.3–7.8-fold higher for HPV-16 and 7.8–12.1-fold higher for HPV-18 [29]. However, the clinical relevance of these findings is unclear as no correlate of protection has been defined.

2.1.8.2 TWO-DOSE SCHEDULE

Results of a systematic review indicate that immunogenicity of 2 doses of HPV vaccine in girls aged 9–14 years are non-inferior to 3 doses in women aged 15–24 years (cited in [12]). In 4 randomized studies (1 of quadrivalent, 2 of bivalent, and 1 of nonavalent vaccine) [30-34], and 2 non-randomized trials [35,36] (of quadrivalent and bivalent vaccines), immunogenicity outcomes were compared using a 2D schedule (0, 6 months, and some with a 0, 12 months arm) in girls aged 9–14 years and a 3-dose schedule (0, 1 or 2, 6 months) in young women aged 15–26 years. Two doses in girls were non-inferior to, or resulted in higher GMTs, than 3 doses in young women (for the nonavalent, all 9 HPV types measured except HPV-45, non-inferiority inconclusive).

Post-hoc analyses of two- or three-dose trials found high vaccine effectiveness following a single dose of bivalent or quadrivalent vaccine [37-39]. However, the interpretation of analyses that included women with incomplete vaccination schedules is limited by several factors including that women were not randomized by number of doses, the sample size, and the low number of incident or persistent infections.

Current evidence suggests that the three licensed HPV vaccines would have relatively similar effectiveness in preventing cervical cancer [12]. Regarding the impact of vaccination programmes at the population level, there is evidence that vaccination significantly reduces the prevalence of high-risk HPV types among young women [40].

2.1.8.3 CURRENT WHO RECOMMENDATION

The current evidence supports the recommendation for a 2-dose schedule with adequate spacing between the first and second dose in those aged 9–14 years. This schedule also has cost-saving and programmatic advantages that may facilitate high coverage. For HPV vaccines, a 2-dose schedule with a 6-month interval between doses is recommended for individuals receiving the first dose before 15 years of age. Those aged >15 years at the time of the second dose are also adequately covered by 2 doses. There is no maximum recommended interval between doses. However, an interval no greater than 12–15 months is suggested in order to complete the schedule promptly and before becoming sexually active. If the interval between doses is shorter than 5 months, a third dose should be given at least 6 months after the first dose [20].

2.1.8.4 CROSS-PROTECTION

With regard to cervical cancer prevention, all of the 3 licensed HPV vaccines provide high protection against HPV-16 and HPV-18, the HPV types which are associated with 71% of cervical cancer cases globally. HPV vaccines provide some cross-protection against HPV types not included in the vaccines. Based on evidence from clinical trials and post-introduction impact evaluations, the bivalent and quadrivalent HPV vaccines provide some level of cross-protection against high-risk HPV types other than 16 and 18, in particular for types 31, 33 and 45 [12,41-43].

HPV types 31, 33 and 45, the 3 types against which the bivalent and quadrivalent vaccines are reported to give cross-protection, are associated with 13% of cervical cancer cases. A systematic review evaluated changes between pre- and post-vaccination periods in infection rates of high-risk HPV types other than types 16 and 18 [44]. Evidence of cross-protection was found for HPV-31 (prevalence ratio=0.73 [95% CI: 0.58–0.92]) but little evidence of cross-protection for HPV-33 and HPV-45 (prevalence ratio=1.04 [95% CI: 0.78–1.38] and 0.96 [95% CI: 0.75–1.23]). Subsequent analyses of post-introduction evaluations show bivalent vaccine to have cross-protection against all three types [45,46].

2.1.8.5 DURATION OF PROTECTION

For the bivalent vaccine, immunogenicity and efficacy of a 3-dose schedule against infection and cervical lesions associated with HPV-16 and HPV-18 have been demonstrated up to 8.4 and 9.4 years respectively [47]. Antibody levels reached following a 2-dose schedule (0, 6 months) of the bivalent in girls aged 9–14 years remained comparable to those with a 3-dose schedule in women up to 5 years after first vaccination, indicating similar decay kinetics. It is not yet known whether booster doses will be required after the primary immunization. Based on data up to 9.4 years after the primary 3-dose immunization series with bivalent vaccine, there is no evidence that efficacy against infection and CIN 1+ lesions associated with HPV-16 or HPV-18 wanes over time (cited in [12]).

2.2 RATIONALE FOR THE USE OF HPV VACCINE SINGLE DOSE

Despite reductions in the cost through Gavi or tiered-pricing, the uptake of HPV vaccines into national programs has been limited. Barriers to uptake remain for both Gavi-eligible and graduating countries and include cost (vaccine and delivery cost), programmatic and logistical (novel age of target group, competing new vaccine introduction priorities), as well as socio-cultural challenges.

Vaccination targeting multiple age cohorts of female students aged between 9 and 18 years at the time of HPV vaccine introduction would result in faster and greater population impact than vaccination of single age cohorts, due to the estimated increase in direct protection and herd immunity [48]. This approach should also offer opportunities for economies of scale in delivery and could make programmes more resilient to any interruptions in vaccine supply. Vaccination of multiple cohorts of girls is cost-effective in the age range 9–14 years. The initial vaccination of multiple cohorts of girls aged 9–14 years is recommended when the vaccine is first introduced [20].

The use of a single dose (SD) of HPV vaccine would enable twice as many female students to benefit, facilitate catch-up vaccination campaigns and alleviate the overall economic cost of vaccine supply and delivery and treatment costs of disease. However, WHO acknowledges that more data are needed to issue such recommendation [12,20].

2.2.1 INITIAL FINDINGS ON EFFICACY OF A SINGLE DOSE OF HPV VACCINE

Recently, post-hoc evidence from three multi-dose trials has provided strong evidence that a SD of either vaccine may also have high and sustained efficacy.

Data from the Costa Rica Vaccine Trial (CVT; NCT00128661) and the PApilloma TRIal against Cancer In young Adults (PATRICIA; NCT001226810), two Phase III controlled, randomized, double-blind, clinical trials of the HPV-16/18 AS04-adjuvanted vaccine CERVARIX among young women, were combined in a *post-hoc* analysis to investigate efficacy of fewer doses of the HPV-16/18 vaccine after four years of follow-up. In these studies, women were randomly assigned to receive three doses of the HPV-16/18 vaccine or to a control vaccine; yet some received fewer doses. After excluding women with <12-months follow-up or those HPV16/18 DNA-positive at enrollment (for the HPV16/18 endpoint), vaccine efficacy was calculated against one-time detection of incident HPV infections after three (n=11,110 HPV and 11,217 control), two (n=611:574), and one (N=292:251) dose(s). The main aim of the analysis was to ascertain HPV16/18 vaccine efficacy as well as to explore protection conferred against non-vaccine HPV types, by number of doses received.

Table 1. Summary of Vaccine Efficacy by number of doses received in combined post-hoc analysis of CVT and PATRICIA [37,49]

Number of Doses	Number of Subjects (vaccine/control)	Vaccine efficacy against vaccine serotypes (16/18)	Vaccine efficacy against non-vaccine serotypes (31/33/45)
Three	11,110 / 11,217	77%	59%
Two	611 / 574	76%	37%
One	292 / 251	85%	36%

Vaccine efficacy against incident HPV16/18 infections for three doses was 77.0%, two doses was 76·0%, and one dose was 85.7%. Vaccine efficacy against incident HPV31/33/45 infections for three doses was 59·7%, two doses 37.7%, and one dose was 36.6%. However, two-dose women who received their second dose at six months, but not those receiving it at one month, had efficacy estimates against HPV 31/33/45 similar to the three-dose group (Vaccine efficacy 68.1%; CVT data only). Four years following vaccination of women aged 15 to 25 years, one and two dose(s)

of the HPV16/18 vaccine appear to protect against cervical HPV16/18 infections, similar to the protection provided by the three-dose schedule. Two doses separated by six months additionally provided limited cross-protection. These data argue for a direct evaluation of one-dose efficacy of the HPV16/18 vaccine [37].

A 17,729-girl study conducted in nine locations in India provide additional support for the efficacy of single dose HPV vaccine; data are available on immunogenicity and HPV infection after one, two, and three doses of quadrivalent HPV vaccine Gardasil® among girls vaccinated at ages 10-18 years. The original study was a randomized trial of two vs. three doses; however, the study was stopped for reasons unrelated to the vaccine and not all girls received the full schedule. Therefore, there are girls aged 10–18 years who received three doses of vaccine on days 1, 60, and 180 or later, two doses on days 1 and 180 or later, two doses on days 1 and 60 by default, and one dose by default. The primary outcomes were immunogenicity in terms of L1 genotype-specific binding antibody titers, neutralizing antibody titers, and antibody avidity after vaccination for the vaccine-targeted HPV types 16, 18, 6, and 11 and incident and persistent HPV infection. Analysis was per actual number of vaccine doses received. Of 21,258 eligible girls identified at 188 clusters, 17,729 girls were recruited from 178 clusters before suspension. 4348 (25%) girls received three doses, 4979 (28%) received two doses on days 1 and 180 or later, 3,452 (19%) received two doses at days 1 and 60, and 4,950 (28%) received one dose. Antibody response in the two-dose HPV vaccine group was non-inferior to the three-dose group at 7 months, but was inferior in the two-dose default and one-dose default groups at 18 months. The geometric mean avidity indices after fewer than three doses by design or default were non-inferior to those after three doses of vaccine. Fewer than three doses by design and default induced detectable concentrations of neutralizing antibodies to all four vaccine-targeted HPV types, but at much lower concentration after one dose. Cervical samples from 2,649 participants were tested and the frequency of incident HPV 16, 18, 6, and 11 infections was similar irrespective of the number of vaccine doses received. The testing of at least two samples from 838 participants showed that there were no persistent HPV 16 or 18 infections in any study group at a median follow-up of 4.7 years [39].

A recent summary of the longer term follow-up of the above studies is presented in **Table 2** below (Peter Dull, personal communication, Bill & Melinda Gates Foundation).

Table 2. Longer term follow-up of CVT and IARC India studies

CVT Study (Costa Rica 2004)	
Vaccine	CERVARIX (GSK)
Study Design	RCT; subset analysis of non-completers
Subjects contributing to 1-dose efficacy	196 and 134 subjects at 4 and 7 years of follow-up, respectively
Efficacy summary	100% (No persistent infection among 1-dose subjects vs. 8% among controls)
Immunogenicity summary	7 year post vaccination immune response stable and ~5-fold above natural infection

IARC Study (India 2009)	
Vaccine	Gardasil (Merck)
Study Design	Cluster randomized; enrollment halted and new 1-dose group created
Subjects contributing to 1-dose efficacy	749 subjects at 5 years of follow-up
Efficacy summary	No evidence of breakthrough infection with HPV-16/18
Immunogenicity summary	5 years post vaccination immune response stable

2.2.2 SCIENTIFIC RATIONALE FOR USE OF SINGLE DOSE

Although the antigens in the HPV vaccines are designated “virus-like particles” (VLPs) because they mimic the outer shell of authentic HPV virions, they are generally considered to be a type of subunit vaccine in that they are composed of a single highly purified protein, in this case the L1 major capsid protein, and are entirely noninfectious.

The most compelling support for the conjecture that HPV vaccines will induce long term protection after a single dose are the findings that the vaccines induce strong and durable neutralizing antibody responses against the targeted types in essentially all one dose recipients. For the bivalent HPV vaccine, the geometric mean of antibody titers (GMT) after four years were only

four-fold lower in one dose compared to three-dose recipients [50], and this ratio has been maintained out to seven years [51]. The exceptional immunogenicity of HPV vaccines can largely be attributed to the structure of the HPV vaccine antigen. Typical subunit vaccines are composed of monomeric or low valence multimeric proteins or carbohydrate/protein conjugates. In contrast, HPV VLPs are composed of 360 ordered protein subunits that form a particulate 55nm structure displaying a repetitive array of epitopes on their surface. The engagement of these repetitive elements by the B-cell receptors on naive B cells is believed to transmit exceptionally strong activation and survival signals to the cells leading to consistent induction of memory B cells, and, most importantly, long-lived plasma cells that continuously produce antibodies for many years [52]. Epitope spacing of 50-100 Å appears to be critical. This spacing is commonly found on microbial surfaces but not on body surfaces normally exposed to the systemic immune system. The particulate and repetitive structure of VLPs likely contributes to B-cell immunity in several additional ways. Particles of this size efficiently traffic to lymph nodes and are also efficiently phagocytized by antigen presenting cells for the initiation of immune responses and the generation of cognate T-helper cells. The polyvalence of VLPs also leads to stable binding of nature low avidity IgM and complement, promoting their acquisition by follicular dendritic cells, which are key components in inducing humoral immune responses in the lymph node. While the hepatitis B vaccine is also considered a VLP it appears to be much less immunogenic after a single dose, perhaps because it has a fewer subunits and/or because they float in a lipid bilayer. To summarize, by mimicking the key structural features of authentic viruses, the HPV VLPs consistently induce potent and long lasting humoral responses that more closely resemble the anti-virion responses to an acute virus infection or a single dose of a live-attenuated virus vaccine than the response to simple subunit vaccines.

Several virologic factors also likely contribute to the exceptional efficacy of the HPV vaccines. First, HPVs have an unusual life cycle that is entirely confined to a stratified squamous epithelium. By producing their virions in the superficial layers and shedding them from the epithelial surface, the viruses minimize exposure of these highly immunogenic structures to the systemic immune system, and thereby can persistently produce infectious virions that are not subject to inactivation by neutralizing antibodies. Overall, HPVs have evolved to maintain immune ignorance rather than evolving mechanisms to actively counteract systemic immunity. This mechanism of immune escape can be easily overcome by parenteral injection of the VLPs. Second, studies in animal models have found that the mechanism that the papilloma viruses use to infect their target tissues make them exceptionally susceptible to vaccine-induced virion neutralizing antibodies [53]. To

initiate infection, the virions must bind to specifically modified forms of heparan sulfate restricted to the basement membrane in normal tissue. Exposure of the basement membrane requires epithelial disruption. Direct exudation of systemic antibodies occurs at these sites, such that the virions are subject to an increasing gradient of antibody concentration as they approach their binding site. Basement membrane binding initiates a series of conformational changes that are required for exposure of the keratinocyte receptor-binding site on the virion surface. Importantly, this process and the subsequent process of virion internalization by the basal keratinocyte take several hours. The slow kinetics of infection, much slower than for other well-characterized viruses, provides an exceptional length of time for vaccine-induced antibodies to disrupt the process. Inactivation can occur even after basement membrane binding, perhaps due to Fc receptor-mediated phagocytosis of the virion/antibody complex. Neutrophils and macrophages are specifically attracted to sites of epithelial disruption. Experiments in mice involving passive transfer of sera from VLP vaccine individuals into naive recipients indicate that levels of circulating antibodies that are 100-fold lower than the minimum detected in in vitro assays are sufficient to protect from high-dose cervicovaginal challenge in vivo, implying that there are potent mechanisms of antibody-mediated infection inhibition that are not measured in vitro [54]. In light of these observations, it is not surprising that the four-fold lower longterm antibody titers in one-dose recipients did not diminish the apparent protective efficacy of the HPV vaccines.

2.2.3 PLANNED STUDIES AND EVALUATIONS OF SINGLE DOSE

In order to definitively establish the efficacy of a SD of HPV vaccine, a randomized trial is being conducted to compare 1-dose (SD) and 2-dose (2D) schedules of bivalent and 9-valent HPV vaccines in Costa Rica. This definitive evidence regarding the efficacy of SD is expected in 7 years. In addition, there are ongoing immuno-bridging studies (DoRIS and HANDS) to establish whether SD produces immune responses likely to be protective in young girls in sub-Saharan Africa. Thousands of infections resulting in cancers will occur while waiting for the results of these trials. While SAGE is unlikely to make a recommendation for SD use without additional prospectively collected data, current evidence regarding SD efficacy is sufficient to justify further effectiveness studies in willing and appropriate countries. The ultimate outcome from this SD Impact study is to accelerate the availability of evidence to support further consideration of SD strategies.

2.2.4 FEASIBILITY OF MEASURING IMPACT OF VACCINATION PROGRAMS

Demonstrating impact of HPV vaccination programs within the first few years after introduction has been shown to be feasible. To measure impact it is not necessary to wait for CIN as an endpoint as it is now accepted that impact can be measured using HPV infection and persistent infection as a surrogate [55]. A systematic review and meta-analysis was performed on 20 eligible HPV (either quadrivalent or bivalent) vaccine studies undertaken in nine high-income countries and representing more than 140 million person-years of follow-up. The vaccine regimens used were 3-dose regimens recommended by the manufacturer and impact was evaluated at 4 years after introduction. In countries with female vaccination coverage of at least 50%, HPV type 16 and 18 infections decreased significantly between the pre-vaccination and post-vaccination periods by 68%, and anogenital warts decreased significantly by 61% in girls 13–19 years of age. Significant reductions were also recorded in HPV types 31, 33, and 45 in this age group of girls, which suggests cross-protection. Additionally, significant reductions in anogenital warts were also reported in boys younger than 20 years of age and in women 20–39 years of age, which suggests a herd effect. In countries with female vaccination coverage lower than 50%, significant reductions in HPV types 16 and 18 infections and in anogenital warts occurred in girls younger than 20 years of age, with no indication of cross-protection or herd effect [40,56].

In the United States, HPV vaccine was recommended in 2006 for routine vaccination of females aged 11–12 years. To evaluate vaccine impact of quadrivalent vaccine, HPV DNA types was analyzed in in self-collected cervico-vaginal specimens and demographic, sexual behavior, and self-reported vaccination data from females 14–34 years old. Among 14 to 19-year-olds, quadrivalent vaccine-type prevalence decreased from 11.5% in 2003–2006 to 3.3% in 2011–2014, when ≥ SD coverage was 55%. Among 20-24-year-olds, prevalence decreased from 18.5% in 2003–2006 to 7.2% in 2011–2014, when ≥ SD coverage was 43%. Compared to 2003–2006, quadrivalent vaccine type prevalence in sexually active 14- 24-year-olds in 2011–2014 decreased 89% among those vaccinated and 34% among those unvaccinated. Vaccine effectiveness was 83%. Within 8 years of vaccine introduction, quadrivalent-type prevalence decreased 71% among 14 to 19-year-olds and 61% among 20- 24-year-olds [57].

The existing evidence is compelling that SD administration of HPV vaccines has substantial efficacy, and that measuring the impact of HPV vaccine introduction is feasible within 2-4 years if vaccinated girls have reached the age of sexual activity. This proposed effectiveness study would generate further country/context specific evidence with effectiveness outcomes in parallel with the accumulation of evidence from the ongoing randomized clinical trials. If initiated in 2018/19, 2-

year effectiveness data from Thailand could be available as early as 2021 with 4-year data available by 2023. Effectiveness data from different settings together with clinical trial data would create a compelling body of evidence to inform advisory bodies in 2023 in considering policy recommendations regarding SD HPV vaccine.

2.3 HPV SITUATION IN THAILAND

A review was conducted to evaluate the HR-HPV genotype distribution among Thai women with precancerous cervical lesions i.e., cervical intraepithelial neoplasia grade 2-3 (CIN 2-3), adenocarcinoma in situ (AIS), and invasive cervical cancer by reviewing the available literature. The prevalence of HR-HPV infection among Thai women with CIN 2-3 ranged from 64.8% to 90.1% and the three most common genotypes were HPV 16 (38.5%), HPV 58 (20.0%), and HPV 18 (5.5%). There were high squamous cell carcinoma/CIN 2-3 prevalence ratios in women with CIN 2-3 infected with HPV 33 and HPV 58 (1.40 and 1.38, respectively), emphasizing the importance of these subtypes in the risk of progression to invasive cancer among Thai women. Data regarding the prevalence and genotype distribution of HR-HPV in Thai women with AIS remain unavailable. Interesting findings about the distribution of HPV genotype in cervical cancer among Thai women include: (1) a relatively high prevalence of HPV 52 and HPV 58 in invasive squamous cell carcinoma; (2) the prevalence of HPV 18-related adenocarcinoma is almost double the previously reported prevalence, and (3) 75% of neuroendocrine carcinomas are HPV18-positive when taking into account both single and multiple infections [58]. Other studies have been conducted in urban and suburban areas in women with pre-cancerous lesions and showing disparate results regarding genotype distribution [59-62]. Data on HPV prevalence and genotypes in young female students is not available.

2.3.1 HPV VACCINATION IN THAILAND

Thailand first introduced the 2D-HPV vaccination regimen in some regions of the country in 2014 and has now expanded to a nationwide introduction in primary grade 5 school students with more than 95% vaccinated, which suggests an excellent acceptance of the vaccination by both parents and adolescents. The national recommendation for school students more than 15 years of age is to administer 3 vaccine doses. The vaccine which was used in nationwide introduction in 2017

was CERVARIX. According to Thai National Immunization Technical Advisory Group (NITAG) recommendation followed for the National HPV Vaccination Program, the second dose is the booster dose which can be administered at any time from 6 months onward (which applies for Year 2 and Year 4 as well). The study follows the NITAG recommendation rather than the package insert.

Buriram and Udon Thani provinces already benefit of the national HPV vaccination rollout program in 2017. Due to limited funding, only grade 5 female school students are vaccinated. There is no catch-up program for older age groups that has been implemented yet in Thailand. The present study will provide HPV vaccination to grade 8 students, not covered by the National HPV vaccination program. Therefore, a SD-strategy that would allow expanding HPV immunization to more young women while not increasing (or even reducing) costs would be extremely attractive, provided the long-term effectiveness is similar. A SD vaccination strategy might be then recommended by NITAG and adopted by MOPH.

The proposed study will investigate the effectiveness of a SD of HPV vaccine through the conduct of a regional effectiveness study in Thailand. The outcome from this SD effectiveness study is to generate evidence that might support deployment of SD-HPV vaccines at larger scale, in both Thailand and other countries, thereby ensuring that a larger female population has access to this vaccine.

3 STUDY OBJECTIVES

Primary objectives

- Demonstrate HPV vaccine effectiveness of SD by a reduction in vaccine-type HPV prevalence (HPV 16 and HPV 18) at Year 2 and Year 4 post vaccination compared to unvaccinated same grade female students (baseline survey)
- Demonstrate that HPV vaccine effectiveness of SD and 2D regimens are similar by comparing reductions in vaccine type prevalence at Year 4 post vaccination compared with the baseline surveys in the two provinces

Secondary objectives

- Estimate prevalence of HPV infection in Grades 10 and 12 high school and year 1 and 3 vocational female students
- Estimate the distribution of HPV genotypes in HPV infections detected during the surveys
- Assess HPV type-specific antibody response prior and post vaccination in a subset of participants

Exploratory objectives

- Assess sexual behavior for the comparability between the two provinces in Grades 8, high school Grade 10 and Grade 12 and vocational school year 1 and 3 female students
- Assess comparability of risk for HPV infection (HPV prevalence) between Grades 10 high school and year 1 vocational school and Grade 12 high school and year 3 vocational school female students at baseline survey and at Year 2 and Year 4 surveys post vaccination
- Assess the possible herd protection conferred by the HPV vaccination in unvaccinated female students in Year 2 survey

4 STUDY DESIGN

4.1 OVERALL DESIGN

The areas considered for this study are secondary and vocational schools from Buriram and Udon Thani provinces in the North-East region of Thailand. The selection of these two provinces was made after in-depth discussion with the MOPH representatives and is based on several considerations: accessibility, high degree of acceptance by local authorities and communities, size of the female student population and breakdown between secondary school and vocational schools, and comparability of sexual activity among female school age students (see further below). Neither of these provinces had organized HPV vaccination programs prior to 2017.

The two provinces, Udon Thani and Buriram, were selected because of the size of schoolgirl population, similar socio-economic characteristics and high likelihood of acceptability of HPV vaccination. Importantly, these two provinces were selected for their capacity and infrastructures and communication facilities to carry out the study with the highest probability of success. Buriram and Udon Thani Provinces share similar socio-economic and sexual behavior patterns [63]. Both provinces have ~80 secondary schools and ~17-26 vocational schools with sufficient female students for the purpose of the study.

4.1.1 GENERAL CONSIDERATIONS

The most feasible way to conduct this effectiveness study is to target young teenage female students through the public school system used for the national HPV vaccination program. The school grading system in Thailand is described below:

- Primary School, Grade 1-6: Age 7-12 years
- Secondary School, Grade 7-9: Age 13-15 years
- High School, Grade 10-12: Age 16-18 years (abbreviated Grade 10 or 12 HS)
- Vocational School, year 1-3 (corresponding to Grade 10-12): Age 16-18 years (abbreviated year 1 or year 3 VS)

About one-third of the female students attended vocational school while two-thirds attended high school. The fraction of female students who are sexually active varies with age and the type of school they attend, secondary vs. vocational school. According to the results of the most recent MOPH sexual behavioral survey (2017), sexual activity was higher in vocational school students (~40%) than in high school students (~18%), while it was ~ 3-6% in Grade 8 students.

A proxy indicator for sexual activity resulting in HPV infection (although due to infection with other genotypes such as HPV 6 and 11) may be given by the number of new cases of *Condyloma acuminta* per 100,000 female students per age group (MOPH data, **Table 3**):

Table 3. New cases of *Condyloma acuminta*/100,000 female students (2017)

Province	15-16 yrs of age	17-18 yrs of age
Buriram	9.5	18
Udon Thani	5	37

School calendar runs from May to March for all types of schools. Examination period takes place in March. School attendance in Thailand is generally very high. However, in later grades of secondary school and vocational school, there can be up to 15% drop out prior to graduation (Grade 12 HS and year 3 VS). In 2015, 67.3% of grade 9 female students went onto Grade 10 HS and 32.7% to vocational school. Beyond 18 years of age, a sizable fraction of female students

would move out of their home area (to university for some, to other areas for jobs for others) making follow-up difficult.

4.1.2 STUDY COMPONENTS

The study design under consideration with Thai Health Authorities includes 4 distinct and independent components: Baseline cross-sectional survey, vaccination, and sequential cross-sectional surveys for impact assessments at Year 2 and Year 4 post vaccination. Vaccination not only reduces the incidence of disease in those immunized but may also indirectly protect unvaccinated susceptible individuals against infection (herd effect).

Cross-sectional study design is the easiest design, as it does not require extensive interventions to retain a cohort over a number of several years, at a time when girls are leaving primary school. Importantly, a cross-sectional design would also allow herd protection to be assessed. The hypothetical high dropout rate would be balanced between groups and it does not matter in cross-sectional studies. A cohort design would not allow measuring potential herd protection, while cross-sectional studies allow to enroll at Year 2 post vaccination survey for students who may not have been vaccinated when attending grade 8.

Cross-sectional impact assessment at Year 2 post vaccination survey allows to assess a possible herd effect by enrolling female students who may not have been vaccinated. Cross-sectional impact assessment at Year 4 post vaccination survey will only enroll vaccinated schoolgirls and will focus on direct assessment of vaccine effectiveness. All these components will be conducted in schools (as described above) and district hospitals from the two selected provinces. For each of the 4 components of the study parent consent and female student assent will be obtained as detailed in Section 7.2. **Table 4** provides the study components over time and interventions to be performed.

Table 4. Cross-sectional survey design and scheduled interventions

2018-2019		Year 2 post vaccination (2020)	Year 4 post vaccination (2022)
Grade 10 / year 1 and Grade 12 / year 3	Grade 8	Grade 10 / year 1	Grade 12 / year 3
Cross-sectional baseline survey	Vaccination	Cross-sectional impact survey	Cross-sectional impact survey

Udon Thani			
N ~ 4600*	N ~ 8000-9000	N ~ 2600	N ~ 2000
Demographics	Demographics	Demographics	Demographics
Urine collection		Urine collection	Urine collection
Behavioral questionnaire	Behavioral questionnaire (N~1500)	Behavioral questionnaire	Behavioral questionnaire
	Blood collection (N=200)	Blood collection (N=200)	Blood collection (N=200)
**Buriram**			
N ~ 4600*	N ~ 8000-9000	N ~ 2600	N ~ 2000
Demographics	Demographics	Demographics	Demographics
Urine collection		Urine collection	Urine collection
Behavioral questionnaire	Behavioral questionnaire (N~1500)	Behavioral questionnaire	Behavioral questionnaire
	Blood collection (N=200)	Blood collection (N=200)	Blood collection (N=200)

*N=2600 for Grade 10 high school / year 1 vocational school and N=2000 for Grade 12 high school /year 3 vocational school

4.1.2.1 VACCINATION

The study target population for HPV vaccination is grade 8 female students. Our main objective is to assess vaccine effectiveness in students attending Grade 10 high school/year 1 vocational school and Grade 12 high school/year 2 vocational school. All school students present at school and whose parents/LAR have granted signed consent and who provided assent would be vaccinated. There would be no discrimination based on risk, whatever the way it is defined.

In the selected two provinces of the study, open label vaccination will be offered to all Grade 8 female students from all schools. The ideal window in the school year for vaccination and survey activities is November through February as it avoids conflict with other school-based national programs and the national exam period in March. The immunization schedule is shown in **Table 5** below.

Udon Thani Province SD

Female students in Grade 8 who did not receive any prior HPV vaccination will receive **1 dose (SD)** of CERVARIX as catch-up vaccination strategy through a school-based enrollment. The criteria for potentially administering of a second dose of vaccine to female students are developed in section 11.2.5.

Buriram Province 2D

Female students in Grade 8 who did not receive any prior HPV vaccination will receive **2 doses** of CERVARIX at least 6 months apart as catch-up vaccination strategy through a school-based enrollment.

Table 5. Immunization Schedule

Province	Number of Vaccinees	Months	
		0	At least 6
**Udon Thani**	~8000-9000	X	
**Buriram**	~8000-9000	X	X

- Female students of Grade 8 will be enrolled after obtaining parent informed consent and participant assent. Separate informed parent consent for blood collection and assents for questionnaire and blood collection will be obtained (See Section 7.2).
- Demographics will be collected from all students and sexual behavior data from a selected representative subset (N=1500 in each province)
- All enrolled students will receive either SD or 2D as specified above.
- A baseline blood sample will be collected at the District Hospital from a subset of those enrolled prior to vaccination (N=200 per province).

4.1.2.2 BASELINE CROSS-SECTIONAL SURVEY

HPV prevalence data are not available in Grades 10 and 12 HS and year 1 and 3 VS female students. We will therefore conduct a baseline cross-sectional survey to assess HPV prevalence in Grade 10 and Grade 12 HS and year 1 and 3 VS female students. Importantly, these data are needed for the comparison of HPV prevalence at Year 2 (Grade 10 HS / year 1 VS) and Year 4 (Grade 12 HS / year 3 VS) post vaccination cross-sectional surveys in each province. These data will contribute to the later adjustments of the sample size calculation for the assessments at Year 2 and Year 4 post vaccination.

Demographics, sexual behavior and urine samples for HPV prevalence (N=4,600 per province; (N=2600 for Grade 10 HS/year 1 VS and N=2000 for Grade 12 HS/year 3 VS) in both provinces will be collected as shown in **Table 4** above.

This baseline survey must be of adequate sample size to provide a robust estimate of HPV prevalence across Grade strata targeted for subsequent impact assessments. The Grade-specific HPV prevalence will be the basis for sample size calculations in subsequent assessments of Grade 10 HS / year 1 VS and Grade 12 HS/ year 3 VS post vaccination as the exposure risk changes with age.

- Female students of secondary Grade 10 and Grade 12 HS and year 1 and year 3 VS will be enrolled after obtaining a single participant assent to perform questionnaire and urine collection.
- Demographic and behavioral data will be collected from all baseline survey participants.
- Urine sample will be collected at school for HPV DNA PCR and genotyping from all participants.
- The number enrolled for this survey will be N=2600 per province for Grade 10 HS /year 1 VS and N=2000 per province for Grade 12 HS/year 3 VS, for a total of N=9200 per province.

There are however some potential limitations in using data from Grade 10 and Grade 12 HS and year 1 and year 3 VS obtained during the baseline survey as one cannot ascertain that the sexual behavior would be similar for comparison with data generated 2 years and 4 years later. This might introduce some bias in the interpretation of the results despite adjusted analysis. Young people's behavior is subject to changes over time. For this reason, the behavioral questionnaire addressing sexual behavior is an essential element of this study.

4.1.2.3 YEAR 2 IMPACT CROSS-SECTIONAL SURVEY

A similar cross-sectional survey will be performed at Year 2 post vaccination among female students from high schools and vocational schools. A representative subset of students of Grade 10 HS / year 1 VS would be eligible for the Year 2 impact survey regardless of their vaccination status. As students in vocational schools may have higher sexual risk behavior, a subset of Grade 10 HS will be randomly selected from each school, similar to proportion of schoolgirls per school in baseline survey, but intentionally sampled to over-represent vocational schools.

The assessment of the impact of vaccination in each province will be the reduction of HPV prevalence at Year 2 (vaccinees reach Grade 10 HS / year 1 VS) relative to the unvaccinated Grade 10 HS/ year 1 VS determined from the baseline cross-sectional survey. The demographic and behavioral data collected at each assessment will allow for the assessment of reported sexual activity by secondary Grade/vocational between participants enrolled in the baseline survey (collected end of 2018 or early 2019 and those reported at the impact assessments 2 years (2020) later.

- Students of Grade 10 HS / year 1 VS will be enrolled after obtaining informed parent consent for blood collection and student consents/assents to perform questionnaire, urine collection and blood collection.
- Demographic and behavioral data will be collected from all enrolled participants.
- Urine sample will be collected at school for HPV DNA PCR and genotyping from all participants.
- A blood sample will be collected at the District Hospital from a subset of those enrolled and vaccinated when they were Grade 8 (N=200 per province).
- The number enrolled for this survey will be N=2600 per province, for a total of N=5200.

4.1.2.4 YEAR 4 IMPACT CROSS-SECTIONAL SURVEY

Cross-sectional surveys will be performed at Year 4 among female students in secondary and vocational schools. A subset (N=2000) of representative female students of Grade 12 HS/ year 3 VS would be eligible if they have received HPV vaccine at Grade 8 according to the protocol, i.e., 1 dose for Udon Thani, and 2-dose for Buriram. As students in vocational schools may have higher sexual risk behavior, a subset of Grade 12 HS/ year 3 VS will be randomly selected from each school similar to proportion of schoolgirls per school in baseline survey, but intentionally sampled to over-represent vocational schools.

The assessment of the impact of vaccine in each province will be the reduction in HPV prevalence at Year 4 (vaccinees reach Grade 12 HS/ year 3 VS) relative to the non-vaccinated Grade 12 HS/ year 3 VS determined in the baseline cross-sectional survey. The demographic and behavioral data collected at each assessment will allow for the assessment of reported sexual activity by Grade/vocational between participants enrolled in the baseline survey (collected end of 2018 or early 2019 and those reported in impact assessments 4 years (2022) later.

- Students of Grade 12 HS/ year 3 VS will be enrolled after obtaining parent consent for blood collection and students consent/assents to perform questionnaire, urine collection and blood collection.
- Demographic and behavioral data will be collected from all enrolled participants.
- Urine sample will be collected at school for HPV DNA PCR and genotyping from all participants who were vaccinated at Grade 8.
- A blood sample will be collected at the District Hospital from a small subset of those enrolled and vaccinated at Grade 8 (N=200 per province).
- The number enrolled for this survey will be N=2000 per province, for a total of N=4000.

4.2 STUDY ENDPOINTS

4.2.1 PRIMARY ENDPOINT

- HPV 16 and HPV 18 DNA prevalence as measured in urine by DNA PCR at Year 2 and Year 4 post vaccination compared to unvaccinated female students

The assessment of the vaccination impact in each province will be the reduction in HPV prevalence at Year 2 and Year 4 post vaccination relative to the prevalence in non-vaccinated same Grade female students determined through the baseline cross-sectional survey. HPV infection will be assessed in Grade 10/year 1 and Grade 12/year 3 female student participants in the baseline cross-sectional survey and Year 2 (Grade 10/year 1) and Year 4 (Grade 12/year 3) cross-sectional surveys. There will be no such assessment in Grade 8 female students because the estimated prevalence would highly likely be very low.

4.2.2 SECONDARY ENPOINTS

The secondary endpoints of the study include:

- HPV 16 and 18 infection prevalence in Grades 10/year 1 and 12/year 3 female students at baseline survey, and Year 2 and Year 4 surveys post vaccination
- Distribution of HPV genotypes in HPV infections detected during the surveys. This will contribute to understand:

    - the possible cross-protection of vaccination against other HPV genotypes not targeted by the vaccine
    - to document the evolution of the frequency of circulating HPV genotypes over time and their relationship with sexual behavior
- HPV type-specific antibody response in a subset of participants prior to vaccination and at Year 2 and Year 4 post vaccination to ensure and document that the vaccine used in this study is immunogenic. The study is not powered to correlate vaccine-induced responses with effectiveness.

4.2.3 EXPLORATORY ENPOINTS

- Sexual behavior of female students from Grades 8, Grade 10 HS/year 1 VS and Grade 12 HS/year 3 VS. This will allow to compare the level of risk between provinces and between vaccinated and unvaccinated (baseline) female students at different time points.
- Comparability of risk (sexual behavior) for HPV infection (HPV prevalence) between Grades 10 HS and year 1 VS and Grade 12 HS and year 3 VS female students at baseline survey and at Year 2 and Year 4 cross-sectional surveys post vaccination
- Possible herd protection conferred by the HPV vaccination as indirect benefit of the vaccine in unvaccinated female students in each province in Year 2 survey

5 SCHOOL AND POPULATION SELECTION PROCEDURES

The target population for the vaccination is represented by all Grade 8 female students in the two provinces.

Target populations of surveys (baseline, Year 2 and Year 4 impact surveys) are a subset of female students at Grade 10 HS/year 1 VS and Grade 12 HS/year 3 VS from all schools in the two provinces and the sampling unit is an individual.

A baseline blood sample will be collected at the District Hospital from a randomly selected subset among those enrolled prior to vaccination (N=200 per province). Another subset of students (N=200 per province) will be randomly selected for each of the Year 2 and Year 4 surveys for blood collection for assessment of vaccine immunogenicity.

From the list of all schools in the two provinces, the expected enrollment number of participants of Grade 8, Grade 10 HS/year 1 VS and Grade 12 HS/year 3 VS from each school will be determined relative to the total number of female students in each school in each province.

Due to an expected higher sexual activity among students in vocational schools, the number of participants will be oversampled from vocational schools compared to high schools. Within each school, the planned number of survey participants will be enrolled once parent's consent and participant's assent have been obtained (See Section 7.2).

6 HPV VACCINE TO BE USED IN THE STUDY

6.1 ACQUISITION

CERVARIX HPV vaccine to be used in this study will be purchased from Glaxo Smith Kline (GSK, Rixensart, Belgium) by IVI. Vaccines will be transported and delivered directly to the district hospitals of Udon Thani and Buriram provinces. The transportation and delivery will be done under the responsibility of GSK Thailand.

6.1.1 HPV VACCINE CHARACTERISTICS, USE AND STORAGE

CERVARIX is manufactured by GSK. It is a bivalent HPV vaccine presented as a suspension for intramuscular injection containing purified viral L1 protein for HPV types 16 and 18. It is available in 1-dose or 2-dose vials or prefilled syringes. It is produced using a baculovirus expression system in Trichoplusia ni cells. Each 0.5 mL dose of the bivalent vaccine contains 20µg of HPV-16 L1 protein and 20 µg of HPV-18 L1 protein adsorbed onto a proprietary adjuvant system containing 500 µg of aluminum hydroxide and 50 µg of 3-O-desacyl-4-monophosphoryl lipid A (AS04).

This vaccine is indicated for use in females and males from the age of 9 years for the prevention of premalignant anogenital lesions affecting the cervix, vulva, vagina and anus, and cervical and anal cancers causally related to specific HPV types [21,64]. It has been tested in randomized

clinical trials in Thailand and shown to be safe and immunogenic [32,33]. No serious adverse reactions have been recorded since the nationwide implementation of the 2D-regimen (MOPH/DDC Epidemiology Department, Thailand).

CERVARIX should be maintained at +2–8 °C, not frozen, and administered as soon as possible after being removed from the refrigerator. However, stability has been demonstrated when stored outside the refrigerator for up to 3 days at temperatures between +8 °C and +25 °C, or for up to 1 day at temperatures between +25 °C and +37 °C.

In this study, CERVARIX will be administered intramuscularly in the deltoid of the non-dominant arm (usually left) either as single dose (SD) (Udon Thani Province) or as two-dose (2D 6 months apart) (Buriram Province) regimens.

6.1.2 VACCINE ACCOUNTABILITY

CERVARIX vaccine accountability will be under the responsibility of the PI or designee. Tracking procedures with recording documents will be put in place at District Hospitals (vaccine doses received, used, remaining at the end of the Grade 8 vaccination campaign) and detailed in the Manual of Operations.

7 STUDY POPULATION

7.1 STRATEGIES FOR RECRUITMENT

For baseline survey, vaccination, and cross-sectional surveys at Year 2 and Year 4 post vaccination, female students will be recruited from secondary school for Grade 8, high schools for Grade 10 and Grade 12 and from vocational shool year 1 and year 3 in each province. An exhaustive list of schools per province and district will be established and for each school, the number of female students for the grades considered. After consent and assent are obtained, students will be enrolled until the total number of participants needed for a given school is filled.

All female students from Grade 8 whose parents consented and who gave their assent will be offered either SD of 2D vaccination according to the province. A randomly selected subset of the vaccinated students will be asked to fill in the sexual behavioral questionnaire (N=1500).

For baseline, Year 2 and Year 4 surveys, we will provide the number of Grade 10 / year 1 (N=2600) and Grade 12 / year 3 (N=2000) to be selected, similar to proportion of enrolled schoolgirls per school in baseline survey. We will give overweight to vocational school so that 50% of students would be selected from vocational schools.

Blood collection will be performed in a subset of Grade 8 female students (N=200 per province) prior to vaccination and another subset of students (N=200 per province) at Year 2 and another subset at Year 4 (N=200 per province) post vaccination. For practicality purpose, students will be enrolled from schools who are closer to district hospitals. (SC: under Health Promoting Hospital)

7.2 CONSENT PROCEDURES AND DOCUMENTATION

As per principles of ICH GCP (section 2.0, ICH E6 (R2)) and ICH E11, parents or guardian and participants will be asked to provide informed consent and assent, respectively. Information sheets and informed consents and assents will be given to paticipants at school to be reviewed at home with parents. Specific information sheets and informed consents and assents will be obtained at school for the following study events as shown in **Table 6** below.

Table 6. Information sheet, parent consent and participant assent

	Information sheet	Parent consent	Participant Consent/assent
**Vaccination**			
Vaccination	X	X	X
Questionnaire	X		X
Blood	X	X	X
**Baseline survey**			
Questionnaire	X		X
Urine	X		X
**Year 2 post vaccination survey**			
Questionnaire and Urine	X		X
Blood	X	X	X

**Year 4 post vaccination survey**			
Questionnaire and Urine	X		X
Blood	X	X	X

7.3 COMPENSATION FOR PARTICIPATION

All female students will be compensated for their participation for each visit of the study events (baseline survey, vaccination, Year 2 and Year 4 post vaccination cross-sectional surveys) with tokens or snacks corresponding to the equivalent of 100 Thai Bahts as specified in the consent and assent forms.

7.4 PARTICIPANT INCLUSION AND EXCLUSION CRITERIA

The participant inclusion and exclusion criteria for each of the study components are shown in **Table 7** below.

Table 7. Participant inclusion and exclusion criteria for the four study components

	**Inclusion criteria**	**Exclusion criteria**
**Grade 8 (Mathayom 2)**	• Female students with identification card<br>• Less than 15 years of age<br>• Parent or guardian consent for vaccination and blood collection as applicable<br>• Participant assent for vaccination, questionnaire, and blood collection as applicable (**Table 6**)	• Students who already received HPV vaccination<br>• Reported pregnancy*<br>• Any student who has a pre-existing known medical condition or diagnosed psychological illness which in the opinion of the Principal Investigator or designee may be detrimental to her well-being**
**Baseline survey**<br><br>Grade 10 / year 1<br>Grade 12 / year 3	• Female students with identification card<br>• Participant assent for questionnaire and urine collection (**Table 6**)	• Any student who has a pre-existing known medical condition or diagnosed psychological illness which in the opinion of the Principal Investigator or designee may be detrimental to her well-being***

**Year 2 post vaccination survey**<br><br>Grade 10 / year 1<br><br>**Year 4 post vaccination survey**<br><br>Grade 12 / year 3	• Female students with identification card<br>• Parent or guardian consent for blood collection as applicable<br>• Participant consent/assent for questionnaire, urine and blood collection from those vaccinated at Grade 8 as applicable (**Table 6**)<br>• Received assigned dose regimen of HPV vaccine at Grade 8; 1 dose for Udon Thani, 2-dose for Buriram (Only for Year 4 post vaccination survey)	• Any student who has a pre-existing known medical condition or diagnosed psychological illness which in the opinion of the Principal Investigator or designee may be detrimental to her well-being***<br>• Self-reported HPV vaccination (self-purchased)<br>• Received only 1 dose or 3 doses of vaccine (for Buriram only)<br>• Received 2 doses or 3 doses of vaccine (For Udon Thani only)<br>• Received no HPV vaccine (For blood collection)

* For medical practice in Thailand, last menstrual period will be asked to ensure student is not pregnant. Performing a pregnancy test in school students would become a very sensitive issue in the Thai cultural context. This is the reason why we all agreed that only 'reported' pregnancy would be considered as an exclusion criterion in this study for vaccination.

Pregnancy in female students less than 15 years of age is a very rare event: 1.4/1000 in Udon Thani and 1.6/1000 in Buriram, similar to the national figure of 1.4/1000. If the vaccine were accidentally administered to a pregnant woman, the pregnant women would be followed up until delivery by the study staff. Pregnancy outcome will be reported to the MOPH Ethics Committee and IRBs.

** Known anaphylaxis to the components of the HPV vaccine, allergic reaction to the first injection that may preclude a second injection,fever, symptomatic infectious disease such as urinary tract infection, lower respiratory tract infection, known blood discrasy, major psychosis, Down syndrome (some being scholarized) for behavioral questionnaire

*** Symptomatic urinary tract infection, known blood discrasy, major psychosis, Down syndrome (some being scholarized) for behavioral questionnaire

7.5 STUDY PROCEDURES

Details of visit procedures during enrollment for each of the 4 separate study components are shown in the Schedule of Events in **Table 8** below.

Table 8. Schedule of Events

**Year 1 Per province**	**First Vaccination**	**Second Vaccination *Buriram only**	**Blood Collection**	**Behavioral Questionnaire**	**Urine Collection**
Parent/ Guardian Consent	Yes	NA	Yes	NA	NA

Student Assent	Yes	NA	Yes	Yes	Yes
Grade 8	N~8-9000	N~8-9000*	N=200	N=1500	NA
Grade 10/year 1	NA		NA	N=2600	
Grade 12/year 3	NA		NA	N=2000	

**Year 2 Per province**	**Blood Collection**	**Behavioral Questionnaire**	**Urine Collection**
Parent/ Guardian Consent	Yes	NA	
Student Consent/Assent	Yes	Yes	Yes
Grade 10 / year 1	N=200	N=2600	

**Year 4 Per province**	**Blood Collection**	**Behavioral Questionnaire**	**Urine Collection**
Parent/Guardian Consent	Yes	NA	
Student Consent/Assent	Yes	Yes	Yes
Grade 12 / year 3	N=200	N=2000	N=2000

7.6 PARTICIPANT WITHDRAWAL FOR FURTHER VACCINATION

7.6.1 REASONS FOR WITHDRAWAL FOR FURTHER VACCINATION

Parents/guardian and/or participants are free to withdraw their consent or assent from participation in the study at any time upon request, without justification and without prejudice. The Principal Investigator may also decide to discontinue further vaccination or study intervention(s) in the following cases:

- Pregnancy after first dose : no administration of second dose (Buriram). However, if pregnancy would occur after the first dose but before the second dose, the second dose will be offered after delivery.
- Occurrence of medical and/or psycho-social condition that in the judgment of the investigator may be detrimental for the participant's well-being
- Any other reason of study discontinuation as per the judgment of the Principal Investigator

7.6.2 HANDLING OF PARTICIPANT DISCONTINUATION

Discontinuation from study component does not preclude the participation in further components of the study (for example, if a participant refuses vaccination this participant could still be included in Year 2 or Year 4 surveys.) Similarly, a participant who refuses blood draw could still proceed with urine collection, as per study protocol. This will be recorded in the Data Collection Form (DCF).

7.7 PROTOCOL DEVIATIONS

A protocol deviation is any noncompliance with the study protocol or principles of ICH GCP (section 2.0, ICH E6 (R2)) by the participant, the investigator, or the study site staff. It is the responsibility of the PI to use continuous vigilance to identify and report all protocol deviations to MOPH EC and IVI IRB as per guidelines and to sponsor. The PI is responsible for knowing and adhering to the site requirements. The IVI study monitor will report all protocol deviations to the IVI PI and IVI IRB.

Major deviations are defined as those jeopardise the safety or rights of the participant or the scientific integrity of the study which is applicable to cases listed below:

- Violation of inclusion and exclusion criteria
- Vaccination with wrong vaccine as defined in the protocol
- Vaccination not following the immunization schedule defined in protocol
- Visit outside window for the immunogenicity assessment after discussion with the study medical monitor.
- Missed urine samples for HPV DNA PCR and blood samples for immunogenicity assessments.

Major protocol deviations thought to affect the scientific integrity of the study and/or the safety and rights of the participant will be reported and discussed with investigator, monitor, sponsor, and statistician for their exclusion from the analysis. For minor protocol deviations considered not to affect the scientific integrity of the study, the extent of deviation or delay as well as reason will be accurately documented.

7.8 PROTOCOL AMENDMENTS

Any amendment of the approved protocol shall be submitted to all MOPH EC, Chulalongkorn and IVI IRBs for approval. If the amendment relates to interventions with participants, information sheet, parent consent and participant consent/assent may have to be modified accordingly and resubmitted to MOPH EC, Chulalongkorn and IVI IRBs for approval.

7.9 PREMATURE TERMINATION OR SUSPENSION OF STUDY

This study may be temporarily suspended or prematurely terminated if there is sufficient reasonable cause as per PI recommendation after consultation with sponsor. MOPH EC, Chulalongkorn and IVI IRBs may also require termination of the study. Written notification, documenting the reason for study suspension or termination, will be provided by the suspending or terminating party to investigator, the sponsor, and EC/IRBs.

Circumstances that may warrant termination or suspension are:

- Determination of unexpected, significant, or unacceptable risk to participant as recommended by the PI
- Poor protocol compliance

Study may resume once concerns about safety, protocol compliance, data quality are addressed, resolved, and meeting the requirements from the sponsor and EC/IRBs.

7.10 END OF STUDY

The study will be considered completed once the Year 4 cross-sectional survey, all lab analyses and the final statistical analysis and study report will have been performed.

8 LABORATORY PROCEDURES AND EVALUATIONS

8.1 ASSESSMENT OF HPV INFECTION

Urine samples will be collected using a commercially available clean, standard urine collection device containing fixed volume of preservative and designed to collect standard volume of urine, will be pre labelled and provided to the participant. Participants will be instructed to collect the sample as per recommendation of the manufacturer of urine collection device and bring the sample to a pre designated area. First void urine sample collected any time during the day will be accepted. Samples from menstruating female students will be excluded or an option will be given to the participants to submit urine after the end of the cycle if she is willing to participate in the study. Such collection method has been successfully used in Bhutan and Rwanda for HPV prevalence assessments [65-70].

Urine samples will be collected at school and stored at 2-8°C within 30 minutes of collection. A constant temperature of 2-8°C will be maintained using a cold chain and freezing of the sample will be avoided before processing. The sample will be transported and stored at the Disctrict and Provincial Hospitals before shipment to Chulalongkorn lab where it will be further processed within 48 hours of reception. An aliquot of urine will be centrifuged. The cell pellet thus obtained will be suspended in preservative solution and frozen at -20°C till further testing for detection of HPV DNA and genotyping as explained in the section below. A second aliquot of urine will be frozen at -20°C (-70°C) for further testing or re-testing if required or till the end of the study period.

HPV infection will be determined in urine samples by PCR DNA assay as described earlier by the Chulalongkorn lab [71,72]. In this study, a high throughput, automated, commercially available, validated qualitative assay utilizing amplification of target DNA by PCR and nucleic acid hybridization for detection of HPV type 16 and 18 and at least 12 other types of HPV in urine samples will be used for the HPV DNA detection assay [73-75]. Positive samples will then be genotyped [76]. All samples will be bar-code labeled using a unique alpha numeric study ID. The date of collection to be hand written on the sample.

An internal quality control and quality assurance plan will be implemented and the laboratory will participate in external quality proficiency testing program to ensure consistent and reliable test results.

8.2 ASSESSMENT OF IMMUNOGENICITY

An HPV type-specific antibody assessment will be performed in a subset (N=200 per province) of vaccinated female student for each of the groups: Grade 8 prior vaccination, Grade 10/year 1 at Year 2 and Grade 12/year 3 at Year 4 post vaccination. The purpose of this assessment is mostly to document the immunogenicity of the vaccine used in this study. A correlation with effectiveness would not be powered with this small sample size.

Blood will be collected by an experienced health care worker or phlebotomist under aseptic condition and following standard universal precautions. Using disposable syringe and needle, a maximum of 3 mL blood will be collected from peripheral vein and transferred into a pre labelled plain vial. The blood will be allowed to clot for 30 minutes at room temperature and centrifuged to separate the clot and serum. The serum obtained will be divided into two aliquots. Each aliquot should contain at least 0.5 mL of serum. Sera will be stored at -20°C at the Provincial Hospital and transported to the lab at Chulalongkorn University maintaining cold chain using dry ice. One aliquot will be sent to US Centre for Disease Control (CDC), USA, under Material Transfer Agreement (MTA) for anlaysis as described below. The second aliquot will be stored at Chulalongkorn University laboratory at -70°C for future confirmatory analysis. If in case the desired volume of serum sample will not be available, any amount of serum hence collected will be stored and prioritized for the immunoassay to be carried out at the US CDC.

The purpose of the immunogenicity study is to verify that the vaccine administered is indeed immunogenic for the vaccine HPV 16 and 18 genotypes and to assess immunogenicity to non-vaccine genotypes (HPV 6, 11, 31, 33, 45, 52, 58) reflecting sexual exposure and potential cross-immunogenicity. It does not intend to correlate immunogenicity with effectiveness.

Antibody titers will be determined at the US CDC laboratory using multiplex direct IgG ELISA against L1/L2 HPV virus-like particles (VLPs) on the Meso Scale Discovery (MSD) platform with chemiluminescent detection as described with minor modifications [77]. Briefly, conformationally intact VLPs are coated on 7-spot/well MSD plates. Serial 3.16 fold dilutions of standards, controls and test sera will be prepared and at least 3 dilutions starting at 1:100 or higher will be assayed. Following blocking, serum binding and washing, bound IgG is detected with optimized Sulfo-tag labeled mouse anti-human total IgG (Fc specific) (Biotrend-MSD) and signals read on MSD imager. Phase-Locked Loop (PLL) analysis will be performed as described in the WHO HPV Labnet Manual, using raw signal for each HPV type. The PLL value is the antibody titer of the test

serum relative to the standard/reference serum used [78]. Cut-off values (COV) will be determined using serum samples from Children (n=50). Test samples will be considered positive if they passed PLL conditions as well as were above Median+ 2 Standard Deviations of the PLL/titer generated from the children sera. Results for HPV 16 and 18 will be reported in International Units/mL. HPV 6 and 11 will be reported in Arbitrary Units/mL until International Standards are available.

If the student refuse the blood collection, it will not affect her participation in other parts of the study (vaccination, urine collection, behavioral survey).

9 SAFETY MONITORING

This is a community intervention effectiveness study of one or two doses of CERVARIX, a licensed vaccine approved by the Thai FDA since 2012. Numerous safety studies have already been conducted including in Thailand [32,79-81]. Since the study involves a large number of female students who will be vaccinated (N=16,000-18,000), the safety events experienced by the vaccinated student will be reported to the study site staff and transmitted to the Principal Investigator for review and then entered into the database.

An Adverse Event Following Immunization (AEFI) is any untoward medical occurrence which follows immunization and which does not necessarily have a causal relationship with the administration of the vaccine. As per current practice in the national surveillance system, the AEFI listed below which occurred within 4 weeks after vaccination should be reported:

1. Result in death with unknown cause
2. Neurological syndrome such as seizures, muscle weakness, encephalitis, etc.
3. Severe allergic reaction such as anaphylaxis
4. Sepsis
5. High fever with redness and swelling at the injection site for more than 3 days.
6. Any events probably related to the vaccination
    - d. Hospitalization
    - e. Cluster of students with same events
    - f. Non severe symptoms such as rash and abcess at the injection site.

The PI, sub-investigators, and site staff will exercise due diligence in ascertaining, accurately recording all reportable AEFI that vaccinated study participants may have experienced. Reported AEFI will be linked to the study ID of the students.

Site investigators will contact the PI and ask for appropriate management as required. AEFI will be submitted to the Bureau of Epidemiology of the Department of Disease Control, MOPH, who will review all AEFI submitted by the PI. AEFI may also be reported to the MOPH EC as per local regulations.

10 STUDY MONITORING

Study monitoring will be under the joint responsibility of the Sponsor and MOPH/DDC (co-monitoring). The study staff will follow site-specific manual of procedures (MOP) to ensure effective protocol implementation, compliance with applicable regulatory requirements, to identify areas that need corrective action, verify data accuracy. Monitors may review individual participant study records to ensure protection of study participants, compliance with the protocol, and accuracy and completeness of records. This may also include inspection of the regulatory files to ensure that regulatory requirements are being followed.

Study progress will be monitored by the IVI study team jointly with MOPH and Chulalongkorn Lab representatives as frequently as necessary to ensure the rights and well-being of study participants are protected; to verify adequate, accurate and complete data collection; protocol compliance and to determine that the study is being conducted in conformity with applicable regulatory requirements. Arrangements for monitoring visits will be made sufficiently in advance in accordance with the monitoring plan.

11 DATA MANAGEMENT AND STATISTICAL CONSIDERATIONS

11.1 STATISTICAL HYPOTHESIS AND SAMPLE SIZE

11.1.1 VACCINATION

The total sample size of Grade 8 participants (N=16,000-18,000) to receive HPV vaccination is based on the current estimate of Grade 8 students in secondary schools of Udon Thani and Buriram provinces. All willing Grade 8 students would be vaccinated in these two provinces.

11.1.2 BASELINE SURVEY AND YEAR 2 AND YEAR 4 SURVEYS

Assuming a 90% vaccine coverage of Grade 8 participants, the sample size of N=2600 per province at Year 2, and N=2000 per province at Year 4 are calculated to provide >80% power to

show the vaccine effectiveness (VE) of SD is non-inferior to VE of 2D with non-inferiority margin of 10% using one-sided test at 0.025 significance level. In addition, this sample size is calculated to provide 80% power to test the vaccine effectiveness of SD or 2D is greater than 50% using one-sided test at a 0.025 significance level and 90% vaccination coverage. The assumed prevalence of HPV 16 and or 18 in Grade 10/year 1 and Grade 12/year 3 female students in the two provinces (for Year 2 and Year 4 year impact surveys, respectively) are 2% and 3% respectively. Due to lack of information on HPV prevalence in female students in Thailand, the prevalence of HPV infection in Grade 10/year 1 and Grade 12/year 3 will be estimated based on baseline survey.

11.1.3 BEHAVIORAL QUESTIONNAIRE

The sample size for the behavioral questionnaire N=1500 is based on the current estimate of the rate of sexual risk (experience of sexual activity) as 2% [95% CI 1.4%-2.8%] and for which we expect to see about 30 sexually active Grade 8 female students per region.

For Grade 10/year 1 and Grade 12/year 3 baseline and Year 2 and Year 4 surveys, all female students enrolled in these events would be asked to fill in the questionnaire in order to link risk behavior with HPV prevalence (N=2600 for Grade 10 high school / year 1 vocational school and N=2000 for Grade 12 high school /year 3 vocational school per province).

Education to prevent recurrence of behavioral risks emerging from review of the questionnaires will be addressed. This is the normal practice and responsibility of MOPH staff to provide adequate and appropriate counseling to students. Staff will be trained and supervised by senior MOPH staff to ensure that those concerns are delivered to all students participating in the study.

11.1.4 BLOOD COLLECTION

For the assessment of HPV type-specific antibody response to the vaccine, the sample size N=200 per province is based on keeping the CV of mean titer of immunogenicity (HPV-16 or HPV 18) for SD or 2D as less than 2.5, so that the 95% CI of GMT estimate of SD or 2D can be comparable to the estimate of GMT from other studies in similar populations in Thailand [32]. The MOPH site staff will randomly select the assigned number of students from different schools in order to complete the sample size of N=200.

11.2 DESCRIPTION OF STATISTICAL METHODS

11.2.1 GENERAL APPROACH

The target population is composed of female students from all schools in Udon Thani and Buriram provinces and sampling unit is the student. We will perform random sampling including oversampling from vocational schools (50%) and regular schools (50%) and proportional sampling by number of students in each school.

The statistical analysis will focus on accurate estimation of HPV infection prevalence, vaccine coverage in Grades 10/year 1 and 12/year 3 female students. Sexual behavior questionnaire information will be descriptive to assess comparability of Grades 10/year 1 and Grade 12/year 3 at Year 2 and Year 4 impact surveys with those at baseline survey.

11.2.2 ANALYSIS OF PRIMARY ENDPOINT

The prevalence of HPV infection will be measured by DNA PCR in urine samples.

- To estimate the vaccine effectiveness (VE) of SD and 2D, the percent reduction of HPV 16 and 18 prevalence at the Year 2 and Year 4 impact surveys from the prevalence of Grade 10 / year 1 and Grade 12 / year 3 at the baseline cross-sectional survey will be calculated
- To establish whether the VE of SD is comparable to 2D, VE estimates at Year 4 impact survey in SD province will be tested to show non-inferiority to the estimate of VE in 2D province. Using the baseline information of two provinces, any risk factors for HPV infection such as whether the participant is from vocational school, participant is sexually active, may be considered as adjustment in the comparison.

11.2.3 ANALYSIS OF SECONDARY ENDPOINTS

The frequency of the different HPV genotypes detected in positive urine samples will be summarized for each assessment (baseline and Year 2 and Year 4 impact survey) in each province.

As secondary endpoint, immunogenicity assessment will be performed in a subgroup at the time of immunization and at Year 2 and Year 4 impact assessments (N=200 per province at each assessment). Geometric mean titer (GMT) of serum ELISA antibody at Year 2 and Year 4 impact assessment after SD or 2D will be summarized.

11.2.4 EXPLORATORY ENDPOINTS

The responses to the sexual behavior questionnaire from Grade 8, Grade 10/year 1 and Grade 12/year 3 students at each assessment in each province will be summarized descriptively and utilized to assess the potential ascertainment bias for estimates due to survey sample selection and and subsequent comparison with Grade 10/year 1 and Grade 12/year 3 female students at baseline survey at each assessment. If any critical differences are found for some variables, an adjusted analysis will be performed as sensitivity analysis. If any critical differences are found for some variables, an adjusted analysis will be performed as sensitivity analysis.

Herd protection may be analyzed assuming that there would be sufficient unvaccinated female students at Year 2 cross-sectional survey to allow this analysis. HPV prevalence in unvaccinated students at Year 2 (Grade 10/year 1) would be compared to the HPV prevalence of same school grade unvaccinated students from the baseline survey.

11.2.5 CRITERIA FOR A SECOND DOSE IN UDON THANI FEMALE STUDENTS

The hypothesis of this study is that the effectiveness of a single dose of CERVARIX would be similar to two doses 2 years and 4 years after the vaccination of Grade 8 female students. There is strong evidence that this may be the case based on previous studies as reviewed in the introduction. However, we cannot ascertain this fact. For this reason, we have developed a set of criteria that would guide the decision of whether or not a second dose should be administered. It must be acknowledged that Grade 8 female students would receive no dose outside this study since there is no catch-up vaccination campaign planned (only Grade 5 students benefit of the roll-out HPV vaccination program.)

The first assessment of HPV infection would occur 2 years post vaccination. Criteria have therefore been developed based on this Year 2 cross-sectional survey where Grade 8 students would have reached Grade 10 high school or year 1 vocational school.

The conditions for considering the administration of a second dose of vaccine are described below:

- The estimated effectiveness of 2D after cross-sectional survey in Buriram must have a lower bound >50%.
- The estimated effectiveness of SD after Year 2 cross-sectional survey in Udon Thani must have an upper bound of <50%, AND
- Be inferior to 2D with a margin of >25% difference in vaccine effectiveness.

The basic assumption is that vaccine effectiveness less than 50% would be of little public health benefit. The following conditions derive from this first assumption. This justifies the first criterion: the 2D regimen must clearly demonstrate effectiveness >50% (which means that the lower bound of the confidence interval must be greater than 50%).

The second criterion is that the SD effectiveness must be clearly less than 50%. Therefore, the SD effectiveness upper bound of the confidence interval would need to be less than 50%. To ensure that there is a true difference in effectiveness, the two point estimates should be sufficiently different. Therefore, the third criterion is that the difference between the two estimates is >25%.

If none of the criteria developed above were met, the second dose would not be administered.

Data will be reviewed at Year 2 by an ad hoc expert committee to decide whether or not to administer a second dose of HPV vaccine in Udon Thani participants. The ad hoc expert committee will be composed of three experts independent from the study proposed by Thai MOPH DDC and IVI. The members would include a biostatistician, a vaccinologist and a public health expert.

Study staff will be responsible to contact the students who received the first dose according to the ID listings in the database and the corresponding list of names stored at the MOPH.

Should the continuation of the study with SD be granted (no administration of second dose), a similar review process will take place after the analysis at Year 4 to decide whether or not to administer a second dose of HPV vaccine in Udon Thani participants.

12 SOURCE DOCUMENTS AND ACCESS TO SOURCE DOCUMENTS

Data recorded on the Data Collection Form will be reviewed before data entry in order to ensure data completeness and accuracy as required by study protocol. The required study forms (parent informed consent, participant consent/assent and survey forms) will be stored at the office of MOPH in a secured place under lock and key. The investigator and/or site staff must make all study forms and source documents of participants enrolled in this study available for review by the study monitoring team at the time of each monitoring visit.

At a minimum, all collected informed consents, assents and study survey forms must be available to substantiate participant identification, eligibility and participation, proper informed consent procedures, dates of survey performed, adherence to protocol procedures, adequate reporting of AEFI (if any), study vaccine accountability records, study vaccine administration information, and date of completion. AEFI will be submitted to the Bureau of Epidemiology of the Department of Disease Control, MOPH, who will review all AEFI submitted by the PI. Specific items required as source documents will be reviewed with the investigator before the study.

The source documents must also be available for inspection, verification and copying, as required by regulations, and for possible audit by funding agency (e.g., BMGF). The investigator and study site staff must comply with applicable privacy and data protection for use and disclosure of information related to the study and enrolled participants.

Each participant will have a complete Data Collection Form (expect sexual activity questionnaire) of records including study log books, and ICF for the entire study period. Appropriate source documents will be prepared by study staffs. These records must be available to the IVI and regulatory authories upon request for review.

13 DATA HANDLING AND RECORD KEEPING

13.1 DATA COLLECTION AND MANAGEMENT RESPONSIBILITIES

The electronic web-based system for data management using paper and paper-less will be developed by IVI for the collection of participant-specific information including vaccination and demographic, sexual behaviour, and laboratory results. The survey data on sexual activity information will be captured directly from participants using individual smart phones or staffs tablets in order to protect participant privacy. Lab data will be electronically transferred to the IVI

main database system for data quality control. Vaccination information, demographics at baseline, Year 2 and Year 4 impact survey information will be collected on paper and will be entered in the web based system.

This data management (DM) system will include computer system functionality of entry, editing, browsing, downloading and uploading, providing error reports, exception lists and summary reports for each activity. In addition, audit trail of DM system would record all sequential changes made in the database. Data entry programs will incorporate identification of the range, consistency and duplication checks.

The database will be located and managed at the IVI Biostatistics and Data Management Department and copied to MOPH server regularly or can be downloaded by MOPH at anytime. Data security for this data management system will be reinforced by automatic computer virus scanning at start-up of each data entry and data management session, and password protection for accessing data and data management software. Regarding confidentiality of data, the access to database will be controlled by user ID and password depending on their role and responsibility of study team members and the personal identification information of participants will not be stored in IVI server. In addition, backup files generated by the data management system will be kept in a secure place. Data entry and cleaning will be conducted at the sites. Final data cleaning, data locking and data analysis will be performed at the IVI.

13.2 STUDY RECORDS RETENTION

The Principal Investigator will retain all study records required by sponsor and by the applicable regulations in a secure and safe facility. The PI will consult IVI representative before disposal of any study records, and will notify the sponsor of any change in the location, disposition, or custody of the study files. These documents should be retained at least 2 years after the end of the study (ICH-E6(R2), 4.9.5). IVI will inform the PI as to when these documents no longer need to be retained (ICH-E6(R2), 5.5.12).

13.3 PUBLICATION AND DATA SHARING POLICY

IVI assures that the key design elements of this protocol will be posted in a publicly accessible database at a minimum in a Thai registery. All data collected during this study may be used to guide HPV vaccination policy and WHO. All individual data will stay strictly confidential. Analyzed

data may be presented in scientific conferences, and published in peer-reviewed scientific journals. Anyone wishing to publish or present data obtained during and/or aftercompletion of the study will conform to MOPH, Chulalongkorn University and US CDC policies and then forward the publication for review and approval to IVI.

14 QUALITY ASSURANCE AND QUALITY CONTROL

Quality Assurance (QA) oversight will be required at all stages of the trial process guided by the principles of ICH E6 (R2 Section 2) and/or local government GCP requirements. Quality Control (QC) procedures will be implemented beginning with the data entry system and data QC checks. Any missing data or data anomalies will be communicated to the site(s) for clarification/resolution.

During study conduct, the Sponsor or its designee (e.g., CRO) will conduct periodic monitoring visits (i.e., QC checks) to ensure that the protocol, Good Clinical Practice, local regulatory requirements and sponsor’s controlled documents (e.g., Standard Operating Procedure) are being followed. The monitors will review source documents to confirm that the data recorded on CRFs/eCRFs are accurate.

In addition to on-going QA oversight, selected investigator sites will be subjected to quality assurance audits performed by the sponsor or its designee, and/or by inspection by regulatory authorities and/or notified bodies. The investigational sites will provide direct access to all study related sites, source data/documents, and reports for the purpose of monitoring and auditing by the sponsor; inspection by local and regulatory authorities and/or notified bodies.

15 ETHICS AND PROTECTION OF HUMAN SUBJECTS

15.1 REGULATORY AND ETHICAL COMPLIANCE

The study will be initiated after obtaining approval from the Ministry of Public Health Ethical Review Committee of Research in Human Subjects (MOPH EC) and from the IVI Institutional Review Board (IVI IRB) guided by the principles of ICH GCP (section 2.0, ICH E6 (R2)).

The PI will ensure that this study is conducted in guided by the principles of ICH GCP (section 2.0, ICH E6 (R2)), Council for International Organizations of Medical Science (CIOMS), local

country's Ethics policy statement or the Declaration of Helsinki, whichever provides the most protection to human subjects.

15.2 PARTICIPANT AND DATA CONFIDENTIALITY

Participant confidentiality is strictly held in trust by the participating investigators, laboratories involved, their staff, and the sponsor. This confidentiality is extended to cover testing of biological samples in addition to the general and sexual beahavior information relating to participants. Therefore, the study protocol, documentation, data, and all other information generated will be held in strict confidence. No information concerning the study or the data will be released to any unauthorized third party without prior written approval of the sponsor.

The following elements contribute to ensure sexual behavior data privacy: 1/ Student ID is unidentifiable; 2/ Student sexual behavior data is self-administered in the electronic system without personal identifier; 3/ Security of the data management system is a warrantee for the privacy or the information collected in this study.

The study monitor, other authorized representatives of the sponsor, representatives of the EC/IRB may inspect all documents and records required to be maintained by the investigators. The clinical study site will permit access to such records.

The study participant's contact information will be securely stored at study site for internal use during the study. At the end of the study, all records will continue to be kept in a secure location for as long a period as required by MOPH EC and IVI IRB and institutional regulations.

Individual participants and their research data will be identified by a unique study identification number. The study data entry and study management systems used by study sites and by IVI Data Management will be secured and password-protected.

No personal identifier will be used in any publication or communication used to support this research study. The participant's identification number will be used in the event it becomes necessary to identify data specific to a single participant.

15.3. RESEARCH USE OF HUMAN SAMPLES

- Intended Use: Samples and data collected under this protocol may be used to detect HPV infection (urine) and to study immune responses (blood) to the vaccines administered and for safety purpose if deemed necessary per medical judgement of the PI or special request from the sponsor or EC/IRBs. No genetic testing will be performed.
- Storage: Samples and data will be stored at study sites and Chulalongkorn Lab (urine) and US CDC Lab (blood) using codes assigned by the investigators and lab. Data will be kept in password-protected computers.
- Disposition at the completion of the study: All samples will be sent to Chulalongkorn Lab for long term storage under agreement between MOPH and Chulalongkorn Lab. Study participants who request destruction of samples will be notified of compliance with such request and all supporting details will be maintained for tracking.

15.4 USE OF STORED BIOLOGICAL SPECIMENS

With the parents or guardian and participant's approval (consent and assent forms) and as approved by PI and EC/IRBs, the identified blood samples will be stored for 5 years after the end of the study at the Chulalongkorn lab and US CDC lab for confirmatory studies. There will be no stored urine sample after the end of the study. For each participant, all samples will be bar code labelled to maintain the masking of the identity of the participant.

During the conduct of the study, an individual participant can choose to withdraw consent to have blood specimens stored for future research. However, withdrawal of consent with regard to sample storage will not be possible after the study is completed.

The stored blood samples may be used for repeat of assays specified in protocol, and for additional assessment including – but not limited to - immunogenicity, study of possible immune correlates of protection, validation of assays, and testing of new assays.

16 REFERENCES

1. Bruni L, Diaz M, Castellsague X, Ferrer E, Bosch FX, et al. (2010) Cervical human papillomavirus prevalence in 5 continents: meta-analysis of 1 million women with normal cytological findings. J Infect Dis 202: 1789-1799.
2. Smith JS, Melendy A, Rana RK, Pimenta JM (2008) Age-specific prevalence of infection with human papillomavirus in females: a global review. J Adolesc Health 43: S5-25, S25 e21-41.
3. Smith JS, Lindsay L, Hoots B, Keys J, Franceschi S, et al. (2007) Human papillomavirus type distribution in invasive cervical cancer and high-grade cervical lesions: a meta-analysis update. Int J Cancer 121: 621-632.
4. Insinga RP, Dasbach EJ, Elbasha EH, Liaw KL, Barr E (2007) Progression and regression of incident cervical HPV 6, 11, 16 and 18 infections in young women. Infect Agent Cancer 2: 15.
5. Bosch FX, Lorincz A, Munoz N, Meijer CJ, Shah KV (2002) The causal relation between human papillomavirus and cervical cancer. J Clin Pathol 55: 244-265.
6. de Martel C, Plummer M, Vignat J, Franceschi S (2017) Worldwide burden of cancer attributable to HPV by site, country and HPV type. Int J Cancer 141: 664-670.
7. de Sanjose S, Quint WG, Alemany L, Geraets DT, Klaustermeier JE, et al. (2010) Human papillomavirus genotype attribution in invasive cervical cancer: a retrospective cross-sectional worldwide study. Lancet Oncol 11: 1048-1056.
8. Serrano B, de Sanjose S, Tous S, Quiros B, Munoz N, et al. (2015) Human papillomavirus genotype attribution for HPVs 6, 11, 16, 18, 31, 33, 45, 52 and 58 in female anogenital lesions. Eur J Cancer 51: 1732-1741.
9. Greer CE, Wheeler CM, Ladner MB, Beutner K, Coyne MY, et al. (1995) Human papillomavirus (HPV) type distribution and serological response to HPV type 6 virus-like particles in patients with genital warts. J Clin Microbiol 33: 2058-2063.
10. Sturegard E, Johansson H, Ekstrom J, Hansson BG, Johnsson A, et al. (2013) Human papillomavirus typing in reporting of condyloma. Sex Transm Dis 40: 123-129.
11. Patel H, Wagner M, Singhal P, Kothari S (2013) Systematic review of the incidence and prevalence of genital warts. BMC Infect Dis 13: 39.
12. (2017) Human papillomavirus vaccines: WHO position paper, May 2017. Wkly Epidemiol Rec 92: 241-268.
13. Doorbar J, Quint W, Banks L, Bravo IG, Stoler M, et al. (2012) The biology and life-cycle of human papillomaviruses. Vaccine 30 Suppl 5: F55-70.

14. Denny LA, Franceschi S, de Sanjose S, Heard I, Moscicki AB, et al. (2012) Human papillomavirus, human immunodeficiency virus and immunosuppression. Vaccine 30 Suppl 5: F168-174.
15. Plummer M, de Martel C, Vignat J, Ferlay J, Bray F, et al. (2016) Global burden of cancers attributable to infections in 2012: a synthetic analysis. Lancet Glob Health 4: e609-616.
16. Larson DA, Derkay CS (2010) Epidemiology of recurrent respiratory papillomatosis. APMIS 118: 450-454.
17. Stanley MA (2012) Epithelial cell responses to infection with human papillomavirus. Clin Microbiol Rev 25: 215-222.
18. Giuliano AR, Lee JH, Fulp W, Villa LL, Lazcano E, et al. (2011) Incidence and clearance of genital human papillomavirus infection in men (HIM): a cohort study. Lancet 377: 932-940.
19. Stanley MA, Sterling JC (2014) Host responses to infection with human papillomavirus. Curr Probl Dermatol 45: 58-74.
20. World Health Organization. Electronic address swi (2017) Human papillomavirus vaccines: WHO position paper, May 2017-Recommendations. Vaccine 35: 5753-5755.
21. *http://www.ema.europa.eu/docs/en_GB/document_library/EPAR_Product_Information/human/000721/WC500024632.pdf*.
22. *http://monographs.iarc.fr/ENG/Monographs/vol90/mono90.pdf*.
23. Giannini SL, Hanon E, Moris P, Van Mechelen M, Morel S, et al. (2006) Enhanced humoral and memory B cellular immunity using HPV16/18 L1 VLP vaccine formulated with the MPL/aluminium salt combination (AS04) compared to aluminium salt only. Vaccine 24: 5937-5949.
24. Dauner JG, Pan Y, Hildesheim A, Harro C, Pinto LA (2010) Characterization of the HPV-specific memory B cell and systemic antibody responses in women receiving an unadjuvanted HPV16 L1 VLP vaccine. Vaccine 28: 5407-5413.
25. Lehtinen M, Paavonen J, Wheeler CM, Jaisamrarn U, Garland SM, et al. (2012) Overall efficacy of HPV-16/18 AS04-adjuvanted vaccine against grade 3 or greater cervical intraepithelial neoplasia: 4-year end-of-study analysis of the randomised, double-blind PATRICIA trial. Lancet Oncol 13: 89-99.
26. Hildesheim A, Wacholder S, Catteau G, Struyf F, Dubin G, et al. (2014) Efficacy of the HPV-16/18 vaccine: final according to protocol results from the blinded phase of the randomized Costa Rica HPV-16/18 vaccine trial. Vaccine 32: 5087-5097.

27. Lang Kuhs KA, Porras C, Schiller JT, Rodriguez AC, Schiffman M, et al. (2014) Effect of different human papillomavirus serological and DNA criteria on vaccine efficacy estimates. Am J Epidemiol 180: 599-607.
28. Einstein MH, Baron M, Levin MJ, Chatterjee A, Edwards RP, et al. (2009) Comparison of the immunogenicity and safety of Cervarix and Gardasil human papillomavirus (HPV) cervical cancer vaccines in healthy women aged 18-45 years. Hum Vaccin 5: 705-719.
29. Einstein MH, Takacs P, Chatterjee A, Sperling RS, Chakhtoura N, et al. (2014) Comparison of long-term immunogenicity and safety of human papillomavirus (HPV)-16/18 AS04-adjuvanted vaccine and HPV-6/11/16/18 vaccine in healthy women aged 18-45 years: end-of-study analysis of a Phase III randomized trial. Hum Vaccin Immunother 10: 3435-3445.
30. Dobson SR, McNeil S, Dionne M, Dawar M, Ogilvie G, et al. (2013) Immunogenicity of 2 doses of HPV vaccine in younger adolescents vs 3 doses in young women: a randomized clinical trial. JAMA 309: 1793-1802.
31. Romanowski B, Schwarz TF, Ferguson L, Peters K, Dionne M, et al. (2016) Sustained immunogenicity of the HPV-16/18 AS04-adjuvanted vaccine administered as a two-dose schedule in adolescent girls: Five-year clinical data and modeling predictions from a randomized study. Hum Vaccin Immunother 12: 20-29.
32. Puthanakit T, Huang LM, Chiu CH, Tang RB, Schwarz TF, et al. (2016) Randomized Open Trial Comparing 2-Dose Regimens of the Human Papillomavirus 16/18 AS04-Adjuvanted Vaccine in Girls Aged 9-14 Years Versus a 3-Dose Regimen in Women Aged 15-25 Years. J Infect Dis 214: 525-536.
33. Huang LM, Puthanakit T, Cheng-Hsun C, Ren-Bin T, Schwarz T, et al. (2017) Sustained Immunogenicity of 2-dose Human Papillomavirus 16/18 AS04-adjuvanted Vaccine Schedules in Girls Aged 9-14 Years: A Randomized Trial. J Infect Dis 215: 1711-1719.
34. https://clinicaltrials.gov/ct2/show/NCT01984697.
35. Lazcano-Ponce E, Stanley M, Munoz N, Torres L, Cruz-Valdez A, et al. (2014) Overcoming barriers to HPV vaccination: non-inferiority of antibody response to human papillomavirus 16/18 vaccine in adolescents vaccinated with a two-dose vs. a three-dose schedule at 21 months. Vaccine 32: 725-732.
36. Hernandez-Avila M, Torres-Ibarra L, Stanley M, Salmeron J, Cruz-Valdez A, et al. (2016) Evaluation of the immunogenicity of the quadrivalent HPV vaccine using 2 versus 3 doses at month 21: An epidemiological surveillance mechanism for alternate vaccination schemes. Hum Vaccin Immunother 12: 30-38.

37. Kreimer AR, Struyf F, Del Rosario-Raymundo MR, Hildesheim A, Skinner SR, et al. (2015) Efficacy of fewer than three doses of an HPV-16/18 AS04-adjuvanted vaccine: combined analysis of data from the Costa Rica Vaccine and PATRICIA Trials. Lancet Oncol 16: 775-786.
38. Basu P, Bhatla N, Ngoma T, Sankaranarayanan R (2016) Less than 3 doses of the HPV vaccine - Review of efficacy against virological and disease end points. Hum Vaccin Immunother 12: 1394-1402.
39. Sankaranarayanan R, Prabhu PR, Pawlita M, Gheit T, Bhatla N, et al. (2016) Immunogenicity and HPV infection after one, two, and three doses of quadrivalent HPV vaccine in girls in India: a multicentre prospective cohort study. Lancet Oncol 17: 67-77.
40. Drolet M, Benard E, Boily MC, Ali H, Baandrup L, et al. (2015) Population-level impact and herd effects following human papillomavirus vaccination programmes: a systematic review and meta-analysis. Lancet Infect Dis 15: 565-580.
41. Malagon T, Drolet M, Boily MC, Franco EL, Jit M, et al. (2012) Cross-protective efficacy of two human papillomavirus vaccines: a systematic review and meta-analysis. Lancet Infect Dis 12: 781-789.
42. Kemp TJ, Hildesheim A, Safaeian M, Dauner JG, Pan Y, et al. (2011) HPV16/18 L1 VLP vaccine induces cross-neutralizing antibodies that may mediate cross-protection. Vaccine 29: 2011-2014.
43. Draper E, Bissett SL, Howell-Jones R, Waight P, Soldan K, et al. (2013) A randomized, observer-blinded immunogenicity trial of Cervarix((R)) and Gardasil((R)) Human Papillomavirus vaccines in 12-15 year old girls. PLoS One 8: e61825.
44. Mesher D, Soldan K, Lehtinen M, Beddows S, Brisson M, et al. (2016) Population-Level Effects of Human Papillomavirus Vaccination Programs on Infections with Nonvaccine Genotypes. Emerg Infect Dis 22: 1732-1740.
45. Mesher D, Panwar K, Thomas SL, Edmundson C, Choi YH, et al. (2018) The Impact of the National HPV Vaccination Program in England Using the Bivalent HPV Vaccine: Surveillance of Type-Specific HPV in Young Females, 2010-2016. J Infect Dis.
46. Kavanagh K, Pollock KG, Cuschieri K, Palmer T, Cameron RL, et al. (2017) Changes in the prevalence of human papillomavirus following a national bivalent human papillomavirus vaccination programme in Scotland: a 7-year cross-sectional study. Lancet Infect Dis 17: 1293-1302.
47. Naud PS, Roteli-Martins CM, De Carvalho NS, Teixeira JC, de Borba PC, et al. (2014) Sustained efficacy, immunogenicity, and safety of the HPV-16/18 AS04-adjuvanted vaccine:

final analysis of a long-term follow-up study up to 9.4 years post-vaccination. Hum Vaccin Immunother 10: 2147-2162.

48. *http://www.fda.gov/BiologicsBloodVaccines/SafetyAvailability/VaccineSafety/ucm376465.htm*.

49. Paavonen J, Naud P, Salmeron J, Wheeler CM, Chow SN, et al. (2009) Efficacy of human papillomavirus (HPV)-16/18 AS04-adjuvanted vaccine against cervical infection and precancer caused by oncogenic HPV types (PATRICIA): final analysis of a double-blind, randomised study in young women. Lancet 374: 301-314.

50. Safaeian M, Porras C, Pan Y, Kreimer A, Schiller JT, et al. (2013) Durable antibody responses following one dose of the bivalent human papillomavirus L1 virus-like particle vaccine in the Costa Rica Vaccine Trial. Cancer Prev Res (Phila) 6: 1242-1250.

51. Safaeian M, Sampson JN, Pan Y, Porras C, Kemp TJ, et al. (2018) Durability of Protection Afforded by Fewer Doses of the HPV16/18 Vaccine: The CVT Trial. J Natl Cancer Inst 110.

52. Gomes AC, Mohsen M, Bachmann MF (2017) Harnessing Nanoparticles for Immunomodulation and Vaccines. Vaccines (Basel) 5.

53. Schiller JT, Lowy DR (2012) Understanding and learning from the success of prophylactic human papillomavirus vaccines. Nat Rev Microbiol 10: 681-692.

54. Longet S, Schiller JT, Bobst M, Jichlinski P, Nardelli-Haefliger D (2011) A murine genital-challenge model is a sensitive measure of protective antibodies against human papillomavirus infection. J Virol 85: 13253-13259.

55. Lowy DR, Herrero R, Hildesheim A, Participants in the INCIwoPEfPHPVVT (2015) Primary endpoints for future prophylactic human papillomavirus vaccine trials: towards infection and immunobridging. Lancet Oncol 16: e226-233.

56. Herrero R, Gonzalez P, Markowitz LE (2015) Present status of human papillomavirus vaccine development and implementation. Lancet Oncol 16: e206-216.

57. Oliver SE, Unger ER, Lewis R, McDaniel D, Gargano JW, et al. (2017) Prevalence of Human Papillomavirus Among Females After Vaccine Introduction-National Health and Nutrition Examination Survey, United States, 2003-2014. J Infect Dis 216: 594-603.

58. Kietpeerakool C, Kleebkaow P, Srisomboon J (2015) Human Papillomavirus Genotype Distribution among Thai Women with High-Grade Cervical Intraepithelial Lesions and Invasive Cervical Cancer: a Literature Review. Asian Pac J Cancer Prev 16: 5153-5158.

59. Kantathavorn N, Mahidol C, Sritana N, Sricharunrat T, Phoolcharoen N, et al. (2015) Genotypic distribution of human papillomavirus (HPV) and cervical cytology findings in 5906 Thai women undergoing cervical cancer screening programs. Infect Agent Cancer 10: 7.
60. Phoolcharoen N, Kantathavorn N, Sricharunrat T, Saeloo S, Krongthong W (2017) A population-based study of cervical cytology findings and human papillomavirus infection in a suburban area of Thailand. Gynecol Oncol Rep 21: 73-77.
61. Tangjitgamol S, Kantathavorn N, Kittisiam T, Chaowawanit W, Phoolcharoen N, et al. (2016) Prevalence and Associated Factors of Abnormal Cervical Cytology and HighRisk HPV DNA among Bangkok Metropolitan Women. Asian Pac J Cancer Prev 17: 3147-3153.
62. Paengchit K, Kietpeerakool C, Lalitwongsa S (2014) Prevalence and genotype distribution of HPV among women attending a cervical cancer screening mobile unit in Lampang, Thailand. Asian Pac J Cancer Prev 15: 6151-6154.
63. https://en.wikipedia.org/wiki/List_of_Thai_provinces_by_GPP.
64. https:/*http://www.fda.gov/downloads/biologicsbloodvaccines/vaccines/approvedproducts/ucm186981.pdf*.
65. Vorsters A, Van Keer S, Van Damme P (2015) The use of urine in the follow-up of HPV vaccine trials. Hum Vaccin Immunother 11: 350-352.
66. Vorsters A, Van Keer S, Biesmans S, Hens A, De Coster I, et al. (2016) Long-Term Follow-up of HPV Infection Using Urine and Cervical Quantitative HPV DNA Testing. Int J Mol Sci 17.
67. Van Keer S, Pattyn J, Tjalma WAA, Van Ostade X, Ieven M, et al. (2017) First-void urine: A potential biomarker source for triage of high-risk human papillomavirus infected women. Eur J Obstet Gynecol Reprod Biol 216: 1-11.
68. Tshomo U, Franceschi S, Tshokey T, Tobgay T, Baussano I, et al. (2017) Evaluation of the performance of Human Papillomavirus testing in paired urine and clinician-collected cervical samples among women aged over 30 years in Bhutan. Virol J 14: 74.
69. Franceschi S, Chantal Umulisa M, Tshomo U, Gheit T, Baussano I, et al. (2016) Urine testing to monitor the impact of HPV vaccination in Bhutan and Rwanda. Int J Cancer 139: 518-526.
70. Franceschi S, Clifford GM, Baussano I (2018) Options for design of real-world impact studies of single-dose vaccine schedules. Vaccine.

71. Nilyanimit P, Chansaenroj J, Karalak A, Laowahutanont P, Junyangdikul P, et al. (2017) Comparison of human papillomavirus (HPV) detection in urine and cervical swab samples using the HPV GenoArray Diagnostic assay. PeerJ 5: e3910.
72. Nilyanimit P, Wanlapakorn N, Niruthisard S, Pohthipornthawat N, Karalak A, et al. (2013) Detection of human papillomavirus in male and female urine by electrochemical DNA chip and PCR sequencing. Asian Pac J Cancer Prev 14: 5519-5525.
73. Park Y, Lee E, Choi J, Jeong S, Kim HS (2012) Comparison of the Abbott RealTime High-Risk Human Papillomavirus (HPV), Roche Cobas HPV, and Hybrid Capture 2 assays to direct sequencing and genotyping of HPV DNA. J Clin Microbiol 50: 2359-2365.
74. Rao A, Young S, Erlich H, Boyle S, Krevolin M, et al. (2013) Development and characterization of the cobas human papillomavirus test. J Clin Microbiol 51: 1478-1484.
75. Stanczuk GA, Currie H, Baxter G, Foster A, Gibson L, et al. (2015) Cobas 4800 HPV detection in the cervical, vaginal and urine samples of women with high-grade CIN before and after treatment. J Clin Pathol 68: 567-570.
76. Nilyanimit P, Chansaenroj J, Poomipak W, Praianantathavorn K, Payungporn S, et al. (2018) Comparison of Four Human Papillomavirus Genotyping Methods: Next-generation Sequencing, INNO-LiPA, Electrochemical DNA Chip, and Nested-PCR. Ann Lab Med 38: 139-146.
77. Panicker G, Rajbhandari I, Gurbaxani BM, Querec TD, Unger ER (2015) Development and evaluation of multiplexed immunoassay for detection of antibodies to HPV vaccine types. J Immunol Methods 417: 107-114.
78. Grabowska K, Wang X, Jacobsson A, Dillner J (2002) Evaluation of cost-precision rations of different strategies for ELISA measurement of serum antibody levels. J Immunol Methods 271: 1-15.
79. Angelo MG, Zima J, Tavares Da Silva F, Baril L, Arellano F (2014) Post-licensure safety surveillance for human papillomavirus-16/18-AS04-adjuvanted vaccine: more than 4 years of experience. Pharmacoepidemiol Drug Saf 23: 456-465.
80. Angelo MG, David MP, Zima J, Baril L, Dubin G, et al. (2014) Pooled analysis of large and long-term safety data from the human papillomavirus-16/18-AS04-adjuvanted vaccine clinical trial programme. Pharmacoepidemiol Drug Saf 23: 466-479.
81. Lehtinen M, Eriksson T, Apter D, Hokkanen M, Natunen K, et al. (2016) Safety of the human papillomavirus (HPV)-16/18 AS04-adjuvanted vaccine in adolescents aged 12-15 years: Interim analysis of a large community-randomized controlled trial. Hum Vaccin Immunother 12: 3177-3185.

17 APPENDIX

1. DATA COLLECTION FORM AND SEXUAL BEHAVIORAL QUESTIONNAIRE

1	Currently, who do you staying with?	1. Father and Mother<br>2. Father or mother only<br>3. Friend<br>4. Boyfriend<br>5. Stay with relatives<br>6. Live alone<br>7. Other, specify ___________
2	Have student ever had sex?	1. Yes<br>2. Never had sex
3.1	If yes, How old when you had first sex?	Age ______ years
3.2	In lifetime, how many male partners did you have sex with?	Specify number of male partner(s) ______
4	In the past 12 months, how many partners did the student have sex with?	1. _______ person(s)<br>2. Never had sex in the past 12 months
5	In the past 12 months, if student ever had sex, did your partner use condom?	1. Every time<br>2. Sometimes<br>3. Never use<br>4. Never use condom in past 12 months
6	How often did you have sex during the past month	Specify number of times ______
7	In the past 12 months, student had sex for money or things in exchange?	1. Never received money or things in exchange<br>2. Received money or things in exchange<br>3. Never had sex in the past 12 months
8	In the past 12 months, have you ever had any symptoms (check all that apply)	1. Had difficulty urination<br>2. Had abnormal discharge from genitalia<br>3. Had pus from genitalia<br>4. Had pus from anus<br>5. Sore at genitalia<br>6. Sore at anus<br>7. Blister/wart at genitalia<br>8. Blister/wart at anus<br>9. Other, specify ___________<br>10. Never had any abnormal symptoms in the past 12 months
